# Supplementary material for: Autocatalytic photoredox Chan-Lam coupling of free diaryl sulfoximines with arylboronic acids
Source: Nat Commun. 2021 Feb 10;12:932. doi: 10.1038/s41467-021-21156-w (PMC7876119; doi:10.1038/s41467-021-21156-w)
Supplement: Supplementary file 1 — Supplementary Information [file 41467_2021_21156_MOESM1_ESM.pdf]

Supplementary Information

Autocatalytic Photoredox Chan-Lam Coupling of Free Diaryl Sulfoximines with Arylboronic Acids

Wang et al.

Supplementary Methods

General Information

All reactions were carried out under dry argon. Anhydrous ethanol (EtOH), methanol (MeOH), tetrahydrofuran (THF), *N*,*N*-dimethyl formamide (DMF), dimethyl sulfoxide (DMSO) were purchased from J&K Chemicals and used without further purification. Chemicals were purchased from Bidepharm, Aladdin, Energy Chemical, Adamas-beta, JiuDing or J&K Chemicals and solvents were purchased from Fisher Scientific. Unless otherwise stated, reagents were commercially available and used as purchased. The progress of the reactions was monitored by thin-layer chromatography using Whatman Partisil K6F 250 μm precoated 60 Å silica gel plates and visualized by short-wave ultraviolet light as well as by treatment with iodine. Flash chromatography was performed with silica gel (200–300 mesh). The NMR spectra were obtained using a Brüker 400 MHz Fourier-transform NMR spectrometer. Chemical shifts are reported in units of parts per million (ppm) downfield from tetramethylsilane (TMS), and all coupling constants are reported in hertz. Infrared spectra were obtained with KBr plates using a Perkin-Elmer Spectrum Vertex 80 spectrometer. Mass spectrometric data were obtained using Bruker Apex IV RTMS. Melting points were determined on a SGWX-4 melting point apparatus and are uncorrected. Ultraviolet-Visible (UV) spectrophotometer was detected by Hitachi dual-beam UH5300. Resonance (EPR) spectra were recorded on a Bruker ELEXSYS II E 500 EPR spectrometer, and gas chromatography (GC) used a Fuli GC 9790 plus. Luminescence emission intensities were recorded using FS5 Spectrofluorometer from Edinburgh Instruments.

Preparation of *NH*-Diaryl Sulfoximines: Sulfoximines were prepared according to the literature procedures.[1]

Procedure and Characterization for Copper-Catalyzed *NH*-Diaryl Sulfoximines with Arylboronic Acids.[2]

General Procedure for Catalysis: To an oven-dried microwave vial equipped with a stir bar was added sulfoximine **1** (0.75 mmol, 1.0 equiv), boronic acid **2** (1.5 mmol, 2.0 equiv), $Cu(O_2CCF_3)_2{\cdot}H_2O$ (21.7 mg, 0.075 mmol, 10 mol %) under an argon atmosphere in a dry box. The vial was capped with a septum and removed from the dry box. EtOH (0.5 mL) was added into the reaction vial via syringe, and the reaction solution was stirred at room temperature under argon with ambient light for 48 h. Upon completion of the reaction, the vial was opened to air, and the reaction mixture was passed through a short pad of silica gel. The pad was then rinsed with 100:1 DCM:MeOH (20 mL). The solvent was removed under reduced pressure. The residue was purified by flash chromatography as outlined below to afford the purified product.

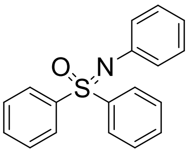

***N*-Phenyl-*S*,*S*-diphenylsulfoximine (3aa):** The reaction was performed following the General Procedure with **1a** (162.8 mg, 0.75 mmol) and **2a** (183.1 mg, 1.5 mmol). The crude product was purified by flash chromatography on silica gel (eluted with hexane:EtOAc = 5:1) to give the product **3aa** (208.8 mg, 95% yield) as a colorless solid. R_f = 0.3 (hexane:EtOAc = 5:1). The spectroscopic data match the previously reported data.[3]

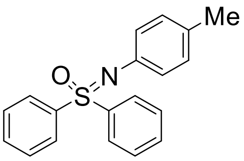

***N*-(4-Methylphenyl)-*S*,*S*-diphenylsulfoximine (3ab):** The reaction was performed following the General Procedure with **1a** (162.8 mg, 0.75 mmol) and **2b** (204.1 mg, 1.5 mmol). The crude product was purified by flash chromatography on silica gel (eluted with hexane:EtOAc = 5:1) to give the product **3ab** (221.1 mg, 96% yield) as a colorless solid. R_f = 0.3 (hexane:EtOAc = 5:1). The spectroscopic data match the previously reported data.[3]

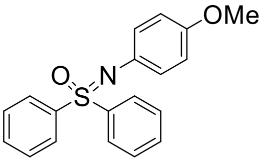

***N*-(4-Methoxyphenyl)-*S*,*S*-diphenylsulfoximine (3ac):** The reaction was performed following the General Procedure with **1a** (162.8 mg, 0.75 mmol) and **2c** (228.1 mg, 1.5 mmol). The crude product was purified by flash chromatography on silica gel (eluted with hexane:EtOAc = 3:1) to give the product **3ac** (206.2 mg, 85% yield) as a colorless solid. R_f = 0.3 (hexane:EtOAc = 3:1). The spectroscopic data match the previously reported data.[4]

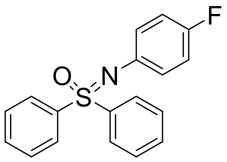

***N*-(4-Fluorophenyl)-*S*,*S*-diphenylsulfoximine (3ad):** The reaction was performed following the General Procedure with **1a** (162.8 mg, 0.75 mmol) and **2d** (210.1 mg, 1.5 mmol). The crude product was purified by flash chromatography on silica gel (eluted with hexane:EtOAc = 5:1) to give the product **3ad** (193.6 mg, 85% yield) as a colorless solid. R_f = 0.3 (hexane:EtOAc = 5:1). The spectroscopic data match the previously reported data.[3]

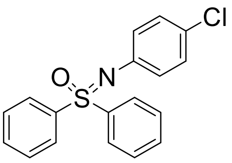

***N*-(4-Chlorophenyl)-*S*,*S*-diphenylsulfoximine (3ae):** The reaction was performed following the General Procedure with **1a** (162.8 mg, 0.75 mmol) and **2e** (234.0 mg, 1.5 mmol). The crude product was purified by flash chromatography on silica gel (eluted with hexane:EtOAc = 5:1) to give the

product **3ae** (223.2 mg, 85% yield) as a colorless solid. R_f = 0.3 (hexane:EtOAc = 5:1). The spectroscopic data match the previously reported data.[3]

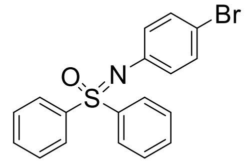

***N*-(4-Bromophenyl)-*S,S*-diphenylsulfoximine (3af):** The reaction was performed following the General Procedure with **1a** (162.8 mg, 0.75 mmol) and **2f** (299.9 mg, 1.5 mmol). The crude product was purified by flash chromatography on silica gel (eluted with hexane:EtOAc = 5:1) to give the product **3af** (250.4 mg, 90% yield) as a colorless solid. R_f = 0.3 (hexane:EtOAc = 5:1). The spectroscopic data match the previously reported data.[3]

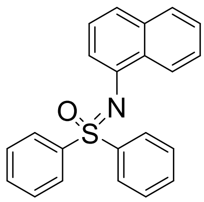

***N*-(1-Naphthalen)-*S,S*-diphenylsulfoximine (3ag):** The reaction was performed following the General Procedure with **1a** (162.8 mg, 0.75 mmol) and **2g** (258.1 mg, 1.5 mmol). The crude product was purified by flash chromatography on silica gel (eluted with hexane:EtOAc = 5:1) to give the product **3ag** (193.0 mg, 75% yield) as a colorless solid. R_f= 0.3 (hexane:EtOAc = 5:1). m.p. = 168-170 °C; ^{1}H NMR (400 MHz, $CDCl_3$) δ 8.79 (d, J = 8.0 Hz, 1H), 8.16 (d, J = 7.6 Hz, 4H), 7.83 (d, J = 8.0 Hz, 1H), 7.61 (t, J = 7.6 Hz, 1H), 7.57 – 7.45 (m, 7H), 7.43 (d, J = 7.6 Hz, 1H), 7.26 – 7.17 (m, 2H) ppm; ^{13}C{^{1}H}NMR (100 MHz, $CDCl_3$) δ 141.2, 141.0, 134.6, 132.8, 130.4, 129.4, 128.5, 127.9, 127.6, 126.4, 126.1, 125.9, 125.1, 124.0, 121.6, 120.4, 117.0, 108.6 ppm; IR (thin film): 3048, 1571, 1503, 1390, 1278, 1222, 1115, 972, 727, 681 cm^{-1}; HRMS calculated for $C_{22}H_{18}NOS$ 344.1104, found 344.1101 $[M+H]^+$.

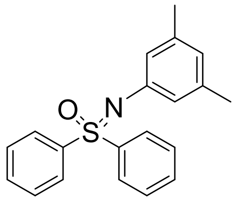

***N*-(3,5-Dimethylphenyl)-*S,S*-diphenylsulfoximine (3ah):** The reaction was performed following the General Procedure with **1a** (162.8 mg, 0.75 mmol) and **2h** (225.1 mg, 1.5 mmol). The crude product was purified by flash chromatography on silica gel (eluted with hexane:EtOAc = 5:1) to give the product **3ah** (192.7 mg, 80% yield) as a colorless solid. R_f = 0.3 (hexane:EtOAc = 5:1). m.p. = 196-197 °C; ^{1}H NMR (400 MHz, $CDCl_3$) δ 8.05 (m, 4H), 7.51 – 7.39 (m, 6H), 6.79 (s, 2H), 6.53 (s, 1H), 2.17 (s, 6H) ppm; ^{13}C{^{1}H} NMR (100 MHz, $CDCl_3$) δ 144.3, 141.1, 138.4, 132.6, 129.3, 128.5, 123.7, 121.5, 21.3 ppm; IR (thin film): 3448, 3421, 2914, 1591, 1440, 1319, 1225, 1173, 1094, 557 cm^{-1}; HRMS calculated for $C_{20}H_{20}NOS$ 322.1260, found 322.1258 $[M+H]^+$.

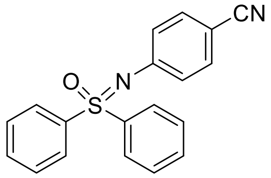

***N*-(4-Cyanophenyl)-*S,S*-diphenylsulfoximine (3ai):** The reaction was performed following the General Procedure with **1a** (163.0 mg, 0.75 mmol) and **2i** (220.4 mg, 1.5 mmol). The crude product was purified by flash

chromatography on silica gel (eluted with hexane:EtOAc = 3:1) to give the product **3ai** (140.2 mg, 59% yield) as a colorless solid. R_f = 0.3 (hexane:EtOAc = 3:1). m.p. = 155-156 °C; ^{1}H NMR (600 MHz, $CDCl_3$) *δ* 8.06 – 8.00 (m, 4H), 7.58 – 7.54 (m, 2H), 7.53 – 7.48 (m, 4H), 7.43 – 7.39 (m, 2H), 7.18 – 7.14 (m, 2H) ppm; ^{13}C{^{1}H}NMR (150 MHz, $CDCl_3$) δ 149.9, 140.1, 133.2(2), 133.2(1), 129.6, 128.3, 123.8, 119.7, 104.2 ppm; IR (thin film): 2924, 2850, 2228, 1599, 1497, 1446, 1285, 1213, 1087, 851, 682 cm^{-1}; HRMS calculated for $C_{19}H_{15}N_2OS$ 319.0900, found 319.0900 $[M+H]^+$.

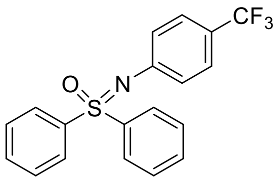

***N*-(4-(Trifluoromethyl)phenyl)-*S*,*S*-diphenylsulfoximine (3aj):** The reaction was performed following the General Procedure with **1a** (163.0 mg, 0.75 mmol) and **2j** (284.9 mg, 1.5 mmol) for 72 h. The crude product was purified by flash chromatography on silica gel (eluted with hexane:EtOAc = 3:1) to give the product **3aj** (221.5 mg, 82% yield) as a colorless solid. R_f = 0.3 (hexane:EtOAc = 3:1). m.p. = 128-129 °C; ^{1}H NMR (600 MHz, $CDCl_3$) *δ* 8.09 – 8.01 (m, 4H), 7.56 – 7.51 (m, 2H), 7.51 – 7.46 (m, 4H), 7.38 (d, *J* = 8.0 Hz, 2H), 7.23 – 7.17 (m, 2H) ppm; ^{13}C{^{1}H}NMR (150 MHz, $CDCl_3$) δ 148.4, 140.4, 133.0, 129.5, 128.4, 126.2 (q, $J_{C\text{-}F}$ = 3.7 Hz), 125.6 (q, $J_{C\text{-}F}$ = 269.2 Hz), 123.4, 123.3 (q, $J_{C\text{-}F}$ = 32.1 Hz) ppm; IR (thin film): 2928, 2856, 1610, 1511, 1309, 1207, 1106, 993, 893, 726, 624 cm^{-1}; HRMS calculated for $C_{19}H_{15}F_3NOS$ 362.0821, found 362.0820 $[M+H]^+$.

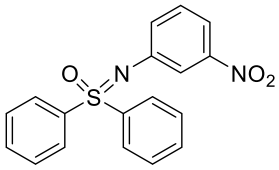

***N*-(3-Nitrophenyl)-*S*,*S*-diphenylsulfoximine (3ak):** The reaction was performed following the General Procedure with **1a** (163.0 mg, 0.75 mmol) and **2k** (250.4mg, 1.5 mmol). The crude product was purified by flash chromatography on silica gel (eluted with hexane:EtOAc = 3:1) to give the product **3ak** (168.2 mg, 66% yield) as a colorless solid. R_f = 0.3 (hexane:EtOAc = 3:1). m.p. = 173-174 °C; ^{1}H NMR (600 MHz, $CDCl_3$) *δ* 8.09 – 8.03 (m, 4H), 7.97 (t, *J* = 2.1 Hz, 1H), 7.74 – 7.70 (m, 1H), 7.57 – 7.54 (m, 2H), 7.53 – 7.48 (m, 4H), 7.46 – 7.41 (m, 1H), 7.25 (t, *J* = 8.1 Hz, 1H) ppm; ^{13}C{^{1}H}NMR (150 MHz, $CDCl_3$) δ 148.9, 146.3, 140.0, 133.2, 129.5(3), 129.4(6), 129.3(6), 128.4, 118.4, 116.4 ppm; IR (thin film): 3095, 3081, 1523, 1344, 1252, 1204, 1087, 1014, 877, 799, 728, 624 cm^{-1}; HRMS calculated for $C_{18}H_{15}N_2O_3S$ 339.0798, found 339.0798 $[M+H]^+$.

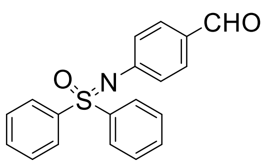

***N*-(4- Benzaldehyde)-*S*,*S*-diphenylsulfoximine (3al):** The reaction was performed following the General Procedure with **1a** (162.8 mg, 0.75 mmol) and **2l** (225.1 mg, 1.5 mmol). The crude product was purified by flash chromatography on silica gel (eluted with hexane:EtOAc = 5:1) to give the product **3al** (192.6 mg, 80% yield) as a colorless solid. R_f = 0.3 (hexane:EtOAc = 5:1). m.p. =

141-142 °C; ^{1}H NMR (400 MHz, $CDCl_3$) *δ* 9.80 (s, 1H), 8.10 – 8.01 (m, 4H), 7.67 (d, *J* = 8.5 Hz, 2H), 7.58 – 7.46 (m, 6H), 7.27 – 7.20 (m, 2H) ppm; ^{13}C{^{1}H}NMR (100 MHz, $CDCl_3$) *δ* 191.1, 151.8, 140.3, 133.2, 131.3, 130.2, 129.5, 128.4, 123.5 ppm; IR (thin film): 3431, 3414, 2922, 1674, 1595, 1319, 1269, 1206, 1159, 1088, 747, 523 cm^{-1}; HRMS calculated for $C_{19}H_{16}NO_2S$ 322.0896, found 322.0892 $[M+H]^+$.

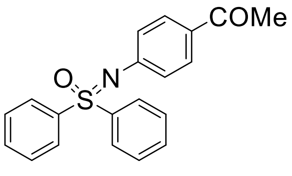

***N*-(4-Acetylphenyl)-*S*,*S*-diphenylsulfoximine (3am):** The reaction was performed following the General Procedure with **1a** (162.8 mg, 0.75 mmol) and **2m** (246.1 mg, 1.5 mmol). The crude product was purified by flash chromatography on silica gel (eluted with hexane:EtOAc = 5:1) to give the product **3am** (196.0 mg, 78% yield) as a colorless solid. R_f = 0.3 (hexane:EtOAc = 5:1). m.p. = 140-142 °C; ^{1}H NMR (400 MHz, $CDCl_3$) *δ* 8.09 – 8.01 (m, 4H), 7.77 (dd, *J* = 8.5, 1.4 Hz, 2H), 7.56 – 7.44 (m, 6H), 7.17 (d, *J* = 8.5 Hz, 2H), 2.48 (s, 3H) ppm; ^{13}C{^{1}H}NMR (100 MHz, $CDCl_3$) *δ* 197.1, 150.3, 140.4, 133.1, 130.7, 129.8, 129.5, 128.4, 123.1, 26.3 ppm; IR (thin film): 2361, 2160, 1689, 1590, 1352, 1261, 1179, 994, 828, 634 cm^{-1}; HRMS calculated for $C_{20}H_{18}NO_2S$ 336.1053, found 336.1056 $[M+H]^+$.

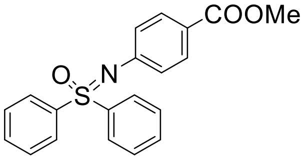

***N*-(4-Benzoate)-*S*,*S*-diphenylsulfoximine (3an):** The reaction was performed following the General Procedure with **1a** (162.8 mg, 0.75 mmol) and **2n** (270.1 mg, 1.5 mmol). The crude product was purified by flash chromatography on silica gel (eluted with hexane:EtOAc = 5:1) to give the product **3an** (158.1 mg, 60% yield) as a colorless solid. R_f = 0.3 (hexane:EtOAc = 5:1). m.p. = 196-197 °C; ^{1}H NMR (400 MHz, $CDCl_3$) *δ* 8.07 – 8.02 (m, 4H), 7.86 – 7.79 (m, 2H), 7.56 – 7.45 (m, 6H), 7.17 – 7.13 (m, 2H), 3.83 (s, 3H) ppm; ^{13}C{^{1}H}NMR (100 MHz, $CDCl_3$) *δ* 167.2, 149.9, 140.4, 133.0, 130.8, 129.4, 128.4, 123.1, 51.8 ppm; IR (thin film): 3483, 1748, 1673, 1439, 1255, 1090, 1000, 553 cm^{-1}; HRMS calculated for $C_{20}H_{18}NO_3S$ 352.1002, found 352.1000 $[M+H]^+$.

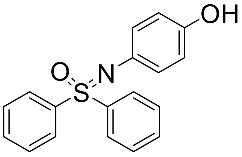

***N*-(4-Hydroxyphenyl)-*S*,*S*-diphenylsulfoximine (3ao):** The reaction was performed following the General Procedure with **1a** (162.8 mg, 0.75 mmol) and **2o** (207.1 mg, 1.5 mmol). The crude product was purified by flash chromatography on silica gel (eluted with hexane:EtOAc = 2:1) to give the product **3ao** (162.3 mg, 70% yield) as a colorless solid. R_f = 0.3 (hexane:EtOAc = 2:1). m.p. = 110-111 °C; ^{1}H NMR (400 MHz, $CDCl_3$) *δ* 8.05 – 7.98 (m, 4H), 7.53 – 7.38 (m, 6H), 7.02 – 6.95 (m, 2H), 6.62 – 6.57 (m, 2H), 5.25 (br s, 1H) ppm; ^{13}C{^{1}H}NMR (100 MHz, $CDCl_3$) *δ* 152.3, 140.5, 137.1,

132.7, 129.3, 128.6, 127.9, 124.9 ppm; IR (thin film): 3483, 3266, 1669, 1457, 1224, 1126, 1093, 958, 764, 720, 683 cm^{-1}; HRMS calculated for $C_{18}H_{16}NO_2S$ 310.0896, found 310.0895 $[M+H]^+$.

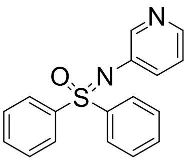

N**-(3-Pyridin)-*****S,S*****-diphenylsulfoximine (3ap):** The reaction was performed following the General Procedure with **1a** (162.8 mg, 0.75 mmol) and **2p** (184.6 mg, 1.5 mmol). The crude product was purified by flash chromatography on silica gel (eluted with hexane:EtOAc = 3:1) to give the product **3ap** (165.4 mg, 75% yield) as a colorless solid. R_f = 0.2 (hexane:EtOAc = 3:1). m.p. =151-152 °C; ^{1}H NMR (400 MHz, $CDCl_3$) δ 8.47 (s, 1H), 8.11 (t, J = 7.8 Hz, 1H), 8.05 (d, J = 7.1 Hz, 4H), 7.60 – 7.44 (m, 6H), 7.41 (d, J = 8.1 Hz, 1H), 7.08 – 7.00 (m, 1H) ppm; $^{13}C\{^1H\}$NMR (100 MHz, $CDCl_3$) δ 145.7, 142.7, 141.6, 140.2, 133.1, 129.9, 129.5, 128.5, 123.6 ppm; IR (thin film): 3048, 1571, 1446, 1390, 1331, 1278, 1245, 1169, 1079, 1015, 869, 754 cm^{-1}; HRMS calculated for $C_{17}H_{15}N_2OS$ 295.0904, found 295.0898 $[M+H]^+$.

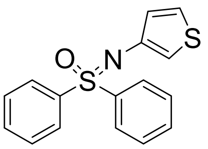

N**-(3-Thiophen)-*****S,S*****-diphenylsulfoximine (3aq):** The reaction was performed following the General Procedure with **1a** (162.8 mg, 0.75 mmol) and **2q** (192.0 mg, 1.5 mmol). The crude product was purified by flash chromatography on silica gel (eluted with hexane:EtOAc = 3:1) to give the product **3aq** (145.8 mg, 65% yield) as a colorless solid. R_f = 0.3 (hexane:EtOAc = 3:1). m.p. =160-161 °C; ^{1}H NMR (400 MHz, $CDCl_3$) δ 8.04 (d, J = 7.5 Hz, 4H), 7.54 – 7.43 (m, 6H), 7.11 – 7.06 (m, 1H), 6.92 (d, J = 4.8 Hz, 1H), 6.58 (s, 1H) ppm; $^{13}C\{^1H\}$NMR (100 MHz, $CDCl_3$) δ 142.2, 140.5, 132.8, 129.3, 128.6, 125.7, 124.1, 109.5 ppm; IR (thin film): 3435, 3414, 1514, 1248, 1169, 1086, 1013, 772, 683, 550 cm^{-1}; HRMS calculated for $C_{16}H_{14}NOS_2$ 300.0511, found 300.0509 $[M+H]^+$.

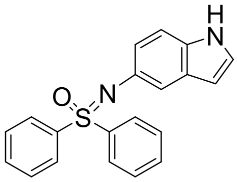

N**-(5-Indolyl)-*****S,S*****-diphenylsulfoximine (3ar):** The reaction was performed following the General Procedure with **1a** (162.8 mg, 0.75 mmol) and **2r** (241.6 mg, 1.5 mmol). The crude product was purified by flash chromatography on silica gel (eluted with hexane:EtOAc = 1:1) to give the product **3ar** (161.9 mg, 65% yield) as a colorless solid. R_f = 0.3 (hexane:EtOAc = 1:1). m.p. =103-104 °C; ^{1}H NMR (400 MHz, $CDCl_3$) δ 8.14 – 7.99 (m, 5H), 7.41 (s, 7H), 7.16 – 7.04 (m, 2H), 7.01 (d, J = 2.4 Hz, 1H), 6.34 (br s, 1H) ppm; $^{13}C\{^1H\}$NMR (100 MHz, $CDCl_3$) δ 141.1, 136.9, 132.5, 132.1, 129.2, 128.7, 128.5, 124.5, 120.1, 114.6, 111.3, 102.1 ppm; IR (thin film): 2361, 2160, 1460, 1234, 1159, 1086, 1014, 723, 695 cm^{-1}; HRMS calculated for $C_{20}H_{17}N_2OS$ 333.1056, found 333.1053 $[M+H]^+$.

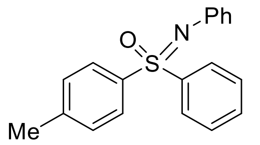

***N*-Phenyl-*S*-phenyl-*S*-(4-methylphenyl)sulfoximine (3ba):** The reaction was performed following the General Procedure with **1b** (173.3 mg, 0.75 mmol) and **2a** (183.1 mg, 1.5 mmol). The crude product was purified by flash chromatography on silica gel (eluted with hexane:EtOAc = 5:1) to give the product **3ba** (218.8 mg, 95% yield) as a colorless solid. R_f = 0.3 (hexane:EtOAc = 5:1). The spectroscopic data match the previously reported data.[3]

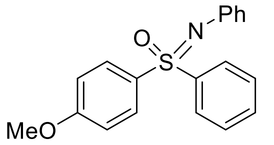

***N*-Phenyl-*S*-phenyl-*S*-(4-methoxyphenyl)sulfoximine (3ca):** The reaction was performed following the General Procedure with **1c** (185.3 mg, 0.75 mmol) and **2a** (183.1 mg, 1.5 mmol). The crude product was purified by flash chromatography on silica gel (eluted with hexane:EtOAc = 3:1) to give the product **3ca** (184.2 mg, 76% yield) as a colorless solid. R_f = 0.3 (hexane:EtOAc = 3:1). m.p. =107-108 °C; ^{1}H NMR (400 MHz, $CDCl_3$) δ 8.05 – 7.92 (m, 4H), 7.41 – 7.40 (m, 3H), 7.19 – 7.05 (m, 4H), 6.93 – 6.80 (m, 3H), 3.73 (s, 3H) ppm; $^{13}C\{^1H\}$NMR (100 MHz, $CDCl_3$) δ 163.1, 144.9, 141.6, 132.4, 131.9, 130.8, 129.3, 129.0, 128.2, 123.8, 121.6, 114.6, 55.6 ppm. IR (thin film): 3048, 1571, 1446, 1390, 1245, 1079, 1015, 972, 799, 727, 681 cm^{-1}; HRMS calculated for $C_{19}H_{18}NO_2S$ 324.1053, found 324.1051 $[M+H]^+$.

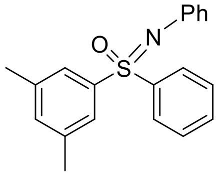

***N*-Phenyl-*S*-phenyl-*S*-(3,5-dimethylphenyl)sulfoximine (3da):** The reaction was performed following the General Procedure with **1d** (183.8 mg, 0.75 mmol) and **2a** (183.1 mg, 1.5 mmol). The crude product was purified by flash chromatography on silica gel (eluted with hexane:EtOAc = 5:1) to give the product **3da** (180.6 mg, 75% yield) as a colorless solid. R_f = 0.3 (hexane:EtOAc = 5:1). m.p.= 100-102 °C; ^{1}H NMR (400 MHz, $CDCl_3$) δ 8.08 – 7.94 (m, 2H), 7.68 – 7.65 (m, 2H), 7.43 – 7.36 (m, 3H), 7.19 – 7.07 (m, 4H), 7.05 (s, 1H), 6.87 – 6.81 (m, 1H), 2.28 (s, 6H) ppm; $^{13}C\{^1H\}$NMR (100 MHz, $CDCl_3$) δ 144.8, 141.1, 140.6, 139.3, 134.5, 132.5, 129.3, 129.1, 128.5, 126.0, 123.8, 121.6, 21.3 ppm; IR (thin film): 3468, 3129, 2717, 1581, 1465, 1389, 1228, 1174, 1094, 550 cm^{-1}; HRMS calculated for $C_{20}H_{20}NOS$ 322.1260, found 322.1265 $[M+H]^+$.

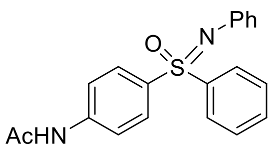

***N*-Phenyl-*S*-phenyl-*S*-(4-acetamide)sulfoximine (3ea):** The reaction was performed following the General Procedure with **1e** (205.6 mg, 0.75 mmol) and **2a** (183.1 mg, 1.5 mmol). The crude product was purified by flash chromatography on silica gel (eluted with hexane:EtOAc = 5:1) to give the

product **3ea** (165.4 mg, 63% yield) as a colorless solid. R_f = 0.3 (hexane:EtOAc = 5:1). m.p.= 111-112 °C; ^{1}H NMR (400 MHz, $CDCl_3$) δ 8.01 (dd, J = 9.4, 7.9 Hz, 2H), 7.95 (d, J = 8.7 Hz, 3H), 7.61 (d, J = 8.7 Hz, 2H), 7.54 – 7.42 (m, 3H), 7.18 – 7.12 (m, 4H), 6.93 – 6.86 (m, 1H), 2.12 (s, 3H) ppm. ^{13}C{^{1}H}NMR (100 MHz, $CDCl_3$) δ 168.8, 144.6, 142.1, 140.9, 135.1, 132.7, 129.8, 129.3, 129.0, 128.4, 123.8, 121.8, 119.7, 24.6 ppm; IR (thin film): 3356, 2922, 2361, 1542, 1521, 1397, 1264, 1102, 832, 762 cm^{-1}; HRMS calculated for $C_{20}H_{19}N_2O_2S$ 351.1162, found 351.1162 [M+H]$^+$.

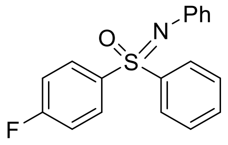

***N*-Phenyl-*S*-phenyl-*S*-(4-fluorophenyl)sulfoximine (3fa):** The reaction was performed following the General Procedure with **1f** (176.3 mg, 0.75 mmol) and **2a** (183.1 mg, 1.5 mmol). The crude product was purified by flash chromatography on silica gel (eluted with hexane:EtOAc = 5:1) to give the product **3fa** (186.6 mg, 80% yield) as a colorless solid. R_f = 0.3 (hexane:EtOAc = 5:1). m.p. = 111-113 °C; ^{1}H NMR (400 MHz, $CDCl_3$) δ 8.14 – 8.03 (m, 4H), 7.58 – 7.45 (m, 3H), 7.21 – 7.11 (m, 6H), 6.97 – 6.88 (m, 1H) ppm; ^{13}C{^{1}H}NMR (100 MHz, $CDCl_3$) δ ^{13}C{^{1}H}NMR (100 MHz, $CDCl_3$) δ 165.2 (d, $J_{C\text{-}F}$ = 255.3 Hz), 144.4, 140.1, 136.8 (d, $J_{C\text{-}F}$ = 3.2 Hz), 132.8, 131.3 (d, $J_{C\text{-}F}$ = 9.5 Hz), 129.4, 129.1, 128.5, 123.7, 121.9, 116.6 (d, $J_{C\text{-}F}$ = 22.6 Hz) ppm; IR (thin film): 3443, 3065, 1585, 1485, 1298, 1194, 1092, 999, 755, 692 cm^{-1}; HRMS calculated for $C_{18}H_{15}NOFS$ 312.0853, found 312.0849 [M+H]$^+$.

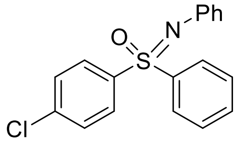

***N*-Phenyl-*S*-phenyl-*S*-(4-chlorophenyl)sulfoximine (3ga):** The reaction was performed following the General Procedure with **1g** (188.3 mg, 0.75 mmol) and **2a** (183.1 mg, 1.5 mmol). The crude product was purified by flash chromatography on silica gel (eluted with hexane:EtOAc = 5:1) to give the product **3ga** (218.3 mg, 89% yield) as a colorless solid. R_f = 0.3 (hexane:EtOAc = 5:1). m.p.= 101-102 °C; ^{1}H NMR (400 MHz, $CDCl_3$) δ 8.08 (d, J = 7.6 Hz, 2H), 8.00 (d, J = 8.5 Hz, 2H), 7.58 – 7.47 (m, 3H), 7.45 (d, J = 8.5 Hz, 2H), 7.21 – 7.14 (m, 4H), 6.96 – 6.89 (m, 1H) ppm; ^{13}C{^{1}H}NMR (100 MHz, $CDCl_3$) δ 144.3, 140.6, 139.5, 139.4, 132.9, 130.1, 129.6, 129.4, 129.1, 128.5, 123.8, 122.0 ppm; IR (thin film): 3353, 3169, 1775, 1381, 1278, 1104, 1085, 979, 756, 663 cm^{-1}; HRMS calculated for $C_{18}H_{15}NOSCl$ 328.0557, found 328.0552 [M+H]$^+$.

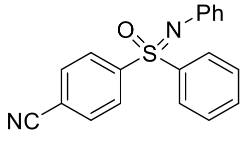

***N*-Phenyl-*S*-phenyl-*S*-(4-cyanophenyl)sulfoximine (3ha):** The reaction was performed following the General Procedure with **1h** (181.7 mg, 0.75 mmol) and **2a** (182.9 mg, 1.5 mmol). The crude product was purified by flash chromatography on silica gel (eluted with hexane:EtOAc = 3:1) to give the product **3ha** (168.3 mg,

71% yield) as a colorless solid. R_f = 0.3 (hexane:EtOAc = 3:1). m.p. = 143-144 °C; ^{1}H NMR (600 MHz, $CDCl_3$) δ 8.17 – 8.13 (m, 2H), 8.09 – 8.04 (m, 2H), 7.77 – 7.72 (m, 2H), 7.60 – 7.55 (m, 1H), 7.55 – 7.49 (m, 2H), 7.19 – 7.14 (m, 2H), 7.14 – 7.09 (m, 2H), 6.96 – 6.89 (m, 1H) ppm; ^{13}C{^{1}H}NMR (150 MHz, $CDCl_3$) δ 145.6, 143.7, 139.6, 133.4, 133.0, 129.6, 129.2(0), 129.1(8), 128.8, 123.7, 122.3, 117.3, 116.4 ppm; IR (thin film): 3089, 2923, 2232, 1595, 1493, 1444, 1310, 748, 649 cm^{-1}; HRMS calculated for $C_{19}H_{15}N_2OS$ 319.0900, found 319.0898 $[M+H]^+$.

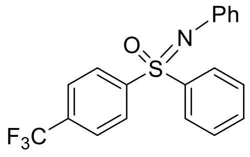

***N*-Phenyl-*S*-phenyl-*S*-(4-trifluoromethyl)sulfoximine (3ia):** The reaction was performed following the General Procedure with **1i** (213.8 mg, 0.75 mmol) and **2a** (183.1 mg, 1.5 mmol). The crude product was purified by flash chromatography on silica gel (eluted with hexane:EtOAc = 5:1) to give the product **3ia** (211.2 mg, 78% yield) as a colorless solid. R_f = 0.3 (hexane:EtOAc = 5:1). m.p.= 123-124 °C; ^{1}H NMR (400 MHz, $CDCl_3$) δ 8.20 (d, J = 8.3 Hz, 2H), 8.11 (d, J = 7.3 Hz, 2H), 7.74 (d, J = 8.3 Hz, 2H), 7.61 – 7.49 (m, 3H), 7.22 – 7.15 (m, 4H), 6.99 – 6.91 (m, 1H) ppm; ^{13}C{^{1}H}NMR (100 MHz, $CDCl_3$) δ 144.8, 144.0, 140.0, 134.3 (q, $J_{C\text{-}F}$ = 33.1 Hz), 133.2, 129.5, 129.2, 129.1, 128.7, 126.4 (q, $J_{C\text{-}F}$ = 3.5 Hz), 123.7, 123.2 (q, $J_{C\text{-}F}$ = 272.9 Hz), 122.1 ppm; IR (thin film): 3443, 3079, 1591, 1498, 1314, 1186, 1113, 700, 532 cm^{-1}; HRMS calculated for $C_{19}H_{15}NOF_3S$ 362.0821, found 362.0814 $[M+H]^+$.

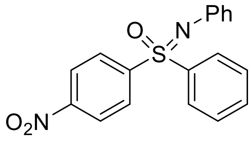

***N*-Phenyl-*S*-phenyl-*S*-(4-nitrophenyl)sulfoximine (3ja):** The reaction was performed following the General Procedure with **1j** (196.7 mg, 0.75 mmol) and **2a** (182.9 mg, 1.5 mmol). The crude product was purified by flash chromatography on silica gel (eluted with hexane:EtOAc = 3:1) to give the product **3ja** (212.9 mg, 89% yield) as a yellow solid. R_f = 0.3 (hexane:EtOAc = 3:1). m.p. = 157-158 °C; ^{1}H NMR (400 MHz, $CDCl_3$) δ 8.32 – 8.25 (m, 2H), 8.23 – 8.17 (m, 2H), 8.13 – 8.05 (m, 2H), 7.61 – 7.48 (m, 3H), 7.19 – 7.10 (m, 4H), 6.96 – 6.88 (m, 1H) ppm; ^{13}C{^{1}H}NMR (100 MHz, $CDCl_3$) δ 150.1, 147.2, 143.6, 139.5, 133.5, 129.9, 129.7, 129.2, 128.8, 124.4, 123.7, 122.4 ppm; IR (thin film): 2950, 2910, 1596, 1547, 1445, 1345, 1182, 730, 635 cm^{-1}; HRMS calculated for $C_{18}H_{15}N_2O_3S$ 339.0798, found 339.0796 $[M+H]^+$.

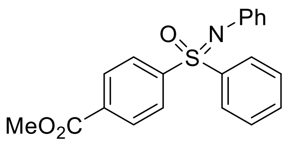

***N*-Phenyl-*S*-phenyl-*S*-(4benzoate)sulfoximine (3ka):** The reaction was performed following the General Procedure with **1k** (206.5 mg, 0.75 mmol) and **2a** (182.9 mg, 1.5 mmol). The crude product was purified by flash chromatography on silica gel (eluted with hexane:EtOAc = 3:1) to give the product **3ka** (243.3 mg, 92% yield) as a colorless solid. R_f= 0.3 (hexane:EtOAc = 3:1). The spectroscopic data match the

previously reported data.[3]

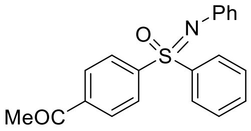

***N*-Phenyl-*S*-phenyl-*S*-(4-acetylphenyl)sulfoximine (3la):** The reaction was performed following the General Procedure with **1l** (194.3 mg, 0.75 mmol) and **2a** (183.1 mg, 1.5 mmol). The crude product was purified by flash chromatography on silica gel (eluted with hexane:EtOAc = 5:1) to give the product **3la** (201.1 mg, 80% yield) as a colorless solid. R_f = 0.3 (hexane:EtOAc = 5:1). m.p.= 103-104 °C; ^{1}H NMR (400 MHz, $CDCl_3$) δ 8.12 (d, J = 8.5 Hz, 2H), 8.08 (dd, J = 8.1, 1.3 Hz, 2H), 7.99 (d, J = 8.5 Hz, 2H), 7.56 – 7.44 (m, 3H), 7.13 (dd, J = 10.7, 2.5 Hz, 4H), 6.93 – 6.84 (m, 1H), 2.57 (s, 3H) ppm; ^{13}C{^{1}H}NMR (100 MHz, $CDCl_3$) δ 200.6, 145.0, 144.1, 140.1, 139.9, 133.1, 129.5, 129.2, 129.1, 128.9, 128.7, 123.7, 122.1, 26.9 ppm; IR (thin film): 3048, 1571, 1503, 1446, 1390, 1278, 1222, 1169, 1115, 972, 779, 727, 681 cm^{-1}; HRMS calculated for $C_{20}H_{18}NO_2S$ 336.1053, found 336.1056 $[M+H]^+$.

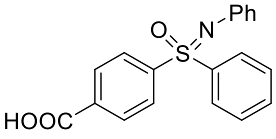

***N*-Phenyl-*S*-phenyl-*S*-(4-carboxyphenyl)sulfoximine (3ma):** The reaction was performed following the General Procedure with **1m** (196.0 mg, 0.75 mmol) and **2a** (182.9 mg, 1.5 mmol) in EtOH (1.5 mL) for 72 h. The crude product was purified by flash chromatography on silica gel (eluted with DCM:MeOH = 10:1) to give the product **3ma** (221.4 mg, 88% yield) as a colorless solid. R_f = 0.3 (DCM:MeOH = 10:1). m.p. = 193-194 °C; ^{1}H NMR (600 MHz, CD_3OD) δ 8.18 – 8.04 (m, 6H), 7.62 – 7.58 (m, 1H), 7.57 – 7.53 (m, 2H), 7.17 – 7.07 (m, 4H), 6.90 – 6.84 (m, 1H) ppm; ^{13}C{^{1}H}NMR (150 MHz, CD_3OD)δ 166.9, 144.3, 139.8, 135.3, 133.2, 130.2, 129.5, 129.3, 128.7, 128.6, 128.5, 123.6, 121.9 ppm; IR (thin film): 3433, 2930, 2860, 1706, 1486, 1266, 1177, 1087, 993, 785, 692, 614 cm^{-1}; HRMS calculated for $C_{19}H_{16}NO_3S$ 338.0845, found 338.0843 $[M+H]^+$.

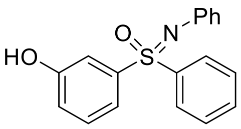

***N*-Phenyl-*S*-phenyl-*S*-(3-hydroxyphenyl)sulfoximine (3na):** The reaction was performed following the General Procedure with **1n** (175.0 mg, 0.75 mmol) and **2a** (182.9 mg, 1.5 mmol). The crude product was purified by flash chromatography on silica gel (eluted with hexane:EtOAc = 1:1) to give the product **3na** (190.3 mg, 82% yield) as a colorless solid. R_f = 0.3 (hexane:EtOAc = 1:1). m.p. = 159-160 °C; ^{1}H NMR (400 MHz, $CDCl_3$) δ 8.03 – 8.00 (m, 2H), 7.67 – 7.66 (m, 1H), 7.57 – 7.44 (m, 4H), 7.30 (t, J = 8.0 Hz, 1H), 7.17 – 7.12 (m, 4H), 6.95 – 6.88 (m, 2H), 6.41 (br s, 1H) ppm; ^{13}C{^{1}H}NMR (100 MHz, $CDCl_3$) δ 156.7, 144.3, 141.5, 140.3, 137.4, 132.9, 130.6, 129.4, 129.1, 128.5, 123.7, 122.0, 120.4, 115.3 ppm; IR (thin film): 2990, 1587, 1513, 1226, 1180, 1093, 989, 782, 615 cm^{-1}; HRMS calculated for $C_{18}H_{16}NO_2S$ 310.0896, found 310.0893 $[M+H]^+$.

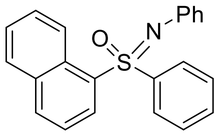

***N*-Phenyl-*S*-phenyl-*S*-(1-naphthalen)sulfoximine (3oa):** The reaction was performed following the General Procedure with **1o** (200.3 mg, 0.75 mmol) and **2a** (183.1 mg, 1.5 mmol). The crude product was purified by flash chromatography on silica gel (eluted with hexane:EtOAc = 5:1) to give the product **3oa** (180.1 mg, 70% yield) as a colorless solid. R_f = 0.3 (hexane:EtOAc = 5:1). m.p.= 130-131 °C; ^{1}H NMR (400 MHz, $CDCl_3$) *δ* 8.83 (d, *J* = 7.9 Hz, 1H), 8.65 (d, *J* = 6.7 Hz, 1H), 8.13 (m, 2H), 7.95 (d, *J* = 7.8 Hz, 1H), 7.76 (d, *J* = 7.2 Hz, 1H), 7.60 – 7.46 (m, 2H), 7.41 (m, 4H), 7.26 – 7.13 (m, 2H), 7.09 (d, *J* = 6.6 Hz, 2H), 6.82 (s, 1H) ppm; ^{13}C{^{1}H}NMR (100 MHz, $CDCl_3$) *δ* 144.8, 141.1, 135.1, 134.8, 134.5, 132.6, 131.7, 129.1, 129.0, 128.8, 128.2, 128.1, 126.7, 124.7, 124.5, 123.4, 121.8 ppm; IR (thin film): 3058, 1572, 1538, 1490, 1275, 1142, 1110, 962, 827, 658 cm^{-1}; HRMS calculated for $C_{22}H_{18}NOS$ 344.1104, found 344.1102 $[M+H]^+$.

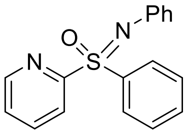

***N*-Phenyl-*S*-phenyl-*S*-(2-pyridinyl)sulfoximine (3pa):** The reaction was performed following the General Procedure with **1p** (163.5 mg, 0.75 mmol) and **2a** (183.1 mg, 1.5 mmol). The crude product was purified by flash chromatography on silica gel (eluted with hexane:EtOAc = 3:1) to give the product **3pa** (143.4 mg, 65% yield) as a colorless solid. R_f = 0.3 (hexane:EtOAc = 3:1). m.p.= 135-136 °C; ^{1}H NMR (600 MHz, $CDCl_3$) *δ* 8.62 (m, 1H), 8.37 (d, *J* = 7.9 Hz, 1H), 8.17 (d, *J* = 7.5 Hz, 2H), 7.90 – 7.82 (m, 1H), 7.57 – 7.52 (m, 1H), 7.49 (t, *J* = 7.5 Hz, 2H), 7.37 (d, *J* = 3.5 Hz, 1H), 7.18 – 7.10 (m, 4H), 6.89 (t, *J* = 7.0 Hz, 1H) ppm; ^{13}C{^{1}H}NMR (150 MHz, $CDCl_3$) *δ* 158.5, 150.4, 144.4, 138.4, 137.9, 133.2, 129.7, 129.1, 129.0, 126.3, 123.9, 123.7, 122.0 ppm; IR (thin film): 3228, 1572, 1496, 1354, 1371, 1276, 1245, 1179, 1054, 1018, 879, 757, 652 cm^{-1}; HRMS calculated for $C_{17}H_{15}N_2OS$ 295.0900, found 295.0896 $[M+H]^+$.

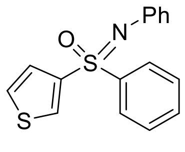

***N*-Phenyl-*S*,*S*-3-thiophenylsulfoximine (3qa):** The reaction was performed following the General Procedure with **1q** (176.3 mg, 0.75 mmol) and **2a** (183.1 mg, 1.5 mmol). The crude product was purified by flash chromatography on silica gel (eluted with hexane:EtOAc = 3:1) to give the product **3qa** (163.7 mg, 73% yield) as a colorless solid. R_f = 0.3 (hexane:EtOAc = 3:1). m.p. = 110-112 °C; ^{1}H NMR (400 MHz, $CDCl_3$) *δ* 8.17 (d, *J* = 7.9 Hz, 2H), 7.62 (t, *J* = 4.5 Hz, 1H), 7.61 – 7.47 (m, 4H), 7.25 – 7.15 (m, 4H), 7.06 – 6.99 (m, 1H), 6.99 – 6.91 (m, 1H) ppm; ^{13}C{^{1}H}NMR (100 MHz, $CDCl_3$) *δ* 144.3, 142.4, 141.5, 134.1, 133.9, 132.8, 129.3, 129.0, 128.2, 128.1, 123.9, 122.2 ppm; IR (thin film): 3440, 1585, 1483, 1275, 1209, 1090, 1030, 770, 739, 690, 617, 559 cm^{-1}; HRMS calculated for $C_{16}H_{14}NOS_2$ 300.0511, found

300.0508 $[M+H]^+$.

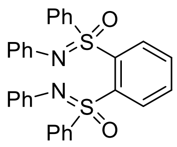

***N,N*-Diphenyl-*S,S*-1,2-diphenylsulfoximine (3ra):** The reaction was performed following the General Procedure with **1r** (267.0 mg, 0.75 mmol) and **2a** (183.1mg, 1.5 mmol), and stirred at room time under argon for 60 h. The crude product was purified by flash chromatography on silica gel (eluted with DCM:MeOH = 20:1) to give the product **3ra** (213.4 mg, 56% yield) as a single diastereoisomer as a brown solid. R_f = 0.3 (DCM:MeOH = 20:1). m.p. = 186-187 °C; ^{1}H NMR (400 MHz, $CDCl_3$) δ 8.13 (dd, J = 7.9, 1.4 Hz, 2H), 7.76 – 7.70 (m, 4H), 7.56 – 7.49 (m, 2H), 7.40 – 7.38 (m, 4H) 7.38 – 7.32 (m, 3H), 7.25 – 7.19 (m, 6H), 7.11 – 7.07 (m, 3H) ppm; ^{13}C{^{1}H}NMR (100 MHz, $CDCl_3$) δ 147.3, 145.1, 134.7, 133.9, 133.0 131.5, 131.2, 130.3, 129.4, 129.2, 129.1, 127.4, 126.3, 125.0 ppm; IR (thin film): 3368, 3054, 1602, 1476, 1440, 1335, 1046, 746, 685, 646 cm^{-1}; HRMS calculated for $C_{30}H_{25}N_2O_2S_2$ 509.1352, found 509.1354 $[M+H]^+$.

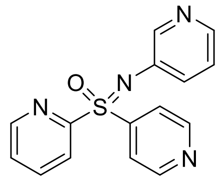

***N*-3-Pyridin-*S*-2-pyridin-*S*-(4-pyridin)sulfoximine (3sm):** The reaction was performed following the General Procedure with **1s** (65.7 mg, 0.3 mmol) and **2m** (84.9 mg, 0.69 mmol), $Cu(O_2CCF_3)_2{\cdot}H_2O$ (13.1 mg, 0.045 mmol, 15 mol %) in dry EtOH (1.0 mL), and stirred at room temperature under argon for 60 h. The crude product was purified by flash chromatography on silica gel (eluted with DCM:MeOH = 10:1) to give the product **3sm** (37.3 mg, 42% yield) as a colorless solid. R_f = 0.3 (DCM:MeOH = 10:1). ^{1}H NMR (400 MHz, CD_3OD) δ 8.82 (d, J = 5.9 Hz, 3H), 8.67 (s, 1H), 8.35 (d, J = 7.8 Hz, 1H), 8.15 – 8.08 (m, 2H), 8.07 – 8.02 (m, 3H), 7.67 – 7.60 (m, 2H) ppm; ^{13}C{^{1}H}NMR (100 MHz, CD_3OD) δ 158.5, 150.8, 150.3, 150.1, 138.8, 127.3, 122.7, 122.4, 121.7 ppm; IR (thin film): 3468, 3154, 1682, 1378, 1450, 1365, 1123, 846, 765, 624 cm^{-1}; HRMS calculated for $C_{15}H_{13}N_4OS$ 297.0805, found 297.0806 $[M+H]^+$.

Supplementary Discussion

Reaction in dark

Procedure: The reaction was performed following the General Procedure with **1a** (162.8 mg, 0.75 mmol, 1.0 equiv), boronic acid **2a** (183.1 mg, 1.5 mmol, 2.0 equiv), $Cu(O_2CCF_3){\cdot}H_2O$ (21.7 mg, 0.075 mmol, 0.1 equiv) under an argon atmosphere in dark. The product **3aa** was generated in only 8% yield (17.6 mg).

Catalytic reaction in the presence of TEMPO

Procedure: To an oven-dried microwave vial equipped with a stir bar was added sulfoximine **1a** (162.8 mg, 0.75 mmol, 1.0 equiv), boronic acid **2a** (183.1 mg, 1.5 mmol, 2.0 equiv), $Cu(O_2CCF_3)_2{\cdot}H_2O$ (21.7

mg, 0.075 mmol, 0.1 equiv), TEMPO (117 mg, 0.75 mmol, 1 equiv) under an argon atmosphere in a dry box. The vial was capped with a septum and removed from the dry box. EtOH (0.5 mL) was added into the reaction vial via syringe, and the reaction solution was stirred at room temperature under an argon atmosphere with ambient light for 48 h. Upon completion of the reaction, the vial was opened to air, and the reaction mixture was passed through a short pad of silica gel. The pad was then rinsed with 100:1 dichloromethane:methanol (20.0 mL). The solvent was removed under reduced pressure to afford a yellow oil. The resulting oil was concentrated and then purified by flash chromatography on silica gel (eluted with hexane:EA = 5:1) to afford product **4** as a yellow solid (11.7 mg, 10% yield). R_f = 0.3 (hexane:EA = 5:1). The spectroscopic data match the previously reported data.[5]

Spin Trap with DMPO[5-6]

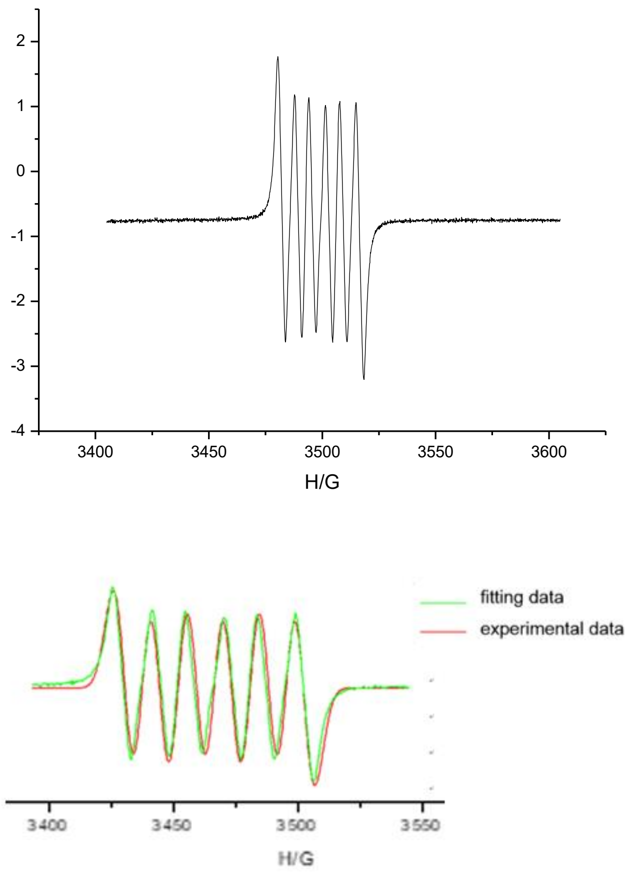

Supplementary Fig. 1| EPR Spectrum of the Reaction Mixture in the presence of DMPO: Experimental Data and Fitting Data.

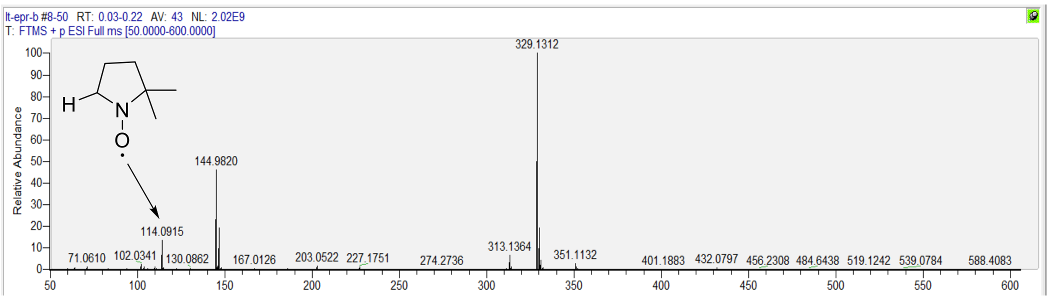

Supplementary Fig. 2| HRMS spectra of **5**.

Procedure: To an oven-dried microwave vial equipped with a stir bar was added sulfoximine **1a** (162.8 mg, 0.75 mmol, 1.0 equiv), boronic acid **2a** (183.1 mg, 1.5 mmol, 2.0 equiv) and $Cu(O_2CCF_3)\cdot H_2O$ (21.7 mg, 0.075 mmol, 0.1 equiv) under an argon atmosphere in a dry box. The vial was capped with a septum and removed from the dry box. EtOH (0.5 mL) was added into the reaction vial via syringe, and the reaction solution was stirred at room temperature under an argon atmosphere with ambient light for 24 h. Next, DMPO (5,5-dimethyl-1-pyrroline N-oxide, 11.3 mg, 0.10 mmol) was added under argon. The reaction mixture was analyzed by electron paramagnetic resonance (EPR) immediately at room temperature. We did data fitting. And HRMS analysis of the reaction mixture confirmed formation of the H• trapping product. EPR spectrometer operated at 9.8243 GHz. Typical spectrometer parameters are shown as follows, scan range: 200 G; center field set: 3505.00 G; scan time: 60 s; modulation amplitude: 1.0 G; modulation frequency: 100 kHz; receiver gain: 30 db; microwave power: 2.00 mW. The fitting data are A_N = 13.60 G, A_H = 6.62 G. HRMS calculated for $C_6H_{12}NO$: 114.0919, found 114.0915 $[M]^+$.

Headspace-Gas Chromatography (GC)

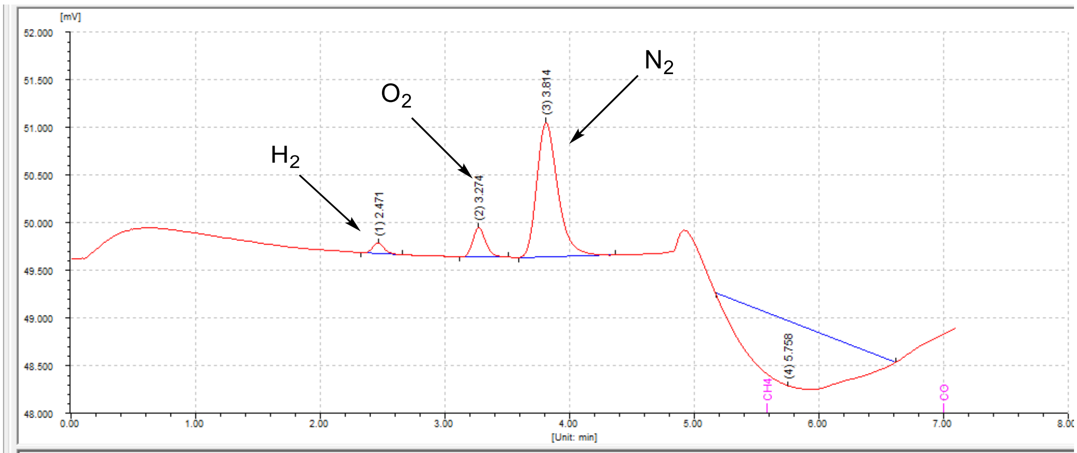

峰序	组分名	保留时间[min]	半峰宽[min]	峰高[uV]	峰面积[uV*s]	峰面积[%]	含量[mg/mL]	峰类型
1		2.471	0.105	113.9	784.4	0.0000	0.0000	BB
2		3.274	0.112	311.4	2291.7	0.0000	0.0000	BB
3		3.814	0.176	1411.9	16665.6	0.0000	0.0000	BB
4	CH4	5.758	1.187	679.1	37473.0	0.0000	0.0000	BB
			总计:	2516.2	57214.6	0.0000	0.0000	

Supplementary Fig. 3| GC spectrum of the headspace gas above the liquid surface in the sealed vial.

Procedure: To an oven-dried microwave vial equipped with a stir bar was added sulfoximine **1a** (325.5 mg, 1.5 mmol, 1.0 equiv), boronic acid **2a** (366.2 mg, 3.0 mmol, 2.0 equiv), $Cu(O_2CCF_3)^{\cdot}H_2O$ (43.4 mg, 0.15 mmol, 0.1 equiv) under an argon atmosphere in a dry box. The vial was capped with a septum and removed from the dry box. EtOH (0.5 mL) was added into the reaction vial via syringe, and the reaction solution was stirred at room temperature under an argon atmosphere with ambient light for 24 h. Then the gas above the liquid surface was extracted by the GC sampling needle for detection. GC spectra were recorded at room temperature on a Fuli GC 9790 plus. The GC spectrum clearly showed the existence of H_2 generated during the reaction.

Light on/off Experiment

Five parallel reactions were performed between sulfoximine **1a** (325.5 mg, 1.5 mmol, 1.0 equiv), phenylboronic acid **2a** (365.8 mg, 3.0 mmol, 2 equiv) and $Cu(O_2CCF_3)_2{}^{\cdot}H_2O$ (43.4 mg, 0.15 mmol, 10 mol %) in EtOH (1.0 mL, 1.5 M) according to the General Procedure. The assay yield (AY) was determined by ^{1}H NMR with 0.1 mmol CH_2Br_2 (7.0 μL) as internal standard at the given times. The white area indicates the light irradiation, while the grey area indicates time in the dark (Fig. 2d).

Reaction profile

Standard conditions: 21 parallel reactions were performed between sulfoximine **1a** (65.1 mg, 0.3 mmol, 1.0 equiv), phenylboronic acid **2a** (73.2 mg, 0.6 mmol, 2.0 equiv), and $Cu(O_2CCF_3)_2{}^{\cdot}H_2O$ (8.7 mg, 0.03 mmol, 10 mol %) in EtOH (0.2 mL, 1.5 M) according to the General Procedure. The reactions were all started at the same time. Upon specified reaction time, the separate vial was opened to air, and the reaction mixture was passed through a short pad of silica gel. The pad was then rinsed with 100:1 DCM:MeOH (20.0 mL). The solvent was removed under reduced pressure. The assay yield (AY) was determined by ^{1}H NMR with 0.1 mmol CH_2Br_2 (7.0 μL) as internal standard (Fig. 3a).

Initiation in the Presence of 3aa

With an initial ratio of **1a**/**3aa**=4/1: nine parallel reactions were performed between sulfoximine **1a** (34.7 mg, 0.15 mmol), *N*-Ph sulfoximine **3aa** (11.0 mg, 0.038 mmol), phenylboronic acid **2a** (73.2 mg, 0.30 mmol, 2.0 equiv of **1a**), and $Cu(O_2CCF_3)_2{}^{\cdot}H_2O$ (5.8 mg, 0.02 mmol, 10 mol %) in EtOH (0.2 mL, 1.0 M) according to the General Procedure. All reactions were started at the same time. Upon specified reaction time, the separate vial was opened to air, and the reaction mixture was passed through a short pad of silica gel. The pad was then rinsed with 100:1 DCM:MeOH (20.0 mL). The solvent was removed under reduced pressure. The assay yield (AY) was determined by ^{1}H NMR with 0.1 mmol CH_2Br_2 (7.0 μL) as internal standard (Fig. 3b).

When initial ratio of **1a**/**3aa**=2/1: six parallel reactions were performed between sulfoximine **1a** (28.9 mg, 0.13 mmol), *N*-Ph sulfoximine **3aa** (19.5 mg, 0.07 mmol), phenylboronic acid **2a** (31.7 mg, 0.26 mmol, 2.0 equiv of **1a**), and $Cu(O_2CCF_3)_2 \cdot H_2O$ (5.8 mg, 0.02 mmol, 10 mol %) in EtOH (0.4 mL, 0.5 M) according to General Procedure, starting at the same time. Upon specified reaction time, the separate vial was opened to air, and the reaction mixture was passed through a short pad of silica gel. The pad was then rinsed with 100:1 DCM:MeOH (20.0 mL). The solvent was removed under reduced pressure. The assay yield (AY) was determined by ^1H NMR with 0.1 mmol CH_2Br_2 (7.0 μL) as internal standard (Fig. 3b).

HRMS data of different reaction stages

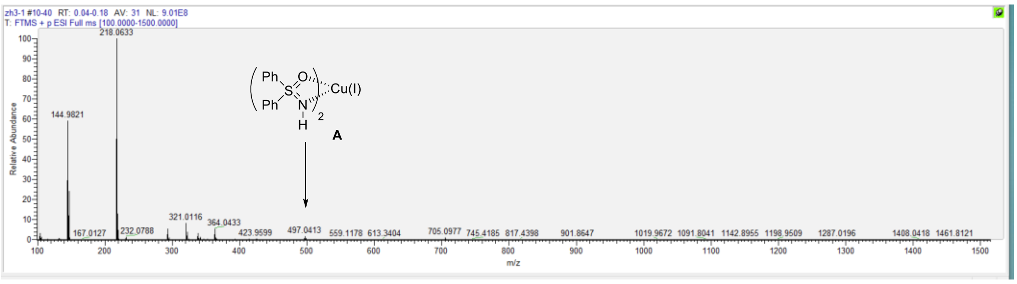

a. Reaction time: 5 h

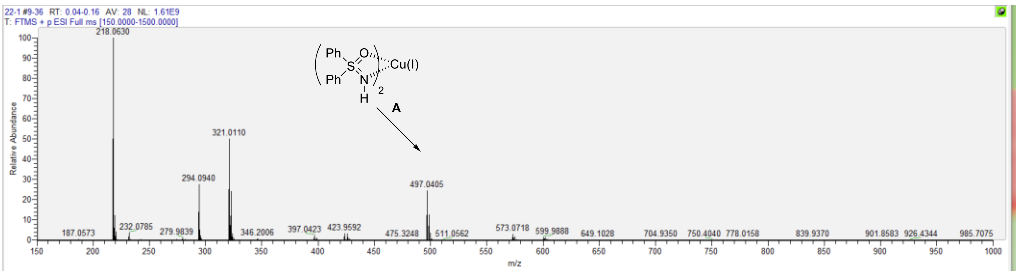

b. Reaction time: 10 h

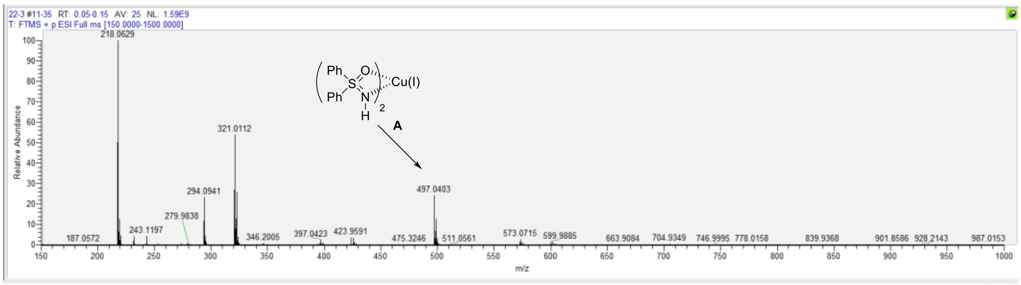

c. Reaction time: 20 h

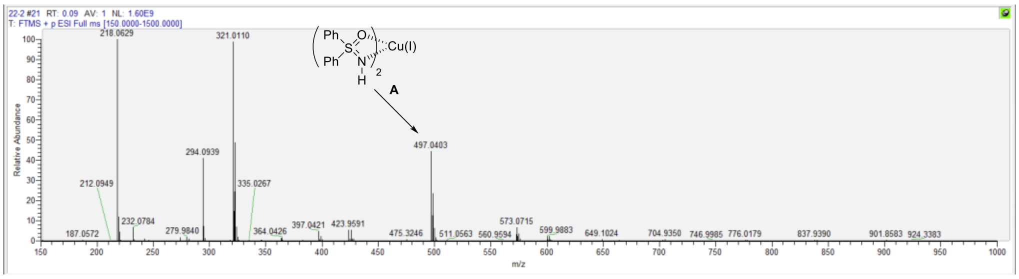

d. Reaction time: 25 h

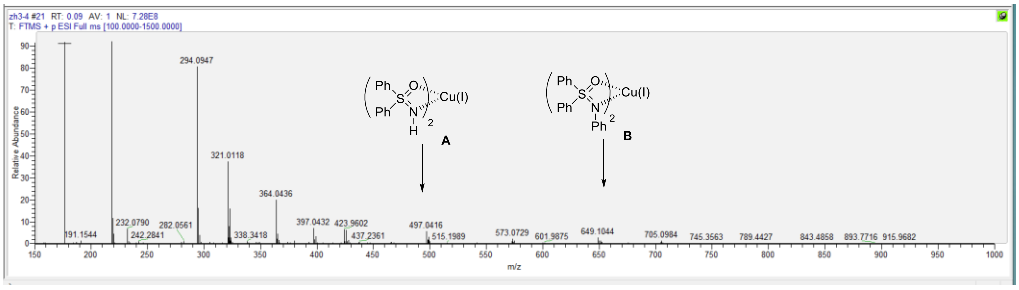

e. Reaction time: 30 h

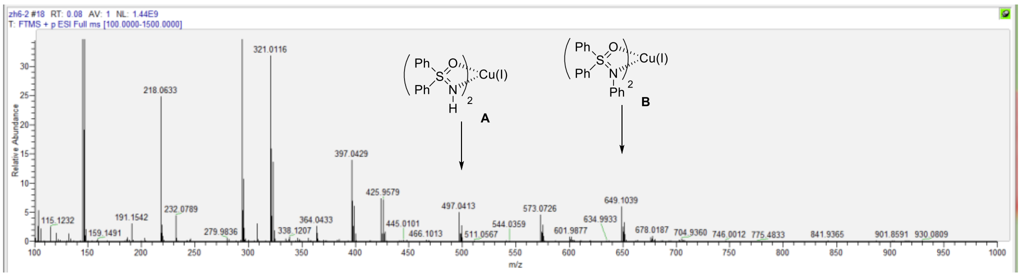

f. Reaction time: 36 h

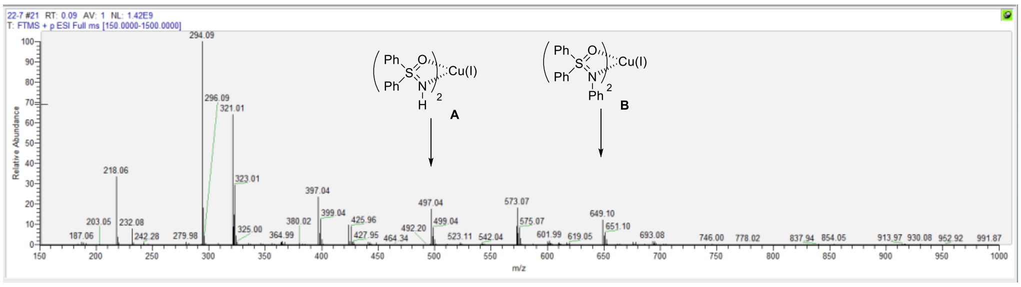

g. Reaction time: 42 h

Supplementary Fig. 4| HRMS spectra of copper-catalyzed photoredox Chan-Lam coupling reaction of **1a** and **2a** at different reaction times.

Copper-Catalyzed Photoredox Chan-Lam Coupling Reaction of Phenyl Methyl Sulfoximine

Supplementary Table 1| Copper-Catalyzed Photoredox Chan-Lam Coupling Reactions of Methyl Phenyl Sulfoximine with **2a**.

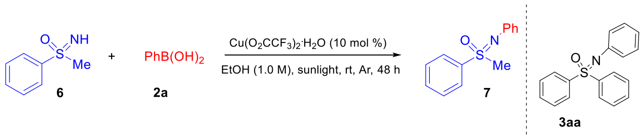

Entry	additive (**3aa**)/mol%	isolated yield/%
1	20	80
2	0	7

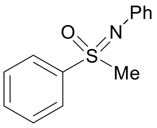

Procedure: To an oven-dried microwave vial equipped with a stir bar was added sulfoximine **6** (31.0 mg, 0.2 mmol, 1.0 equiv), phenyl boronic acid **2a** (48.4 mg, 0.4 mmol, 2.0 equiv), $Cu(O_2CCF_3)_2{\cdot}H_2O$ (5.8 mg, 0.02 mmol, 10 mol %), and **3aa** (11.7

mg, 20 mol %) under an argon atmosphere in a dry box. The vial was capped with a septum and removed from the dry box. EtOH (0.2 mL) was added into the reaction vial via syringe and the reaction solution was stirred at room temperature under argon with ambient light for 48 h. Upon completion of the reaction, the vial was opened to air, and the reaction mixture was passed through a short pad of silica gel. The pad was then rinsed with 100:1 dichloromethane:methanol (20.0 mL). The solvent was removed under reduced pressure to afford a yellow oil. The crude product was purified by flash chromatography on silica gel (eluted with PE:EA = 2:1) to give the product **7** (37.0 mg, 80% yield) as a colorless solid. R_f = 0.3 (PE:EA = 1:1). ^{1}H NMR (400 MHz, $CDCl_3$) δ 8.02 – 7.93 (m, 2H), 7.62 – 7.55 (m, 1H), 7.56 – 7.48 (m, 2H), 7.16 – 7.09 (m, 2H), 7.05 – 6.97 (m, 1H), 6.83 – 6.87 (m, 2H), 3.24 (s, 3H) ppm; ^{13}C{^{1}H}NMR (100 MHz, $CDCl_3$) δ 144.9, 139.4, 133.3, 129.6, 129.0, 128.7, 123.3, 121.8, 46.0 ppm. The spectroscopic data match the previously reported data[7].

When the reaction was conducted without addition of **3aa** the product **7** was obtained (3.2 mg, 7% yield).

Preparation of Complex A' and B'

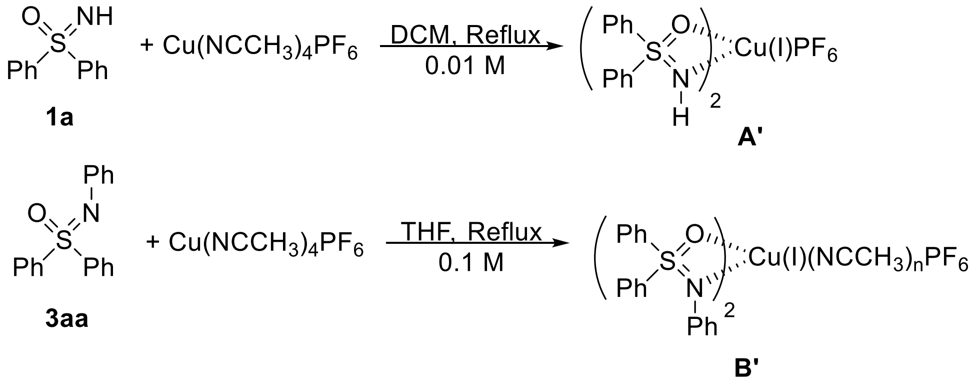

Fig S5| Preparation of copper(I) complex **A'** and **B'**.

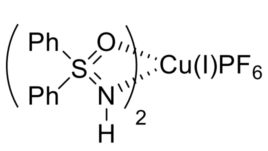

To an oven-dried tube equipped with a stir bar was added sulfoximine **1a** (21.7 mg, 0.1 mmol) and $Cu(NCCH_3)_4PF_6$ (18.6 mg, 0.05 mmol) and DCM (10.0 mL, 0.01 M) in a dry box. The mixture was then heated to reflux for 6 h. After the mixture was cooled down to room temperature, hexane (10.0 mL) was added to the mixture. The resulting solution was filtrated to afford **A'** as a white solid, m.p.= 236-238 °C; ^{1}H NMR (600 MHz, $CDCl_3$) δ 8.06 (d, J = 8.0 Hz, 8H), 7.66 (t, J = 7.4 Hz, 4H), 7.62 – 7.54 (m, 8H) ppm; ^{13}C{^{1}H}NMR (150 MHz, $CDCl_3$) δ 134.7, 130.1, 128.2 ppm; IR (thin film): 3221, 1582, 1476, 1448, 1409, 1310, 1211, 1158, 1093, 1071, 1028, 1008, 983, 880, 847, 834, 781, 769, 757, 725, 684, 623 cm^{-1}; HRMS calculated for $C_{24}H_{22}CuN_2O_2S_2^+$ 497.0413, found 497.0413 $[M- PF_6^-]^+$.

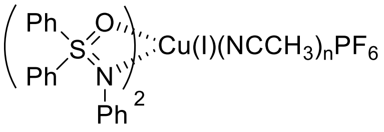

To an oven-dried tube equipped with a stir bar was added sulfoximine **3aa** (29.3 mg, 0.1 mmol) and $Cu(NCCH_3)_4PF_6$ (18.6 mg, 0.05 mmol) and THF (1.0 mL, 0.1 M) in a dry box. The mixture was then heated to reflux for 6 h. After the mixture was cooled down to room temperature, hexane (5.0 mL) was added to the mixture. The resulting solution was filtrated to afford **B'** as a white solid, m.p.= 147-149 °C; ^{1}H NMR (400 MHz, $CDCl_3$) δ 8.22 – 7.94 (m, 8H), 7.58 – 7.45 (m, 12H), 7.22 – 7.16 (m, 8H), 7.11 (t, J = 7.8 Hz, 2H), 6.97 (t, J = 7.3 Hz, 2H), 2.10 (s, 12 H) ppm; $^{13}C\{^1H\}$NMR (150 MHz, $CDCl_3$) δ142.9, 138.5, 133.7, 129.8, 129.2, 128.8, 125.0, 123.6, 116.5, 2.1 ppm; IR (thin film): 1592, 1681, 1485, 1447, 1283, 1263, 1205, 1172, 1086, 1071, 1034, 1011, 987, 897, 880, 833, 786, 751, 725, 687, 652, 622; HRMS calculated for $C_{36}H_{30}CuN_2O_2S_2^+$ 649.1039, found 649.1039 $[M-(CH_3CN)_n-PF_6^-]^+$.

UV/Vis-Absorption Spectra of the Reaction Components[8]

Preparation of the samples for UV-Vis spectra measurement. *(All the samples were freshly prepared for UV-Vis spectra measurement.)*

1a, **2a**, **3aa**, **A'**, **B'** and $Cu(NCCH_3)_4PF_6$: 5×10^{-5} M solutions (in DCM) were prepared in a dry box. Fresh measurement of solution in colorimetric vessel by UV.

Reaction solution (0.5 M in EtOH)**:** To an oven-dried microwave vial equipped with a stir bar was added sulfoximine **1a** (325.5 mg, 1.5 mmol), phenylboronic acid **2a** (421.0 mg, 3.5 mmol) and $Cu(O_2CCF_3)_2{\cdot}H_2O$ (43.3 mg, 0.15 mmol) and EtOH (3.0 mL) under an argon atmosphere in a dry box at room temperature for 24 h. The reaction solution was transferred into a colorimetric cell by syringe, sealed with a cap and removed from the dry box. Fresh measurement of solution in colorimetric vessel by UV.

Emission Quenching Experiments

Solutions of **A'** and **B'** were excited at 344 nm and the emission intensity at 379 and 399 nm were observed. In the typical experiment, 5×10^{-5} M **A'**, **B'** and $Cu(NCCH_3)_4PF_6$ solutions (in DCM) were prepared in a dry box. The separate emission spectra of the above solutions were collected. Then, appropriate amount of quencher was added to the measured solution and the emission spectrum of the sample was collected.

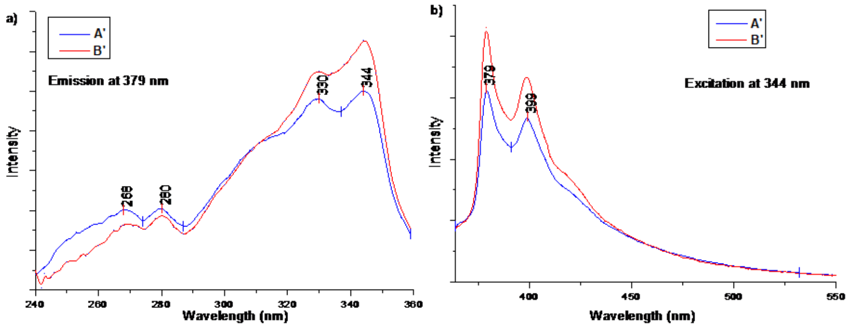

Supplementary Fig. 6| a) Excitation spectrum of **A’** and **B’** in DCM; b) Emission spectrum of **A’** and **B’** in DCM.

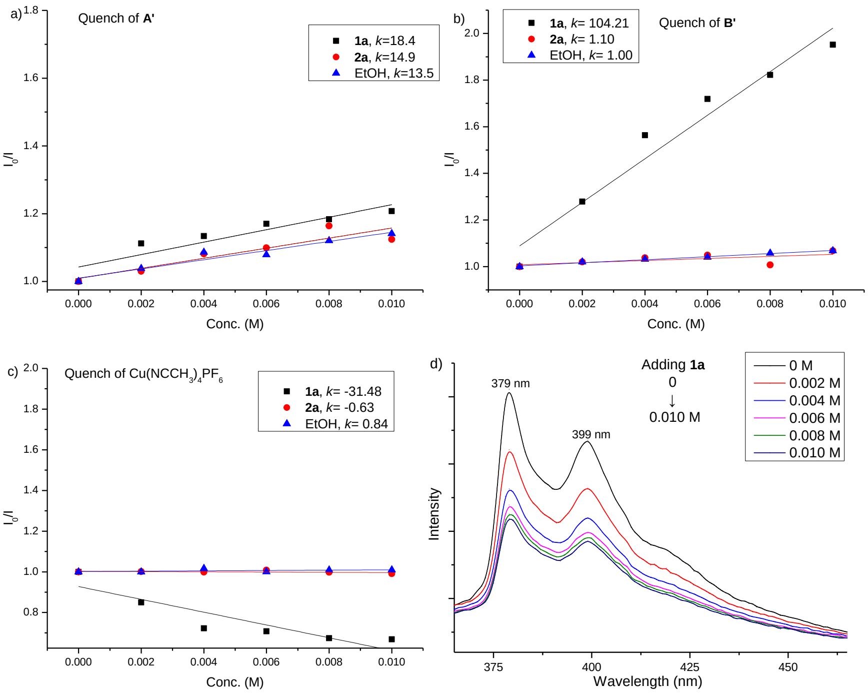

Supplementary Fig. 7| a) Stern-Volmer Quench of **A’** with **1a**, **2a** and EtOH; b) Stern-Volmer Quench of **B’** with **1a**, **2a** and EtOH; c) Stern-Volmer Quench of $Cu(NCCH_3)_4PF_6$ with **1a**, **2a** and EtOH; d) Emission quench of **B’** with **1a**.

Computational Studies

Optimizations of the transition states, starting materials, products, and intermediates were performed using Gaussian 16[9] software with unrestricted DFT using UB3LYP[10, 11, 12] functional and split basis set (6-31G(d) for C, S, O, H, N and SDD for Cu) in the gas phase. For all species, the correct multiplicities from the unrestricted calculations were confirmed. Vibrational frequencies were also computed to obtain

thermal Gibbs free energy corrections (at 298 K) and to characterize the stationary points as transition states (one and only one imaginary frequency) or minima (zero imaginary frequencies). For each frequency job, it was confirmed that convergence fully occurred. Transition states were confirmed by intrinsic reaction coordinate analysis. Conformational analysis was performed manually. Graphics were made in Cylview.[13]

The thermodynamic cycle was calculated to compare the energies of the copper spcies (Supplementary Fig. 8). The copper (II) species are ~70 kcal/mol higher in energy than the copper (I) species.

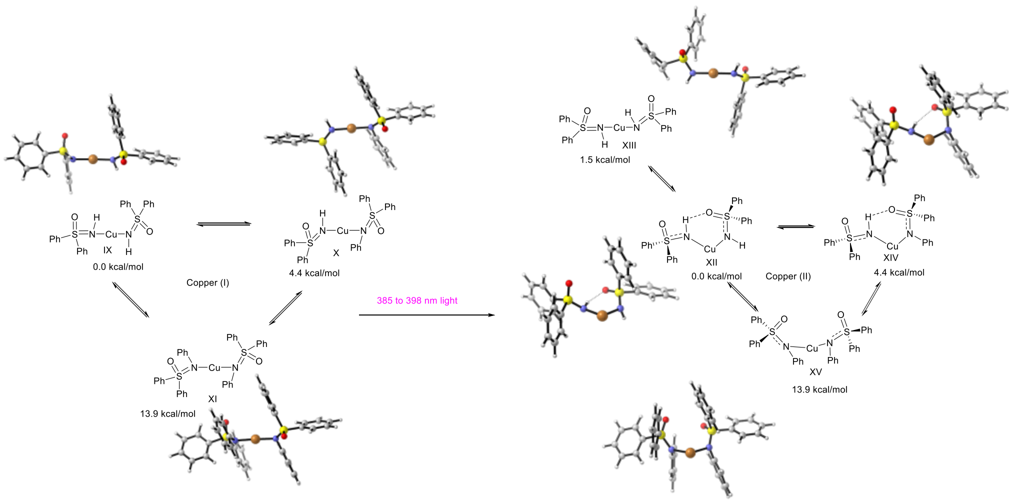

Supplementary Fig. 8| Thermodynamic cycles of substrate/product ligand exchange for Cu(I) and Cu(II).

The formation of copper hydride (Supplementary Fig. 9) has a barrier of 27.6 kcal/mol.

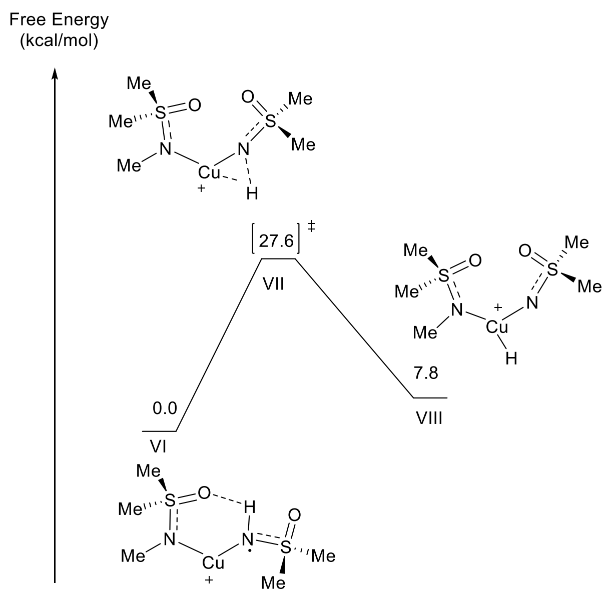

Supplementary Fig. 9| Pathway for copper hydride formation. Free energies computed using B3LYP/6-31G(d), Cu:SDD.

Supplementary Notes

NMR Spectra

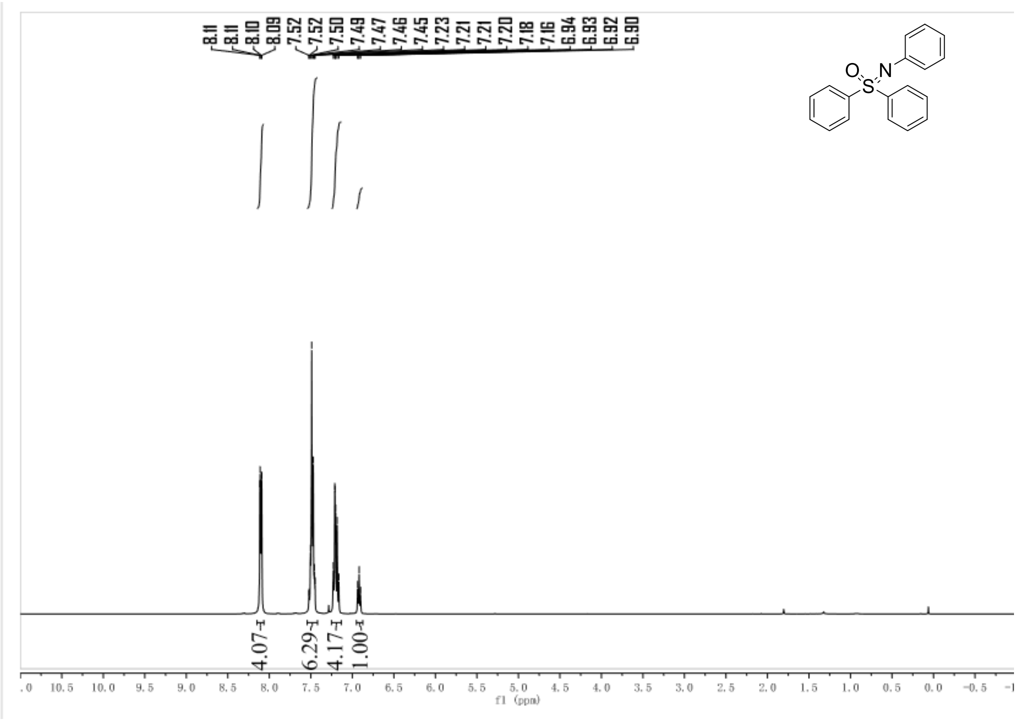

Supplementary Figure 10. ^{1}H NMR spectra (CDCl$_3$, 400 MHz) of compound 3aa

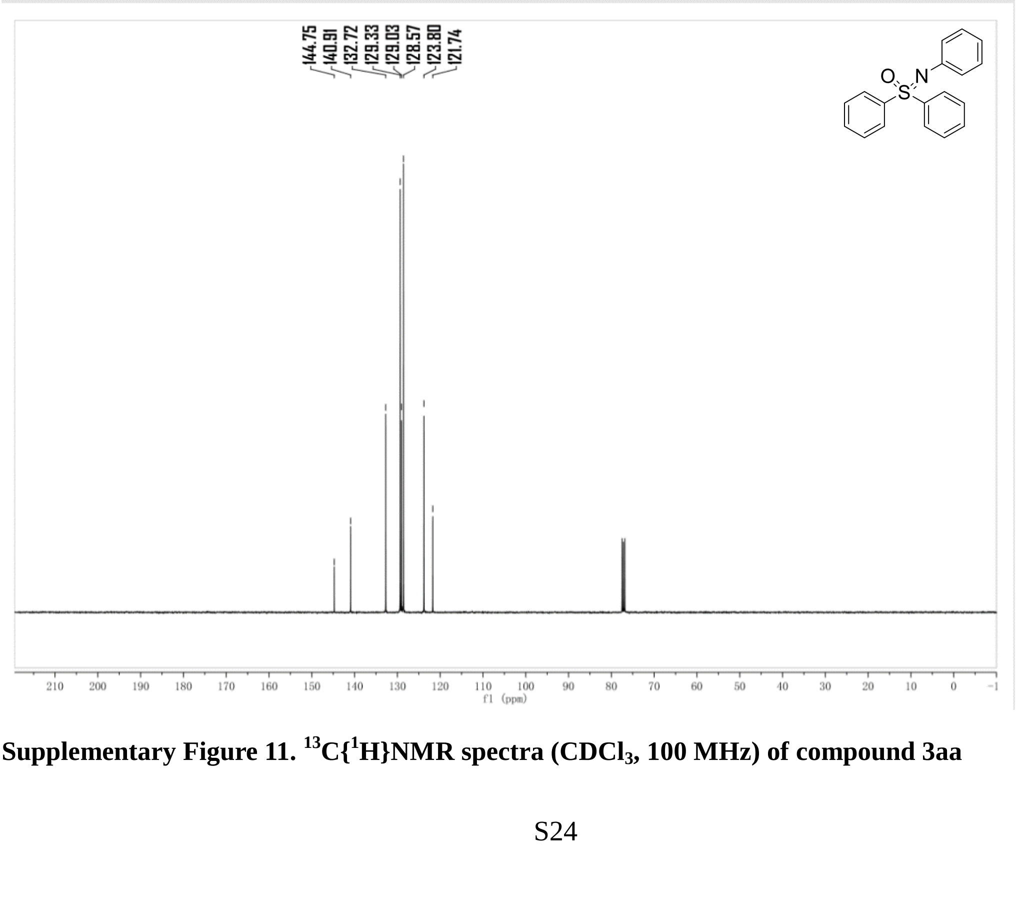

Supplementary Figure 11. ^{13}C{^{1}H}NMR spectra (CDCl$_3$, 100 MHz) of compound 3aa

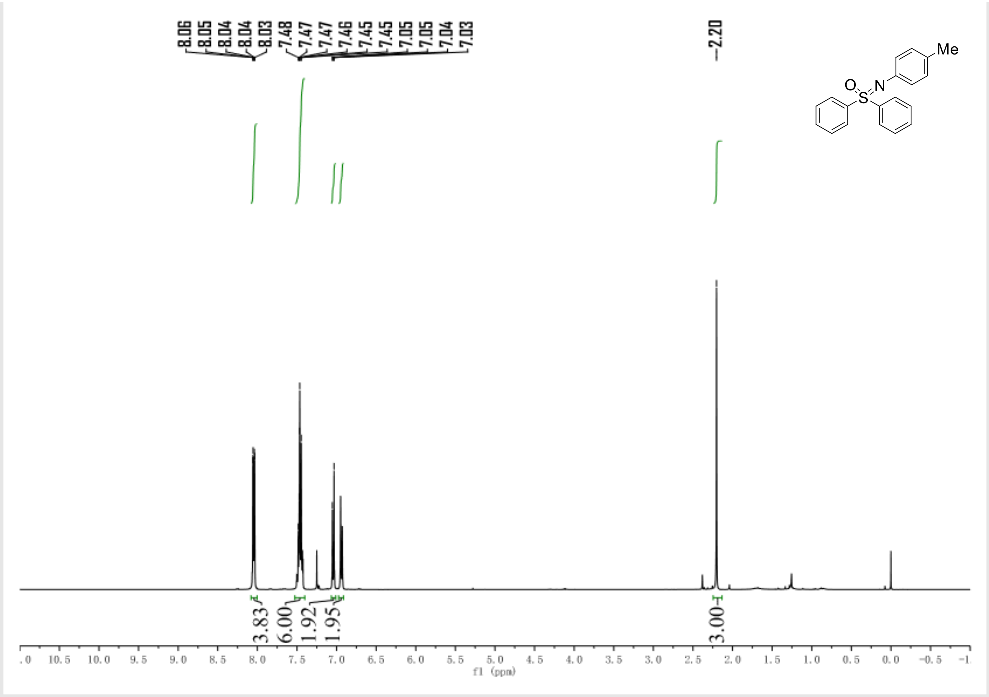

Supplementary Figure 12. ^{1}**H NMR spectra (CDCl$_3$, 400 MHz) of compound 3ab**

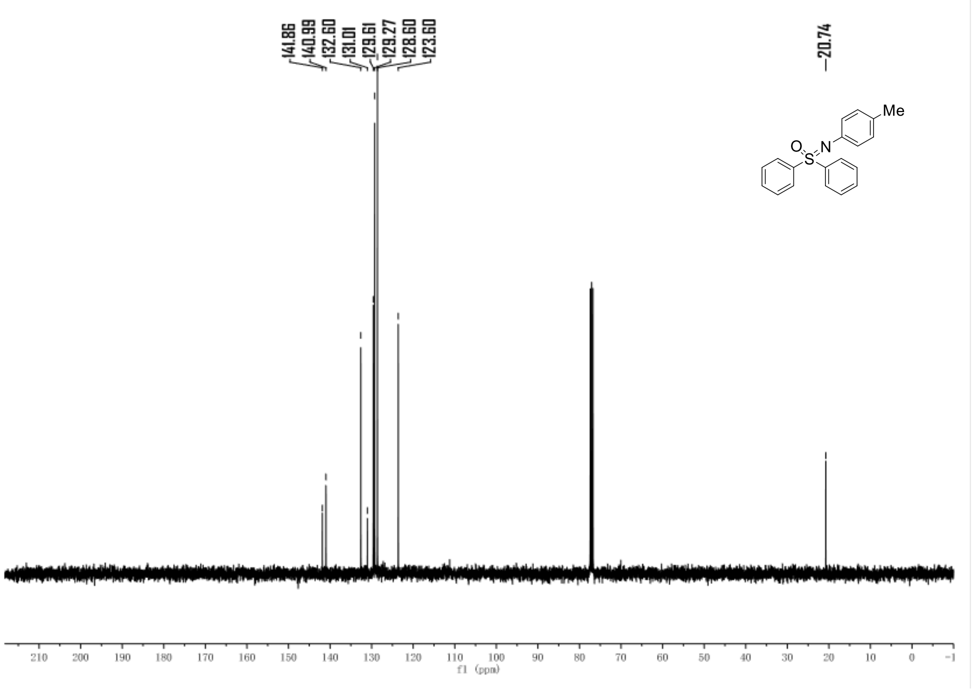

Supplementary Figure 13. ^{13}**C{^{1}H}NMR spectra (CDCl$_3$, 100 MHz) of compound 3ab**

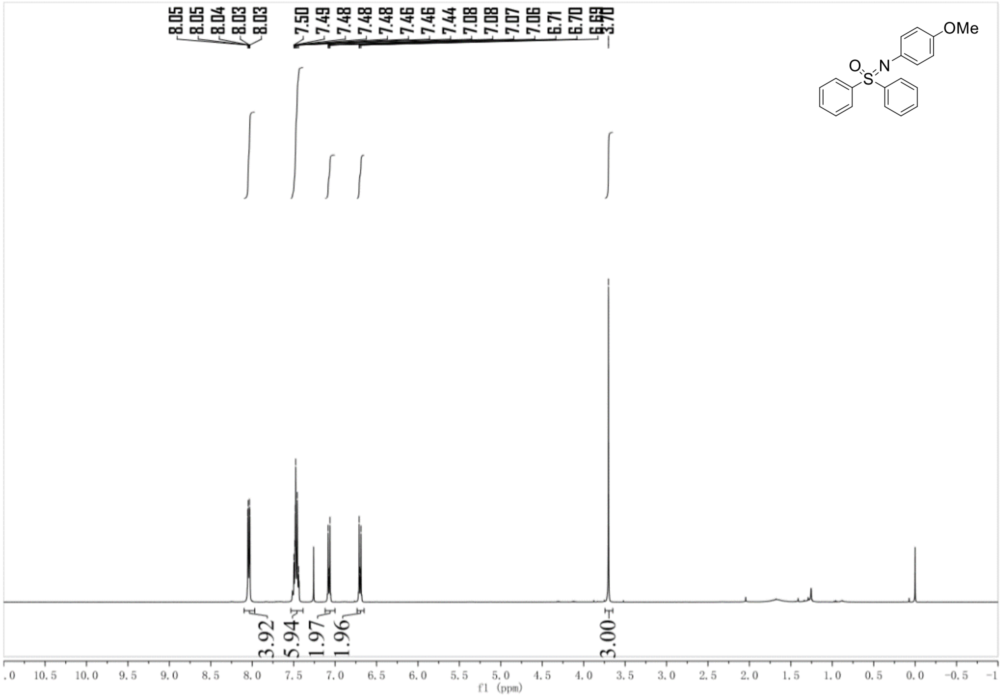

Supplementary Figure 14. **^{1}H NMR spectra ($CDCl_3$, 400 MHz) of compound 3ac**

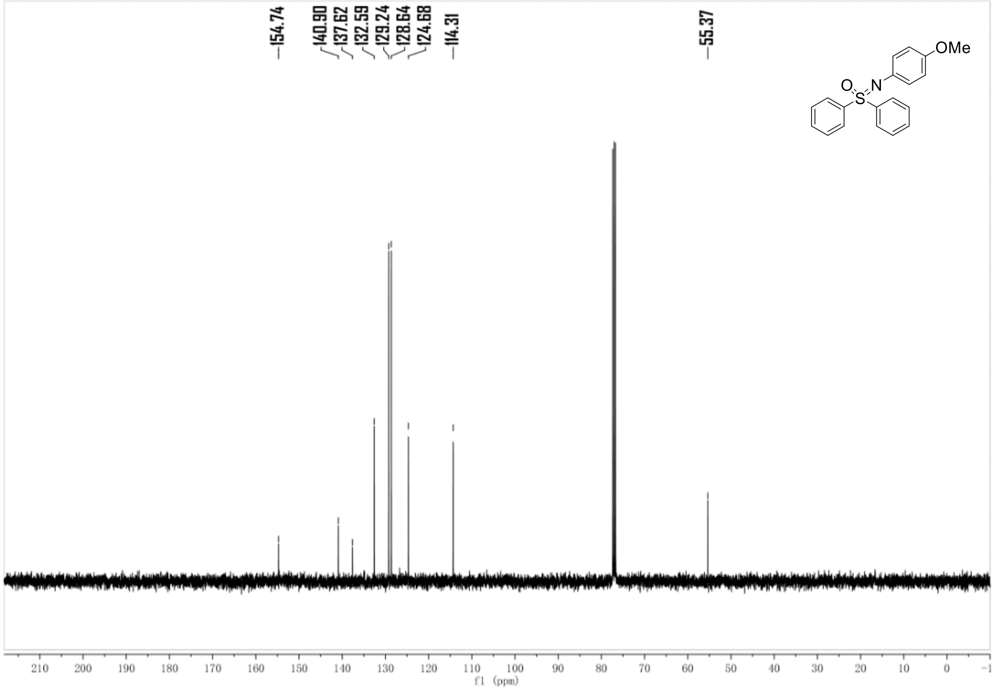

Supplementary Figure 15. **^{13}C{^{1}H}NMR spectra ($CDCl_3$, 100 MHz) of compound 3ac**

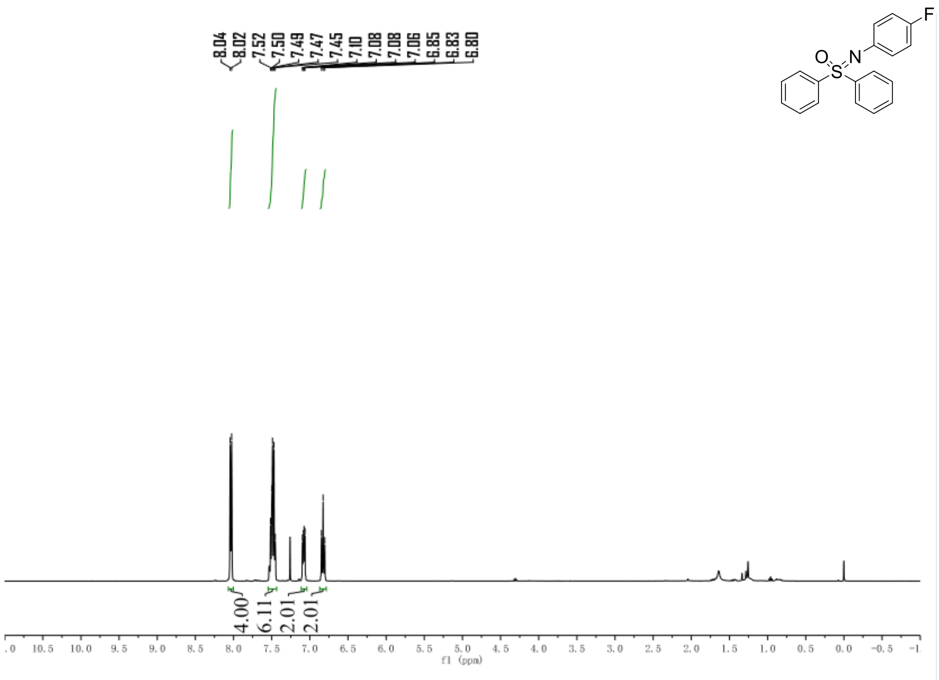

Supplementary Figure 16. ^{1}H NMR spectra ($CDCl_3$, 400 MHz) of compound 3ad

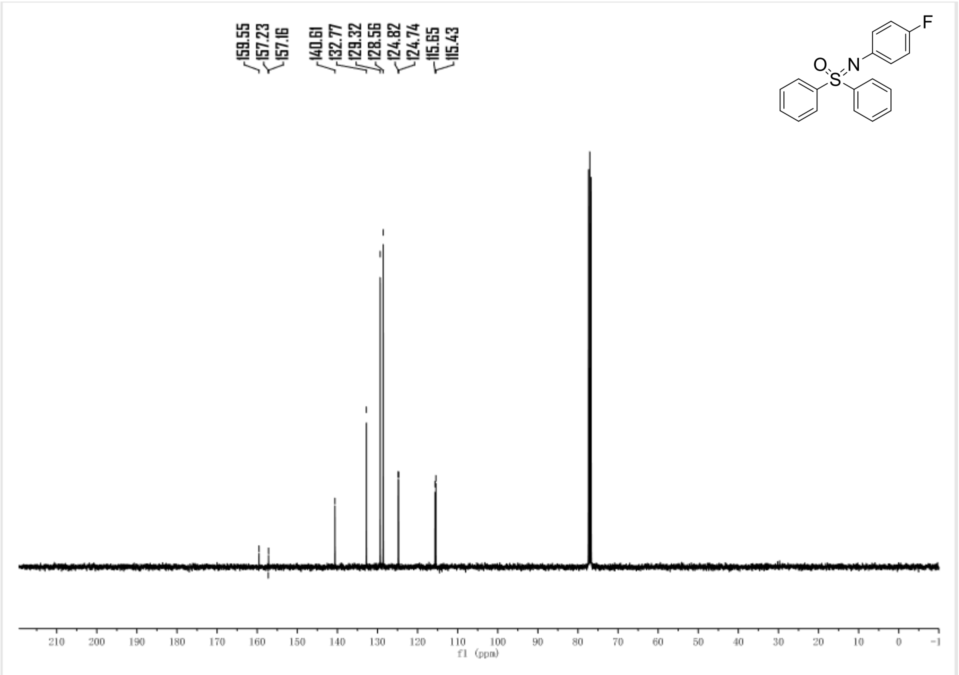

Supplementary Figure 17. $^{13}C\{^1H\}$NMR spectra ($CDCl_3$, 100 MHz) of compound 3ad

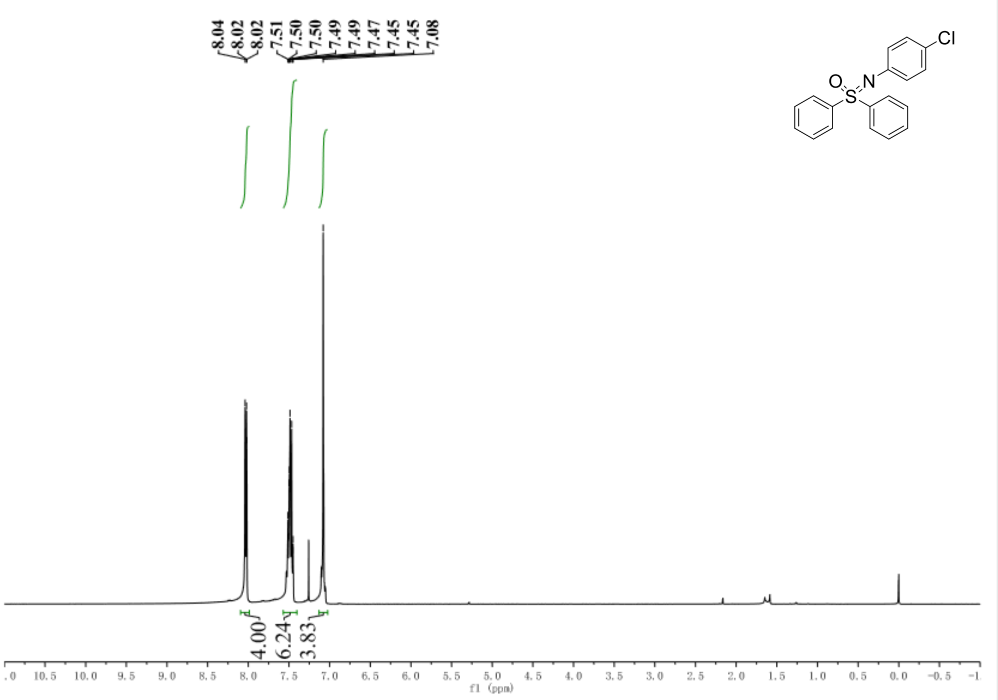

Supplementary Figure 18. ^{1}**H NMR spectra (CDCl$_3$, 400 MHz) of compound 3ae**

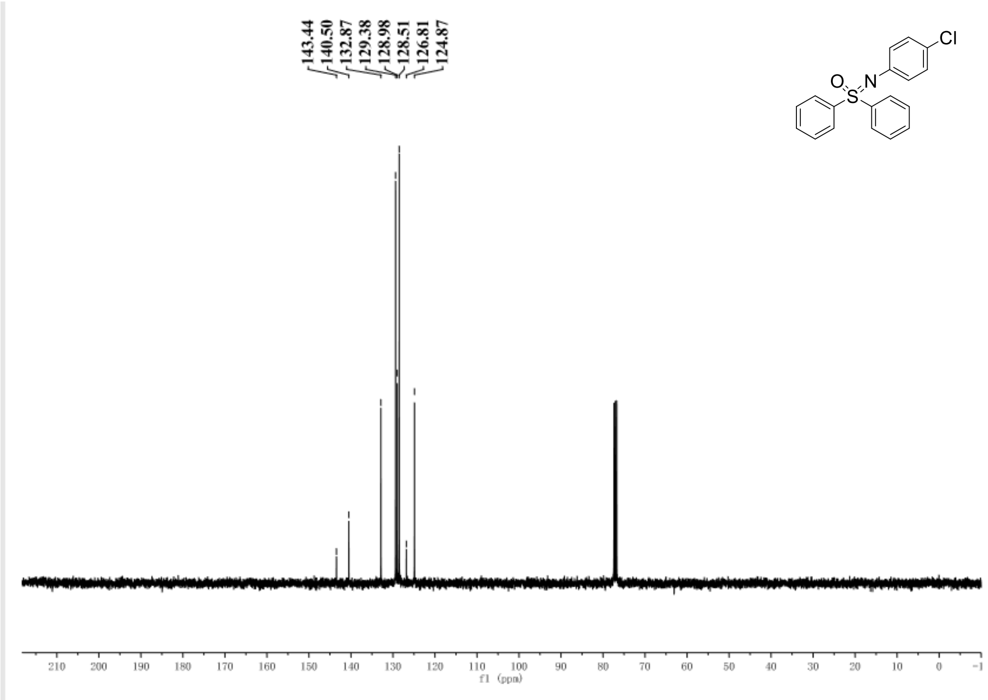

Supplementary Figure 19. ^{13}**C{^{1}H}NMR spectra (CDCl$_3$, 100 MHz) of compound 3ae**

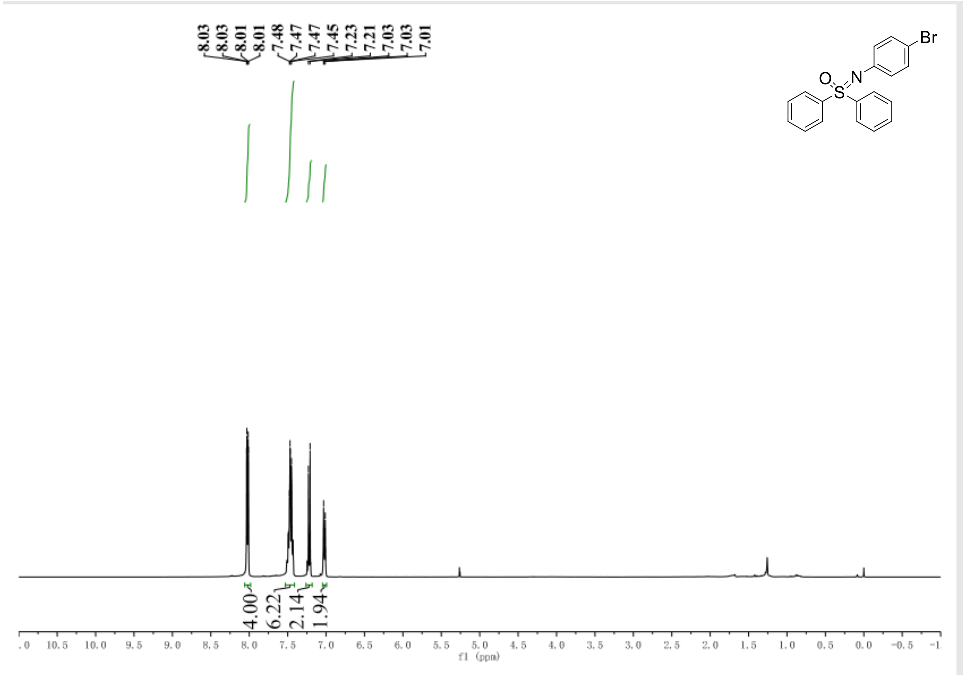

Supplementary Figure 20. ^{1}H NMR spectra ($CDCl_3$, 400 MHz) of compound 3af

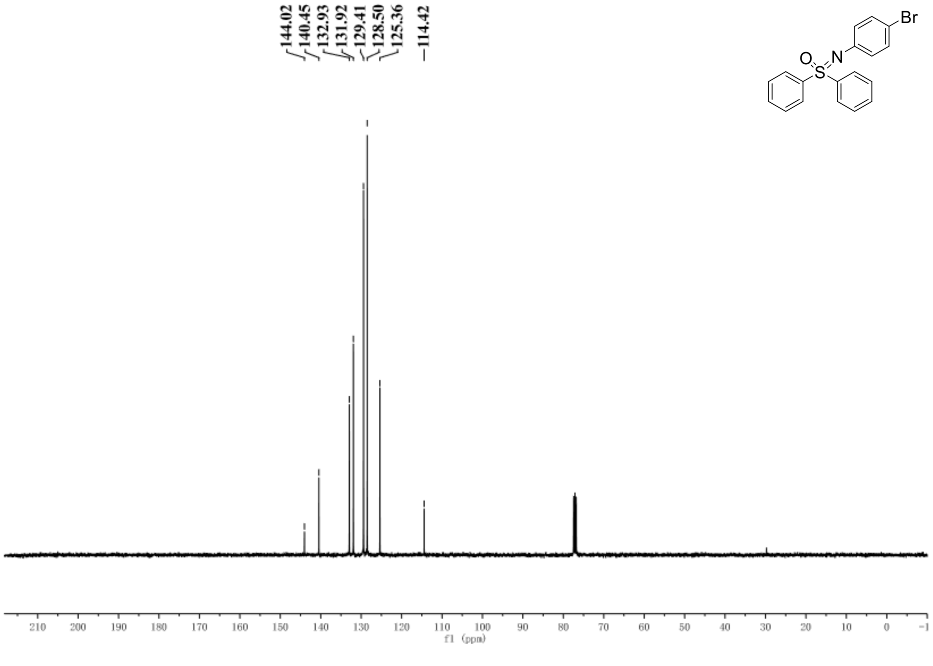

Supplementary Figure 21. $^{13}C\{^1H\}$NMR spectra ($CDCl_3$, 100 MHz) of compound 3af

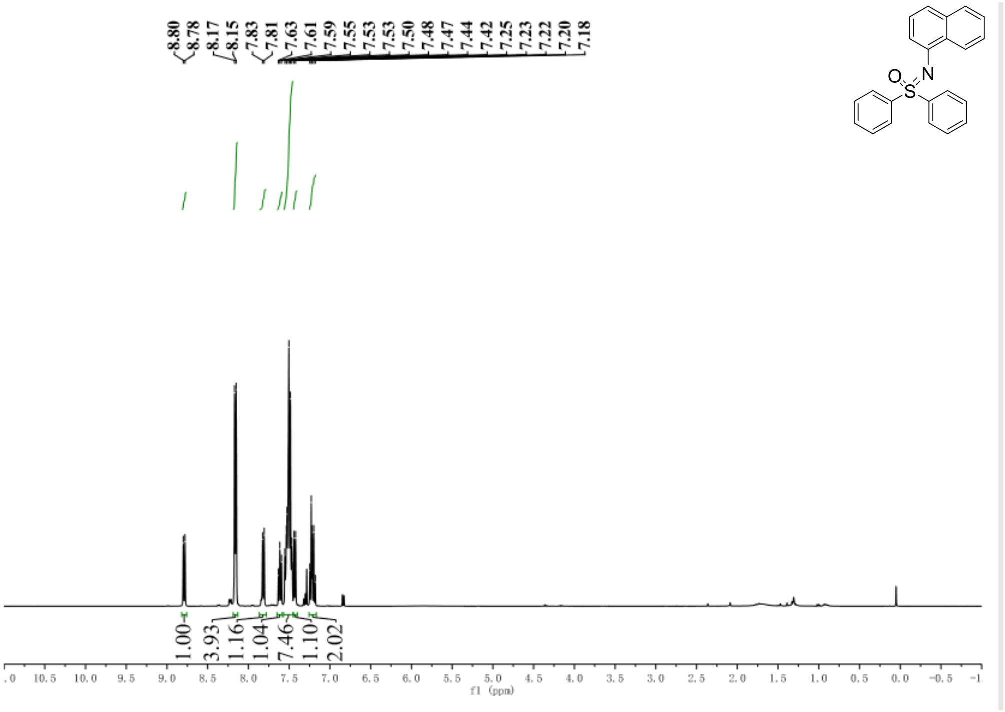

Supplementary Figure 22. ^{1}H NMR spectra ($CDCl_3$, 400 MHz) of compound 3ag

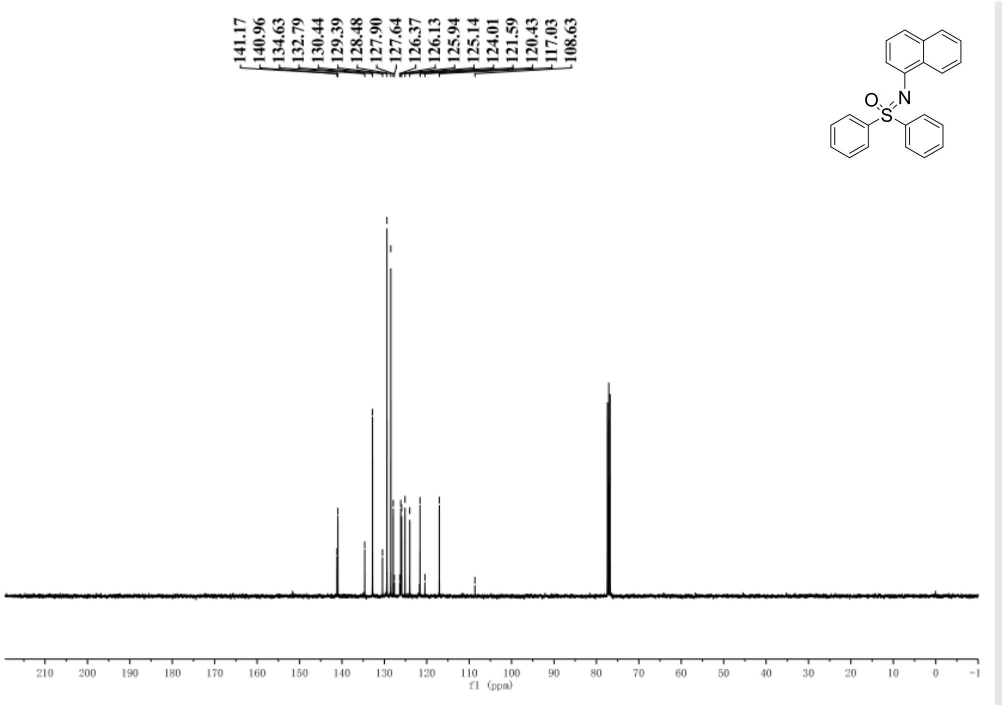

Supplementary Figure 23. $^{13}C\{^1H\}$NMR spectra ($CDCl_3$, 100 MHz) of compound 3ag

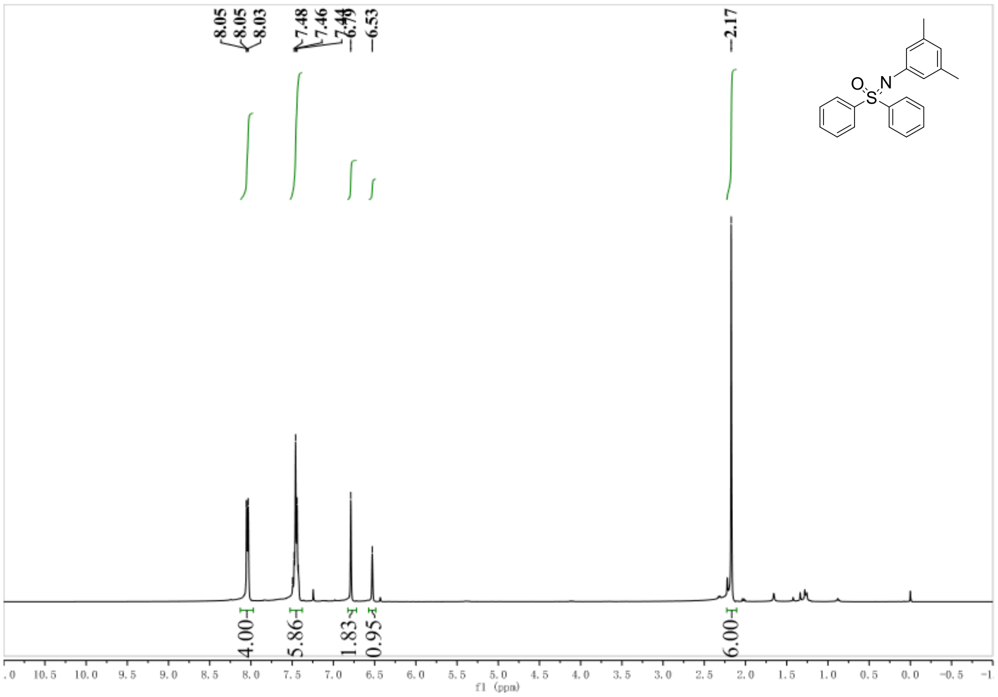

Supplementary Figure 24. ^{1}H NMR spectra ($CDCl_3$, 400 MHz) of compound 3ah

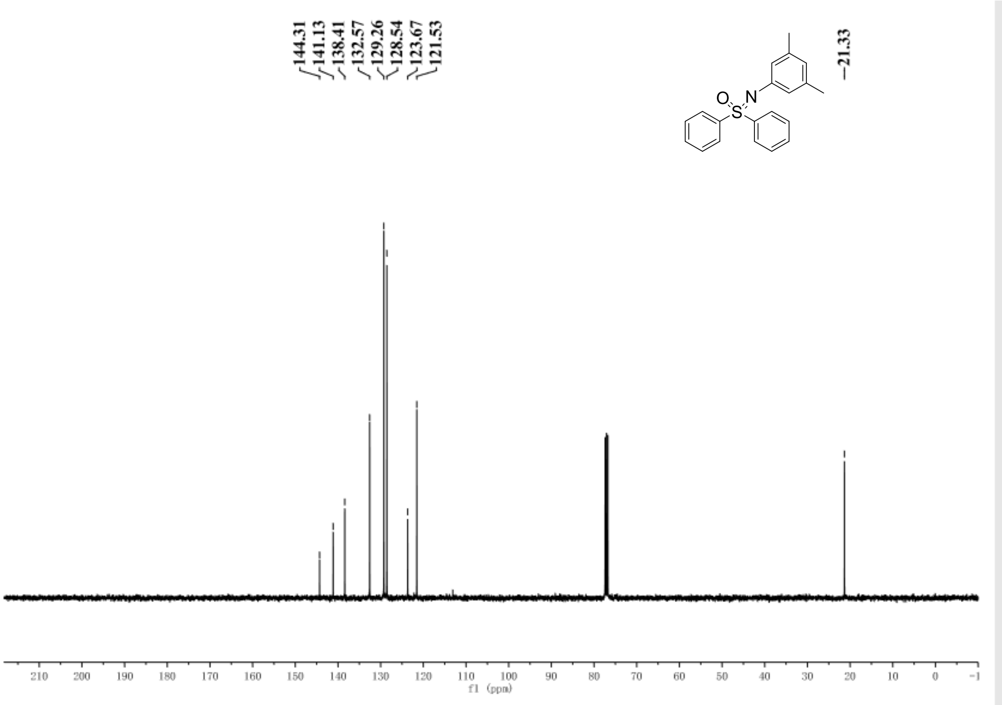

Supplementary Figure 25. $^{13}C\{^1H\}$NMR spectra ($CDCl_3$, 100 MHz) of compound 3ah

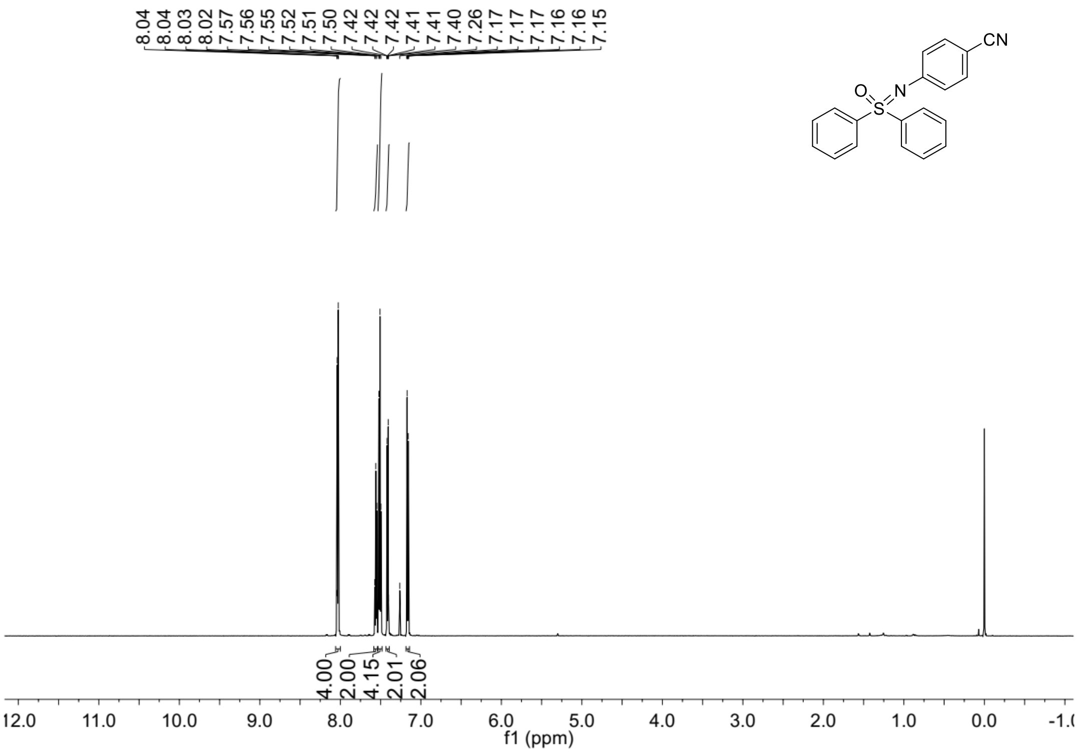

Supplementary Figure 26. **^{1}H NMR spectra ($CDCl_3$, 400 MHz) of compound 3ai**

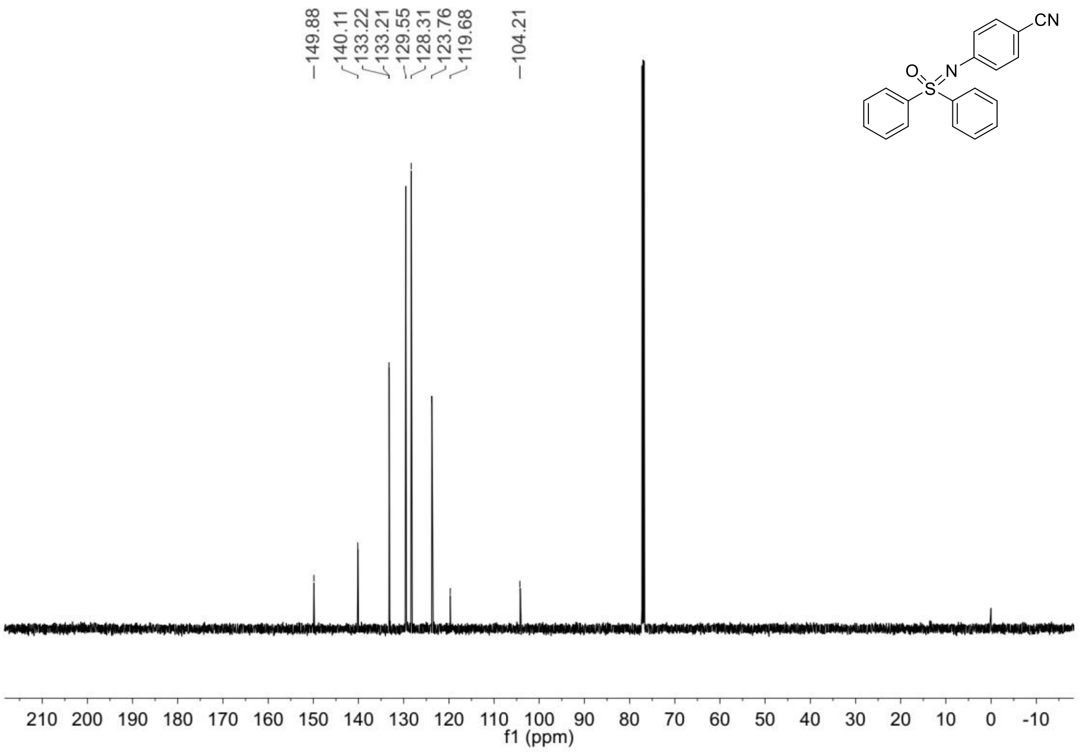

Supplementary Figure 27. **^{13}C{^{1}H}NMR spectra ($CDCl_3$, 100 MHz) of compound 3ai**

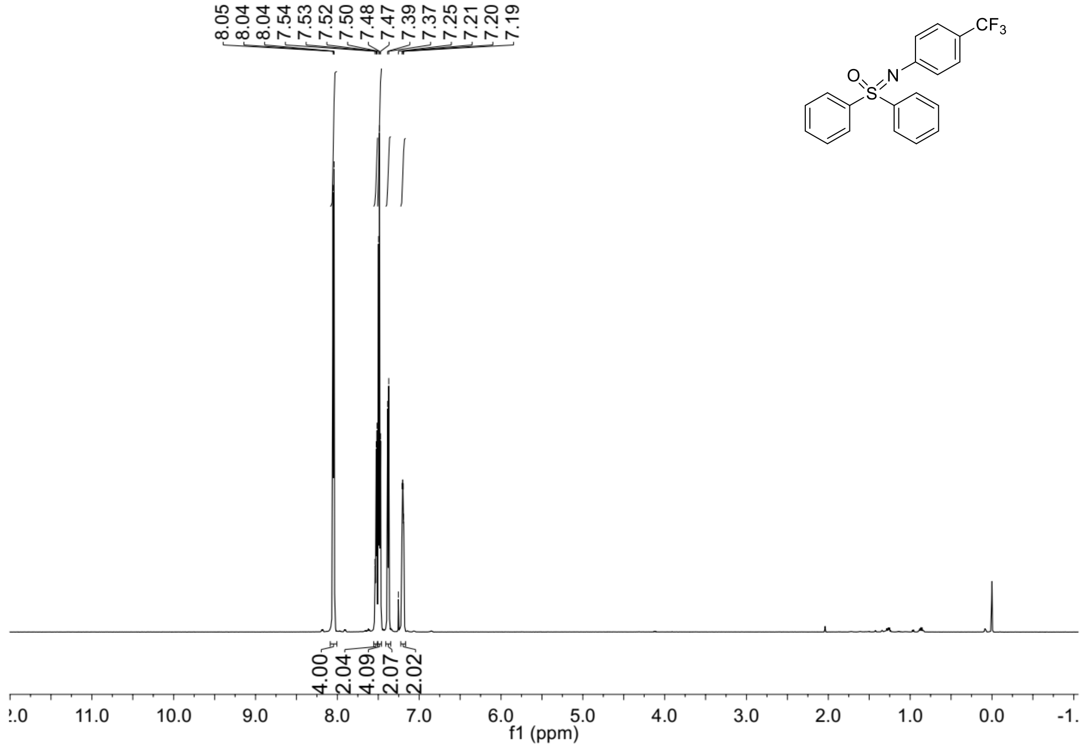

Supplementary Figure 28. ^{1}H NMR spectra ($CDCl_3$, 600 MHz) of compound 3aj

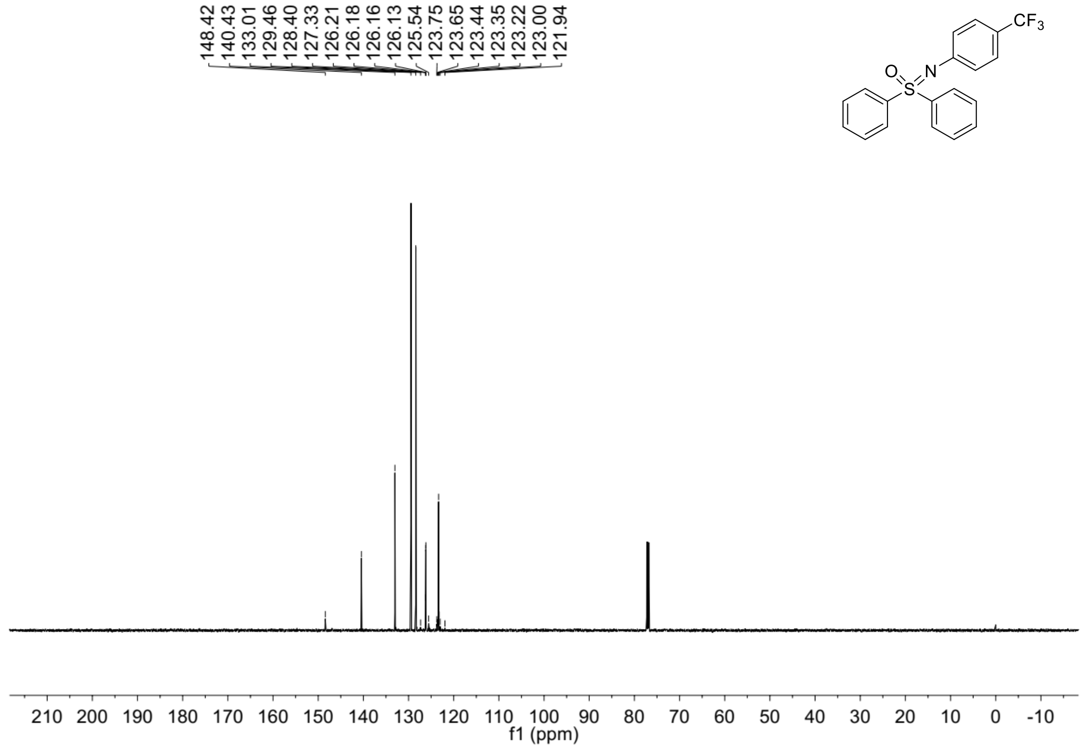

Supplementary Figure 29. ^{13}C{^{1}H}NMR spectra ($CDCl_3$, 150 MHz) of compound 3aj

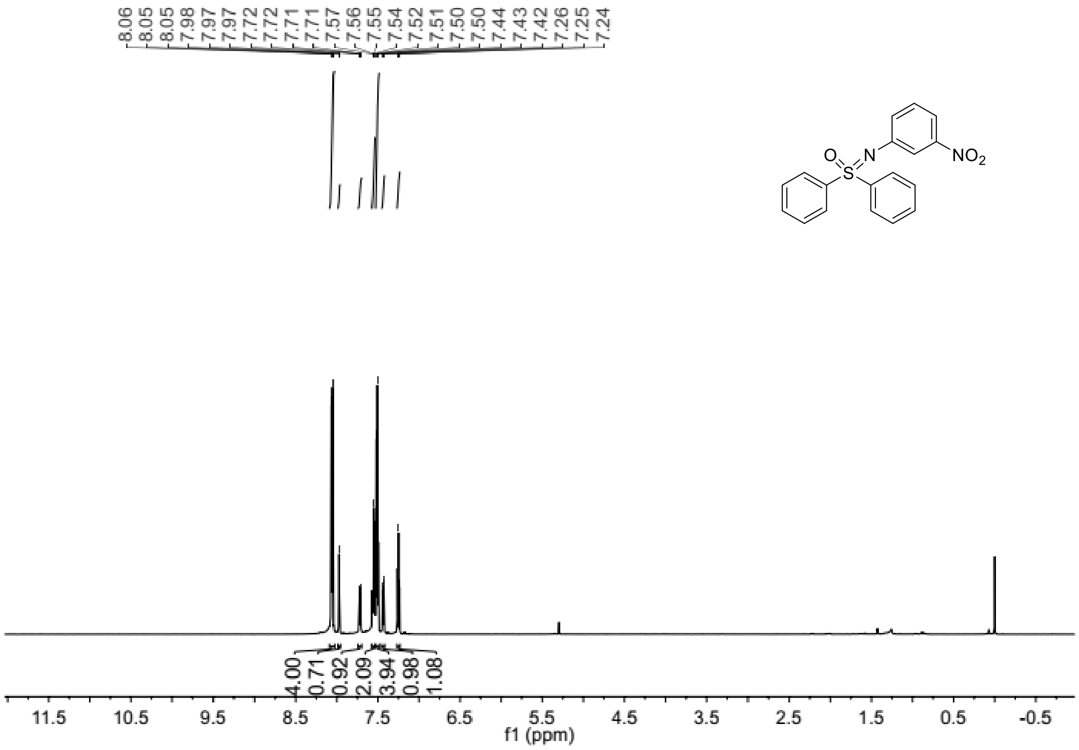

Supplementary Figure 30. ^{1}**H NMR spectra (CDCl$_3$, 600 MHz) of compound 3ak**

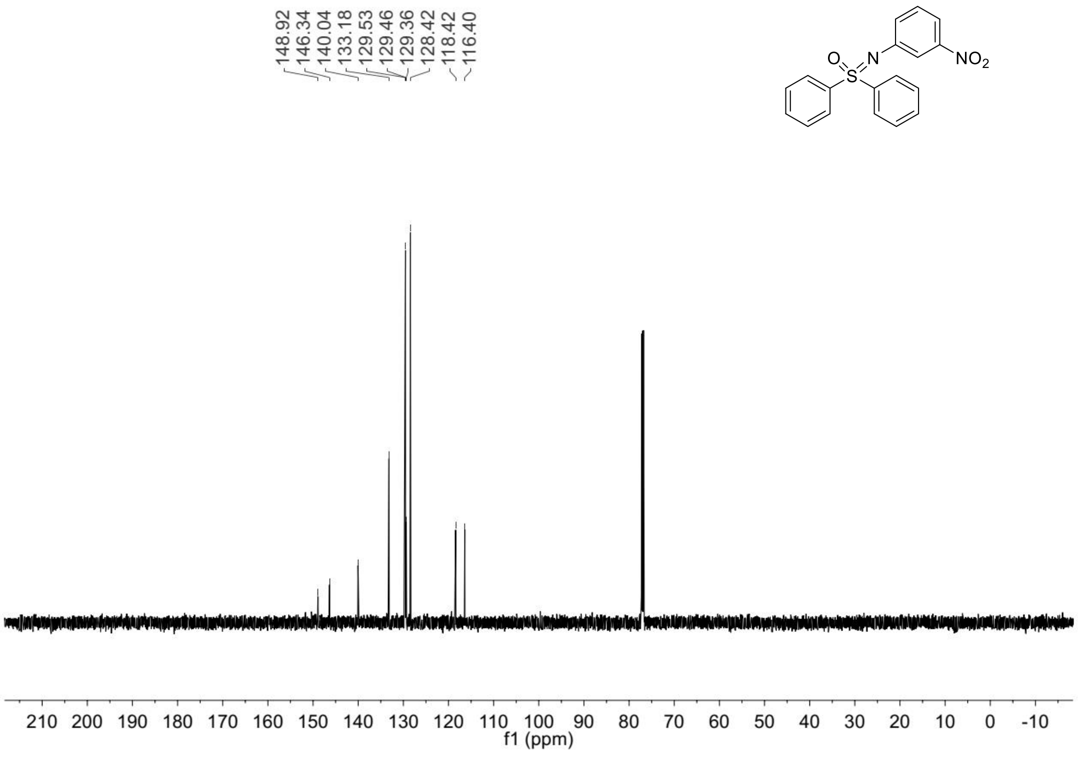

Supplementary Figure 31. ^{13}**C{**^{1}**H}NMR spectra (CDCl$_3$, 150 MHz) of compound 3ak**

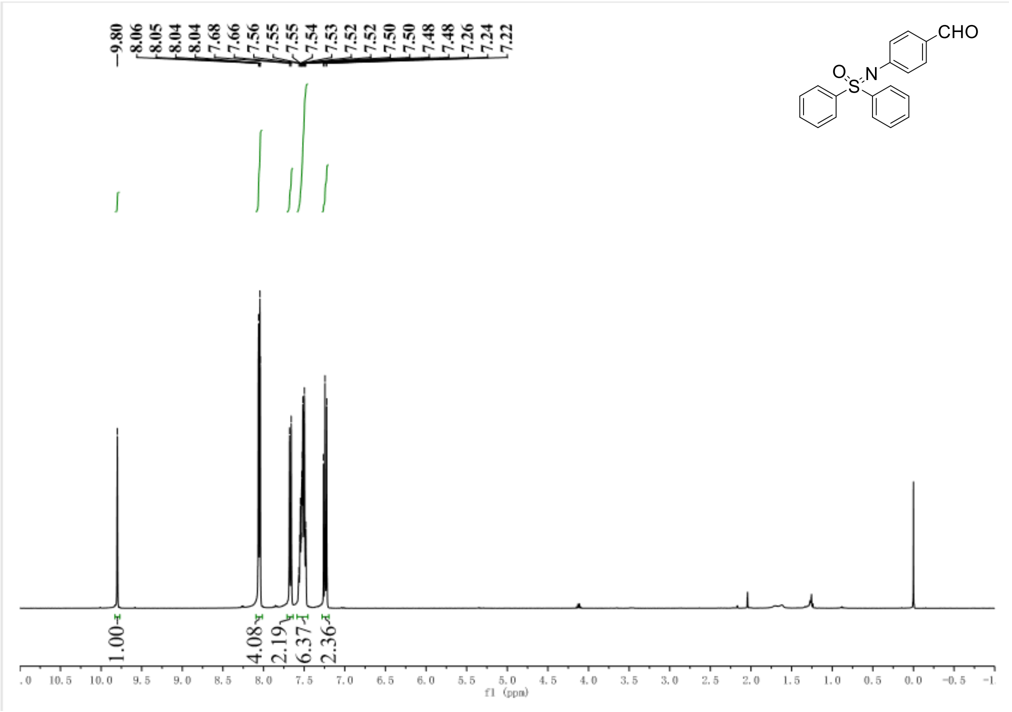

Supplementary Figure 32. ^{1}H NMR spectra ($CDCl_3$, 400 MHz) of compound 3al

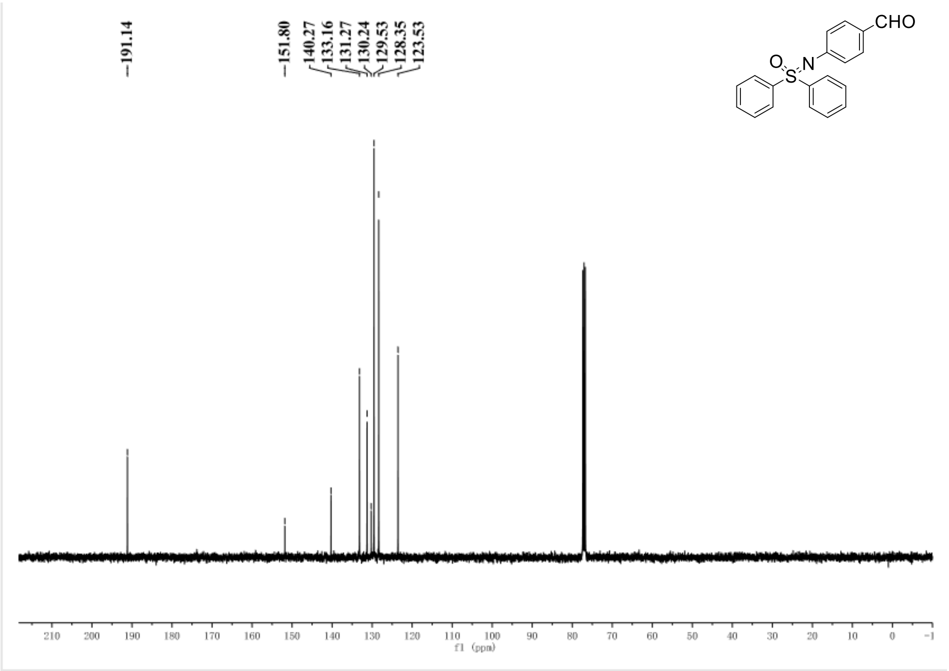

Supplementary Figure 33. $^{13}C\{^{1}H\}$NMR spectra ($CDCl_3$, 100 MHz) of compound 3al

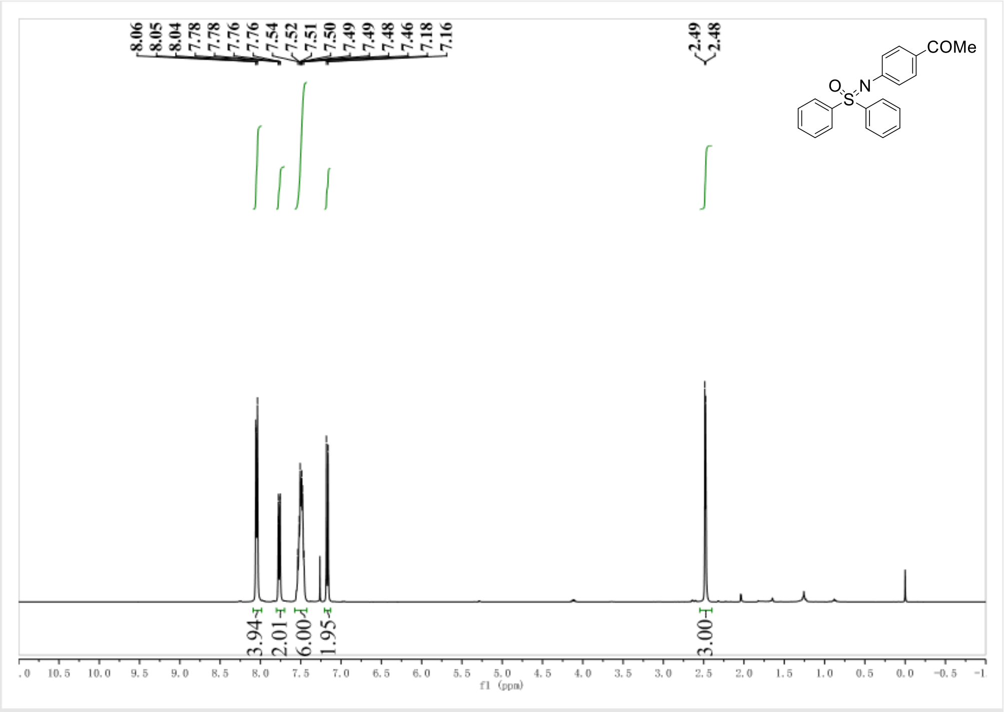

Supplementary Figure 34. **^{1}H NMR spectra ($CDCl_3$, 400 MHz) of compound 3am**

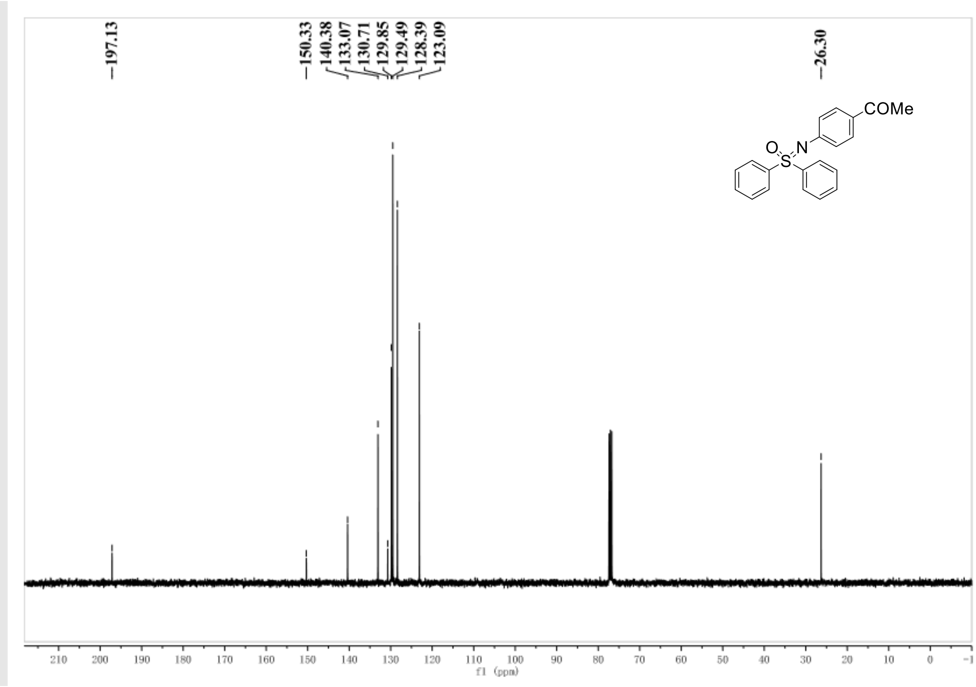

Supplementary Figure 35. **^{13}C{^{1}H}NMR spectra ($CDCl_3$, 100 MHz) of compound 3am**

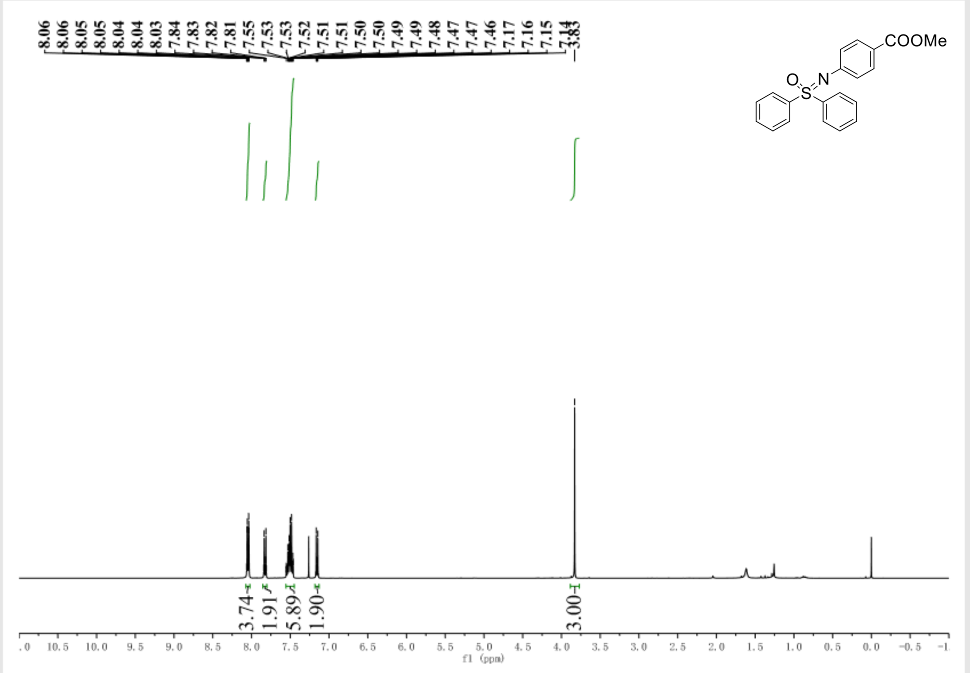

Supplementary Figure 36. ^{1}H NMR spectra ($CDCl_3$, 400 MHz) of compound 3an

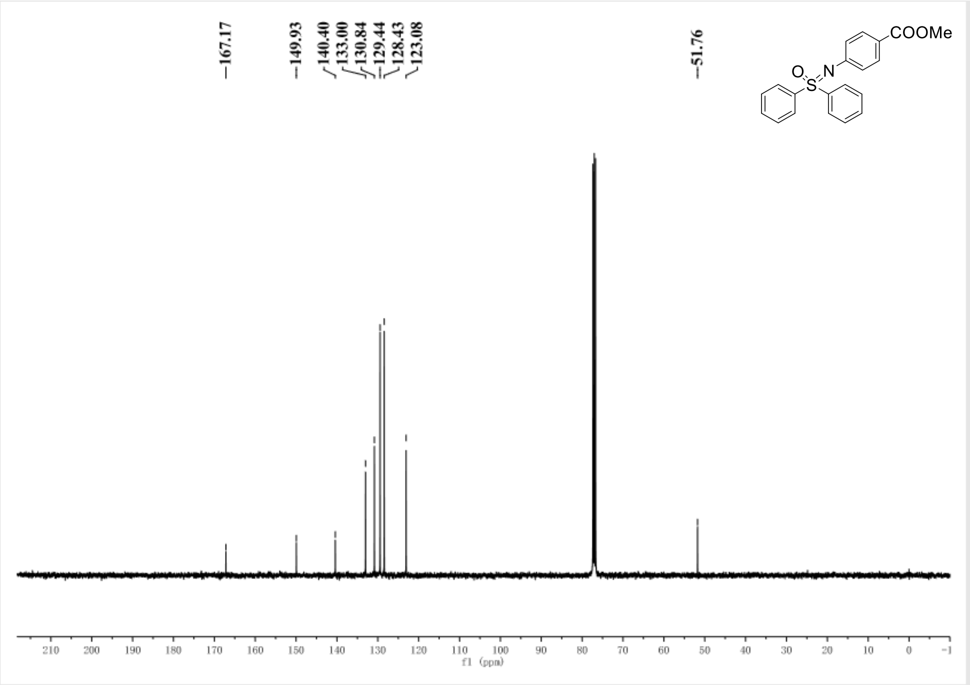

Supplementary Figure 37. $^{13}C\{^{1}H\}$NMR spectra ($CDCl_3$, 100 MHz) of compound 3an

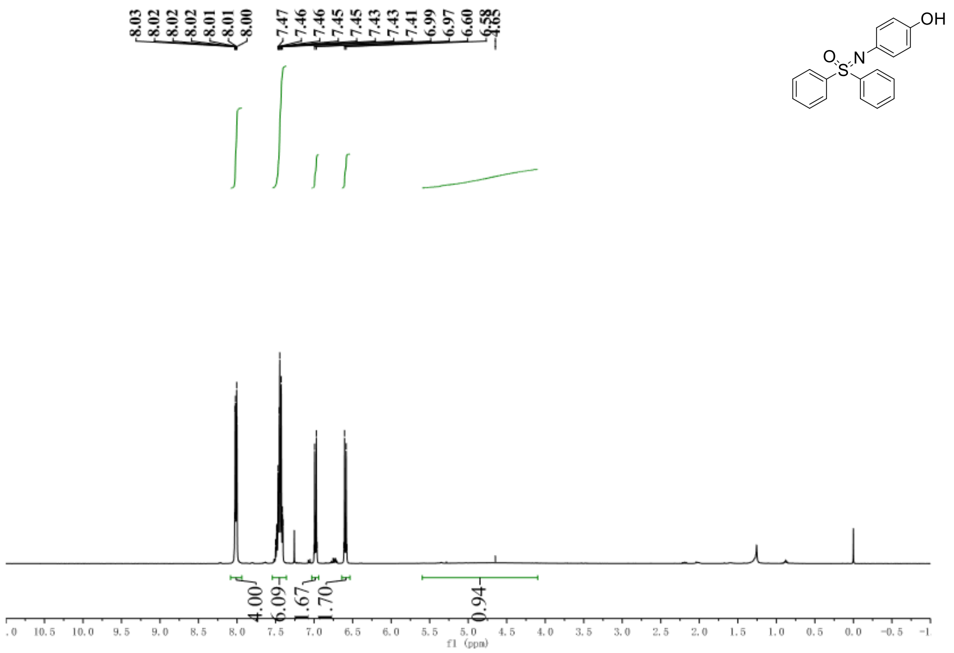

Supplementary Figure 38. ^{1}**H NMR spectra (CDCl$_3$, 400 MHz) of compound 3ao**

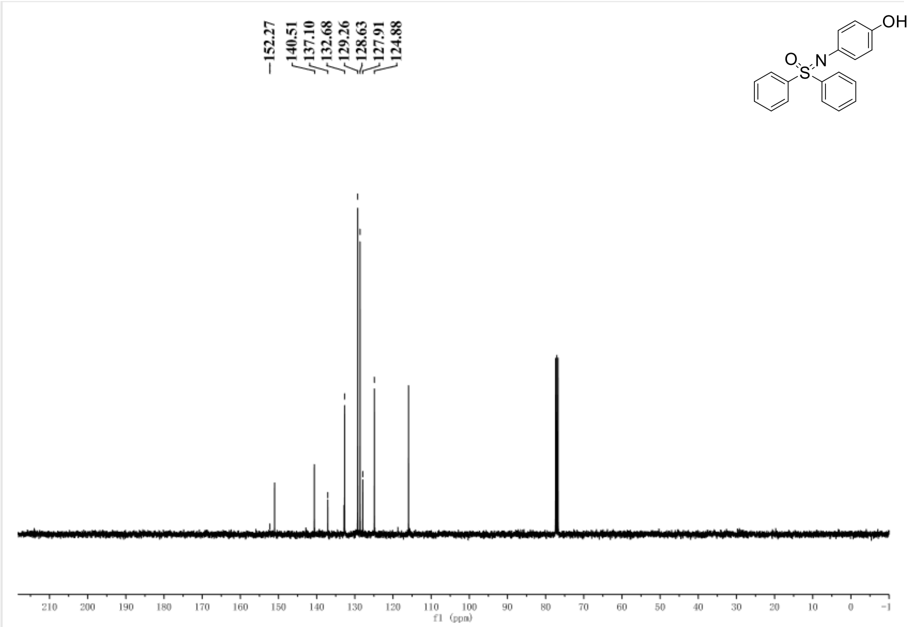

Supplementary Figure 39. ^{13}**C{**^{1}**H}NMR spectra (CDCl$_3$, 100 MHz) of compound 3ao**

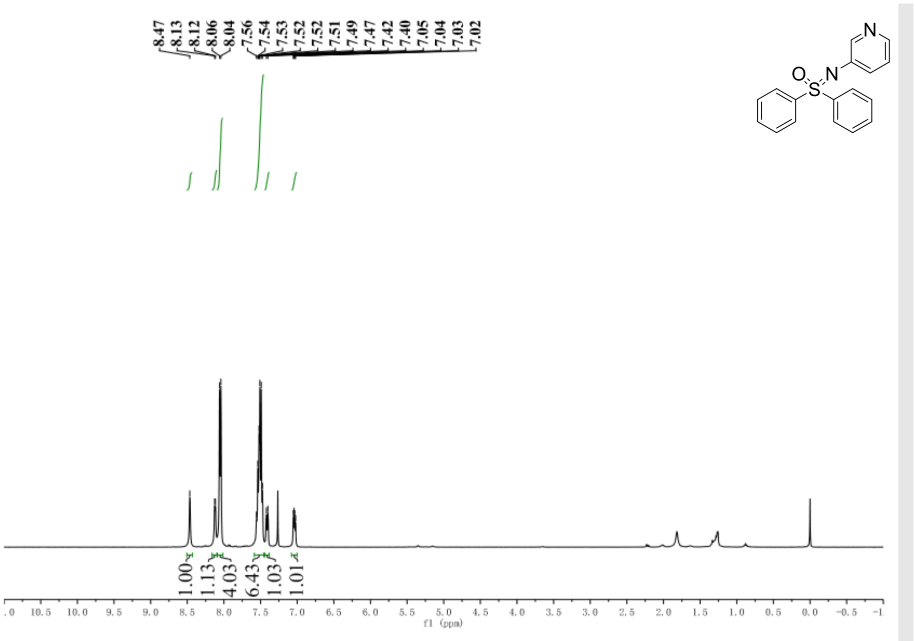

Supplementary Figure 40. ^{1}**H NMR spectra (CDCl$_3$, 400 MHz) of compound 3ap**

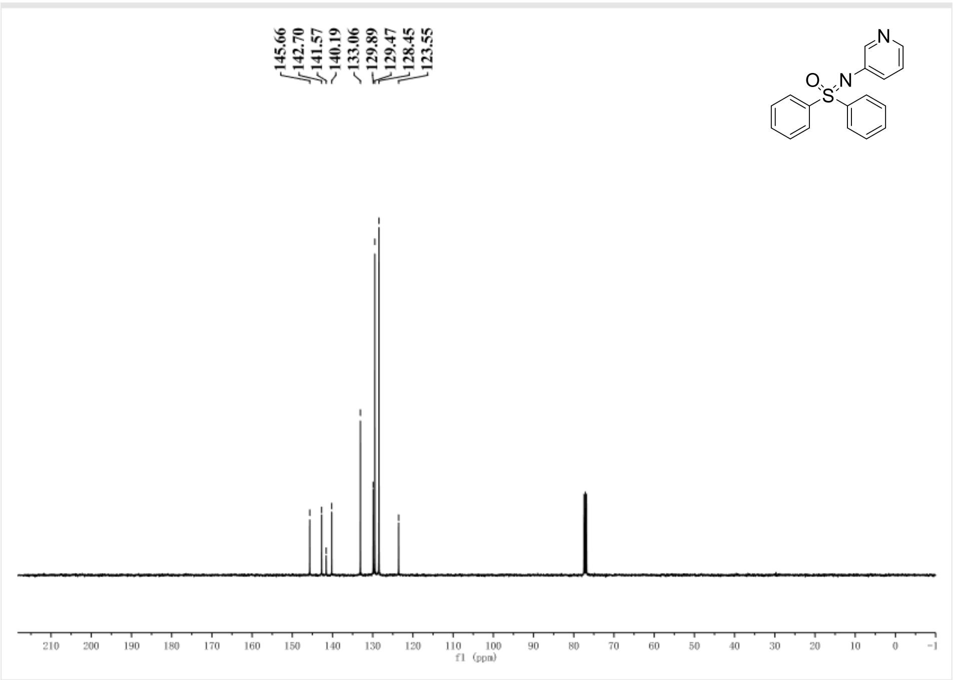

Supplementary Figure 41. ^{13}**C{**^{1}**H}NMR spectra (CDCl$_3$, 100 MHz) of compound 3ap**

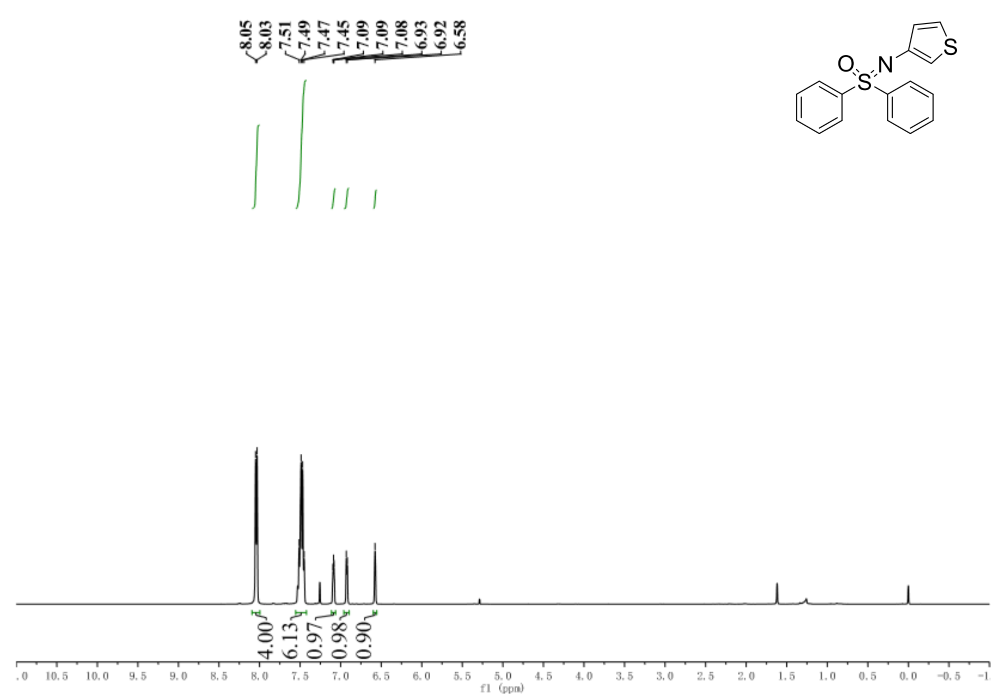

Supplementary Figure 42. ^{1}H NMR spectra ($CDCl_3$, 400 MHz) of compound 3aq

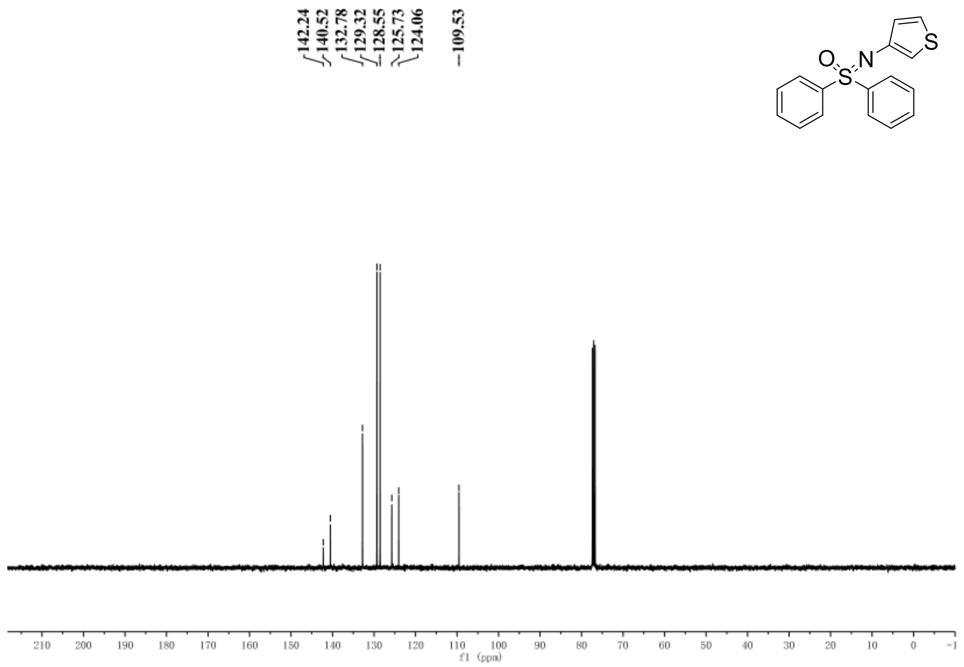

Supplementary Figure 43. ^{13}C{^{1}H}NMR spectra ($CDCl_3$, 100 MHz) of compound 3aq

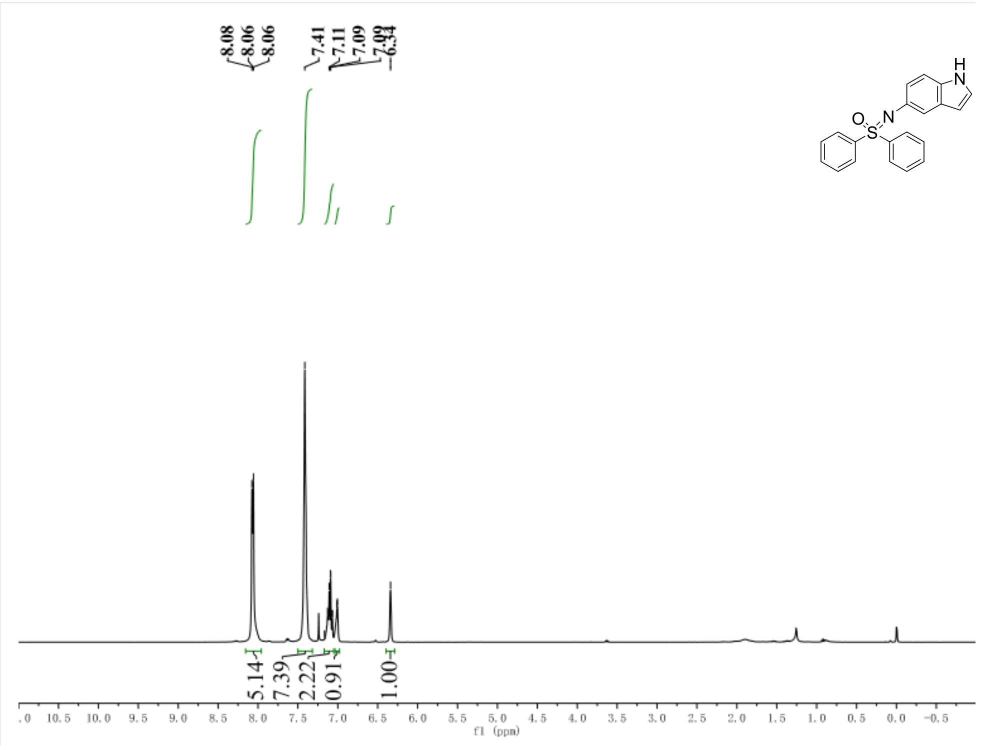

Supplementary Figure 44. ^{1}**H NMR spectra (CDCl**$_3$**, 400 MHz) of compound 3ar**

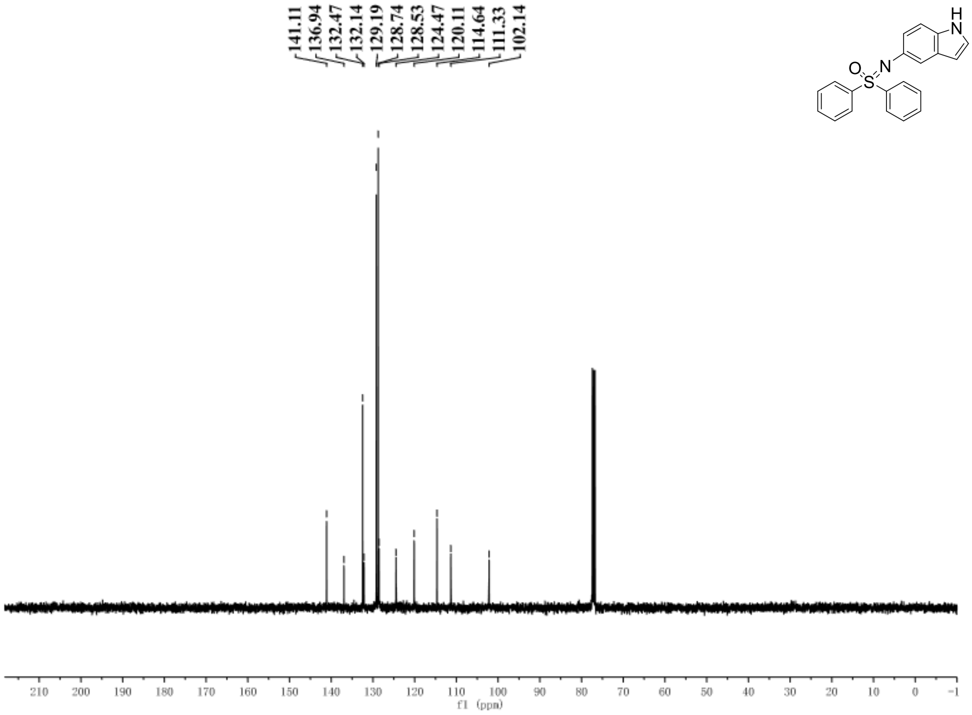

Supplementary Figure 45. ^{13}**C{**^{1}**H}NMR spectra (CDCl**$_3$**, 100 MHz) of compound 3ar**

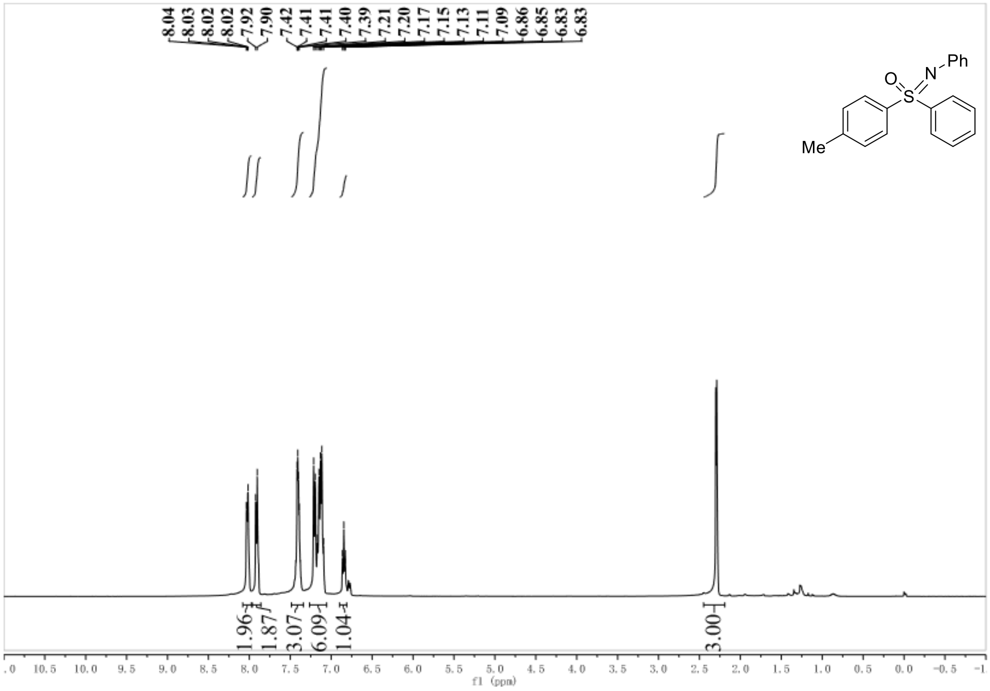

Supplementary Figure 46. ^{1}H NMR spectra ($CDCl_3$, 400 MHz) of compound 3ba

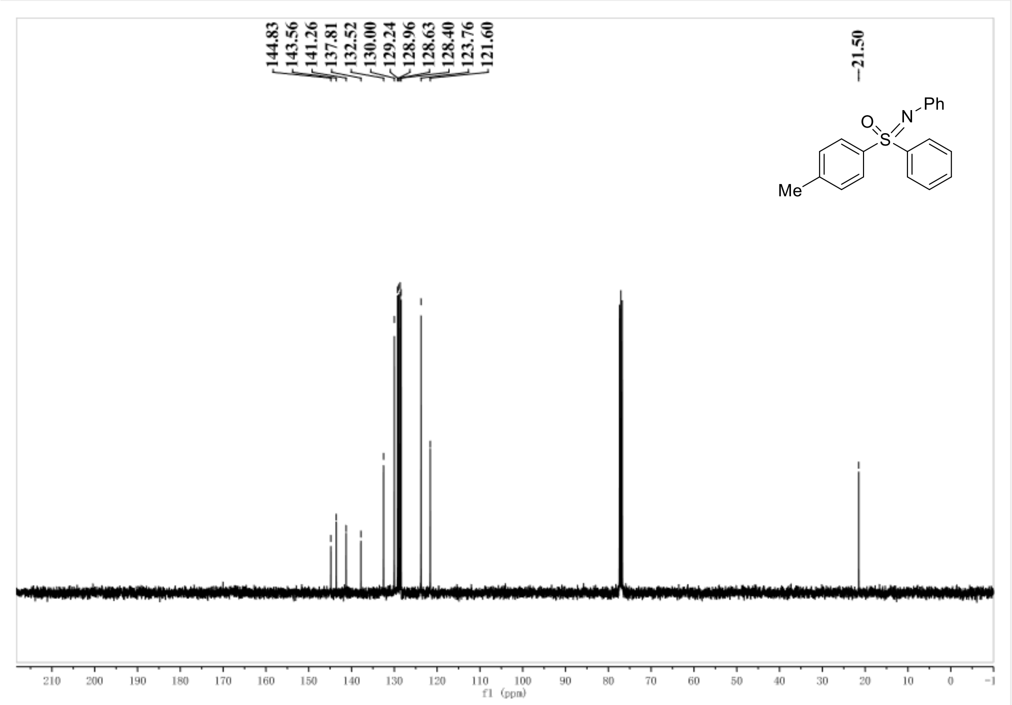

Supplementary Figure 47. $^{13}C\{^{1}H\}$NMR spectra ($CDCl_3$, 100 MHz) of compound 3ba

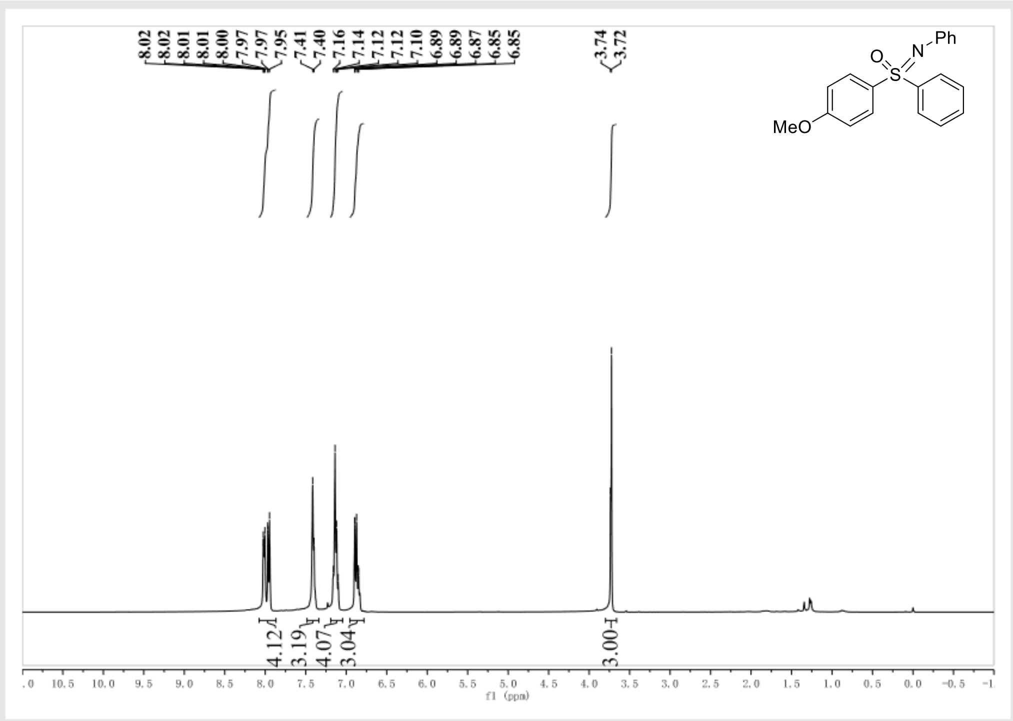

Supplementary Figure 48. ^{1}H NMR spectra ($CDCl_3$, 400 MHz) of compound 3ca

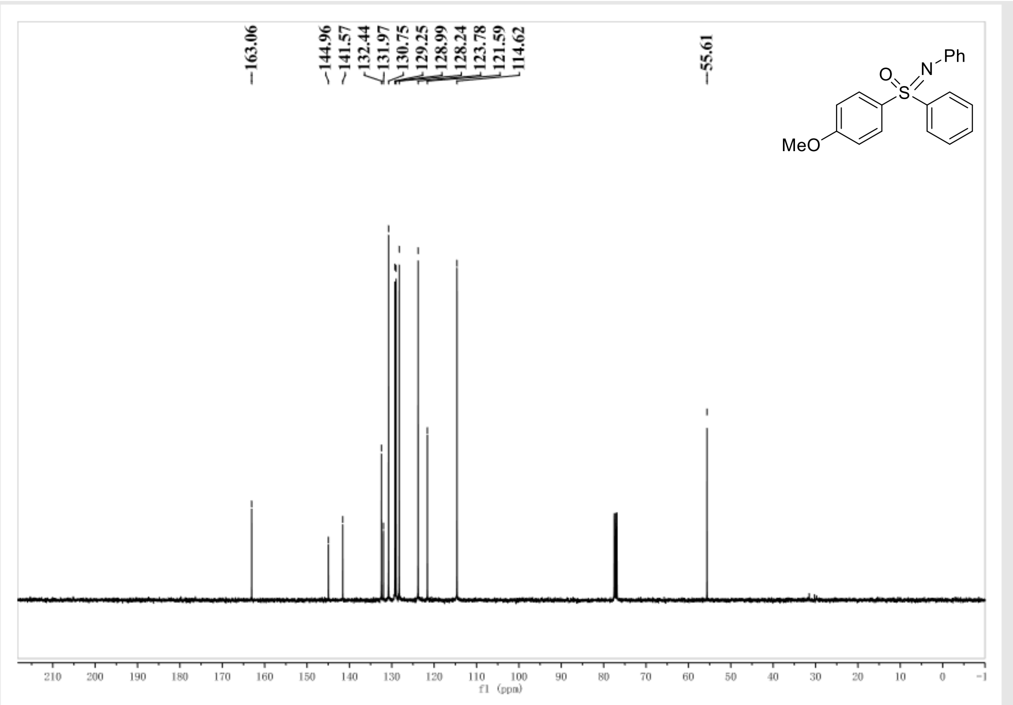

Supplementary Figure 49. $^{13}C\{^{1}H\}$NMR spectra ($CDCl_3$, 100 MHz) of compound 3ca

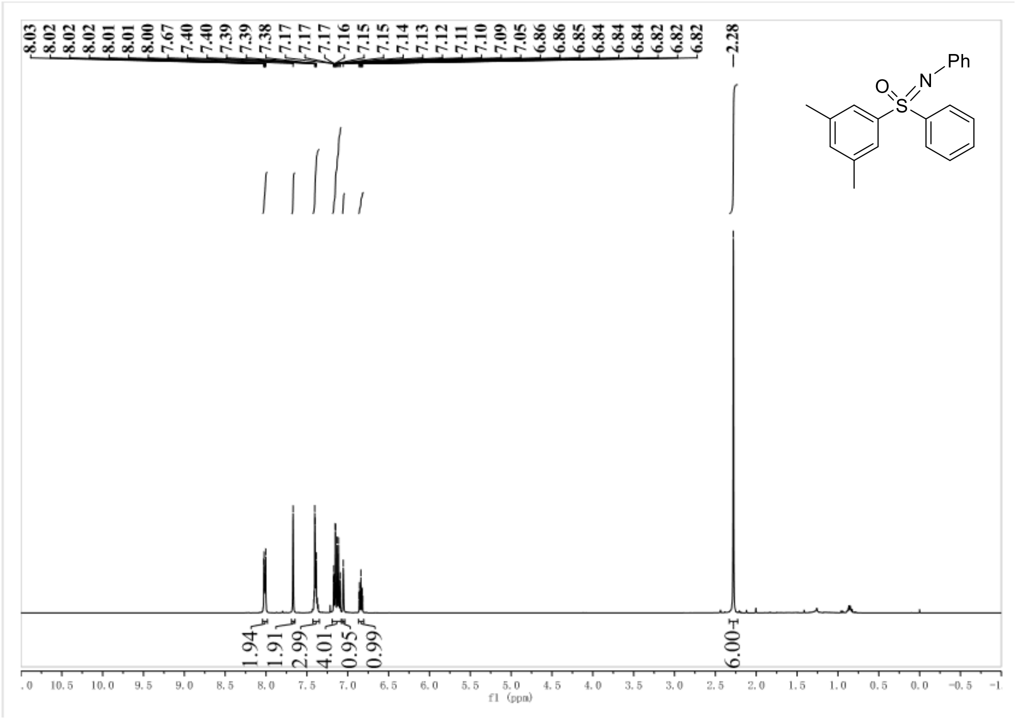

Supplementary Figure 50. ^{1}H NMR spectra ($CDCl_3$, 400 MHz) of compound 3da

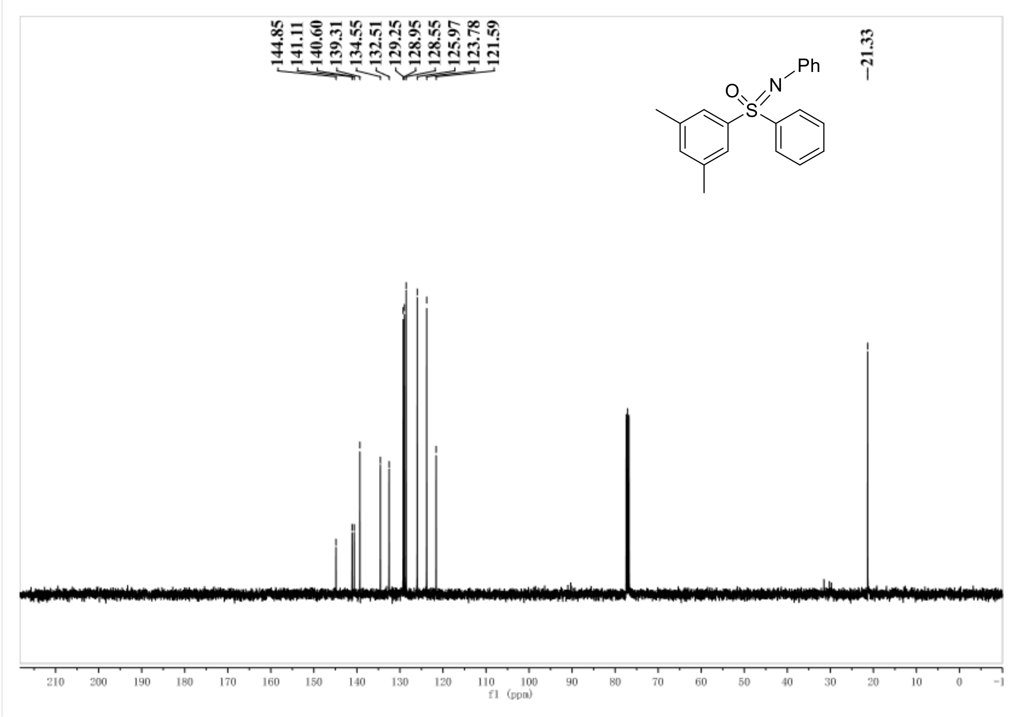

Supplementary Figure 51. $^{13}C\{^{1}H\}$NMR spectra ($CDCl_3$, 100 MHz) of compound 3da

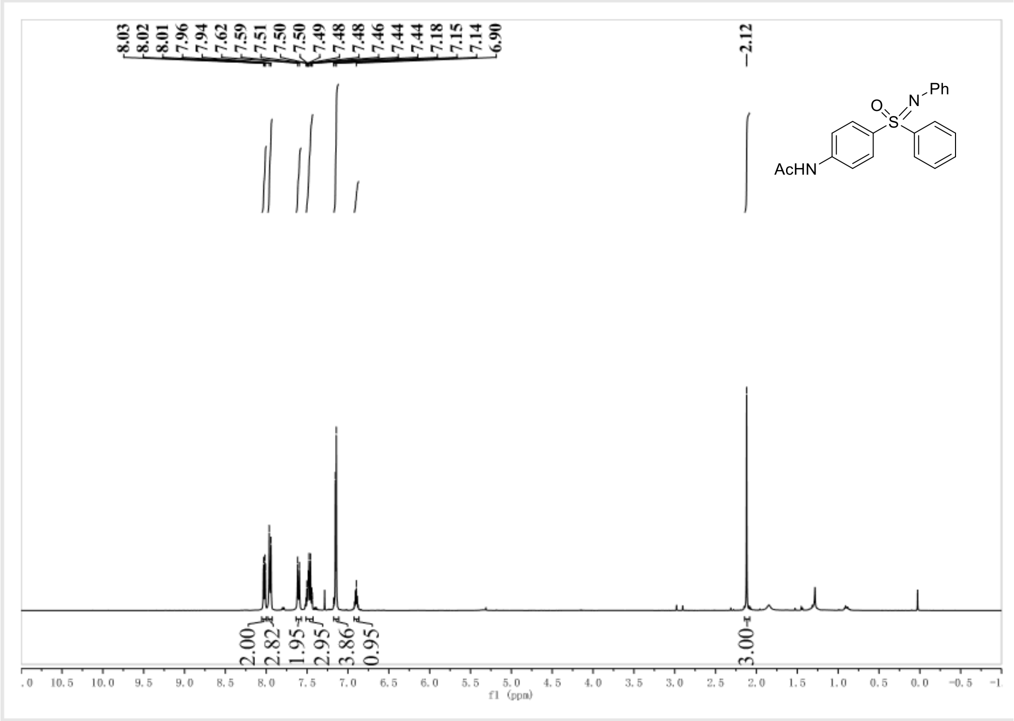

Supplementary Figure 52. ^{1}H NMR spectra ($CDCl_3$, 400 MHz) of compound 3ea

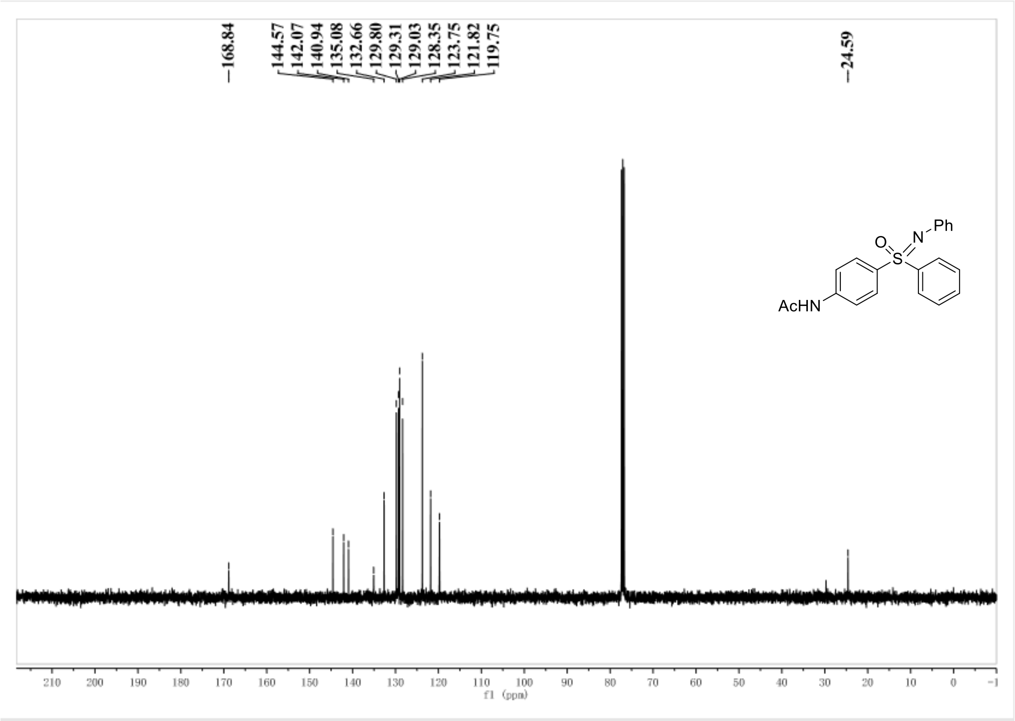

Supplementary Figure 53. ^{13}C{^{1}H}NMR spectra ($CDCl_3$, 100 MHz) of compound 3ea

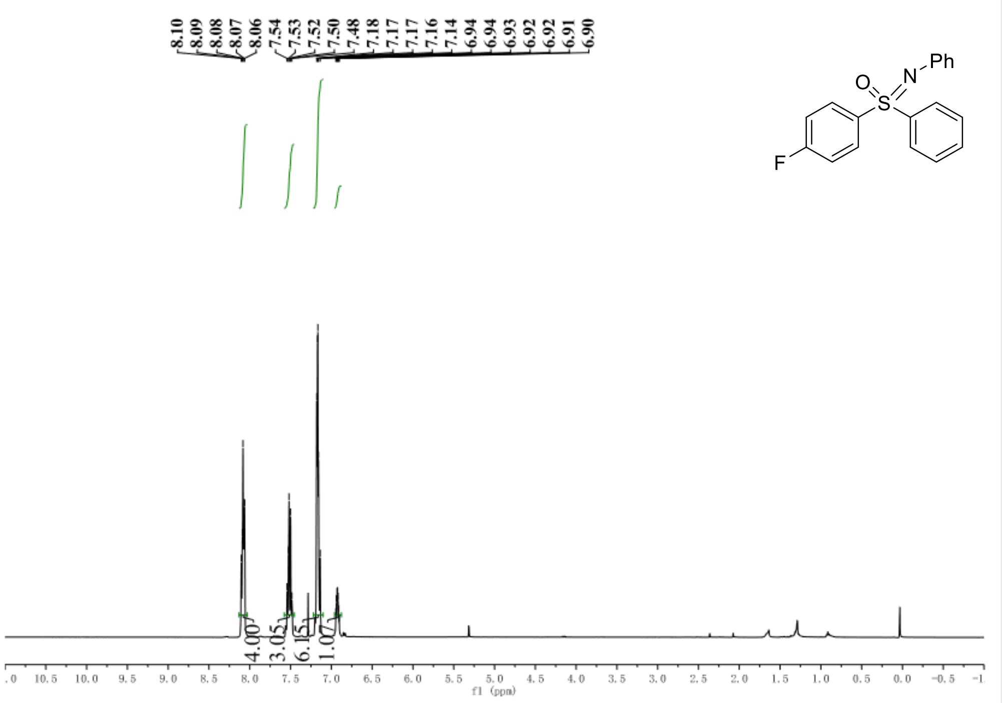

Supplementary Figure 54. ^{1}**H NMR spectra (CDCl**$_3$**, 400 MHz) of compound 3fa**

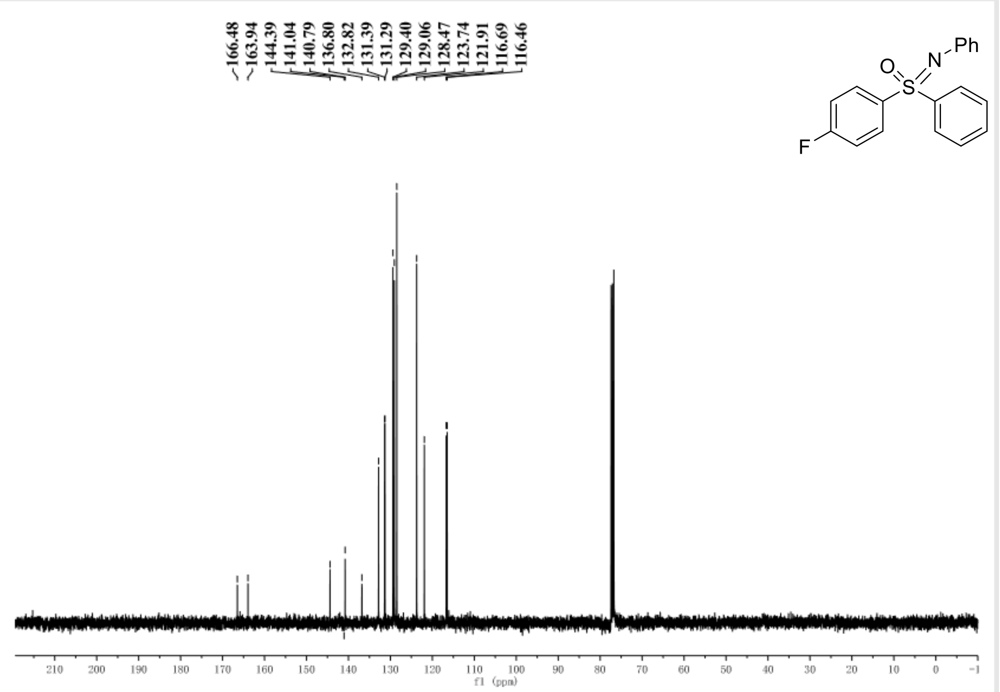

Supplementary Figure 55. ^{13}**C{**^{1}**H}NMR spectra (CDCl**$_3$**, 100 MHz) of compound 3fa**

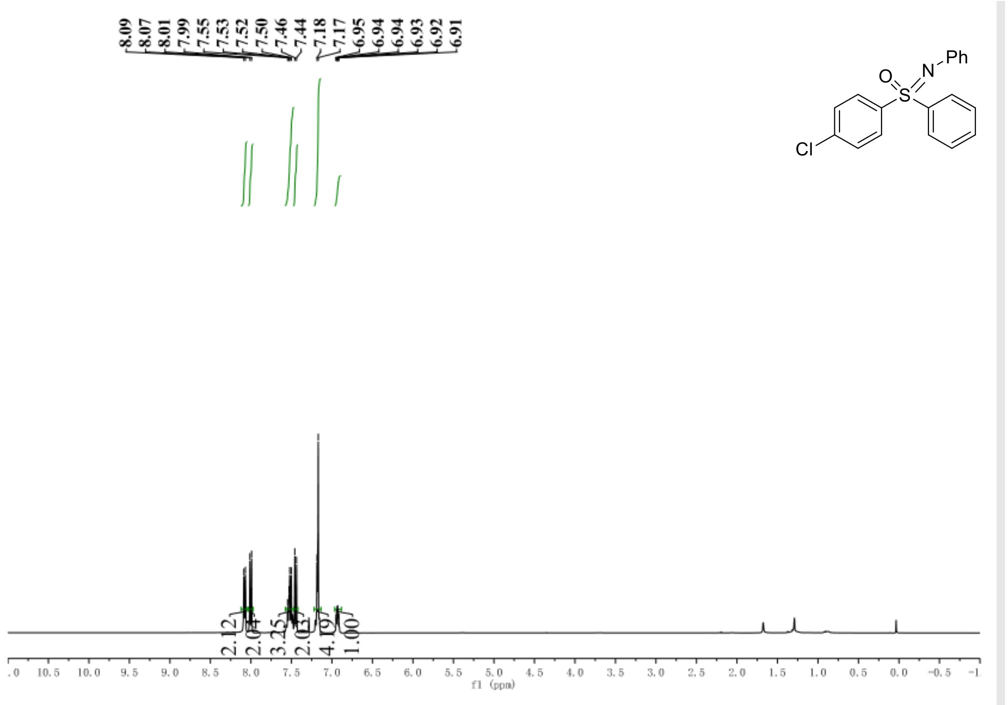

Supplementary Figure 56. ^{1}H NMR spectra ($CDCl_3$, 400 MHz) of compound 3ga

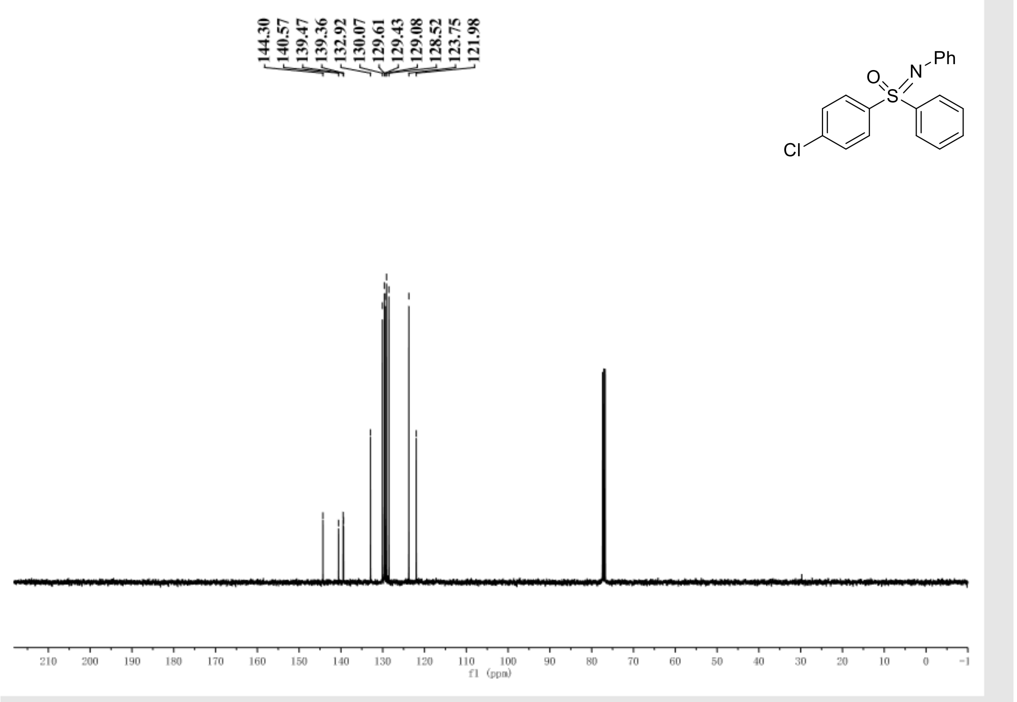

Supplementary Figure 57. $^{13}C\{^{1}H\}$NMR spectra ($CDCl_3$, 100 MHz) of compound 3ga

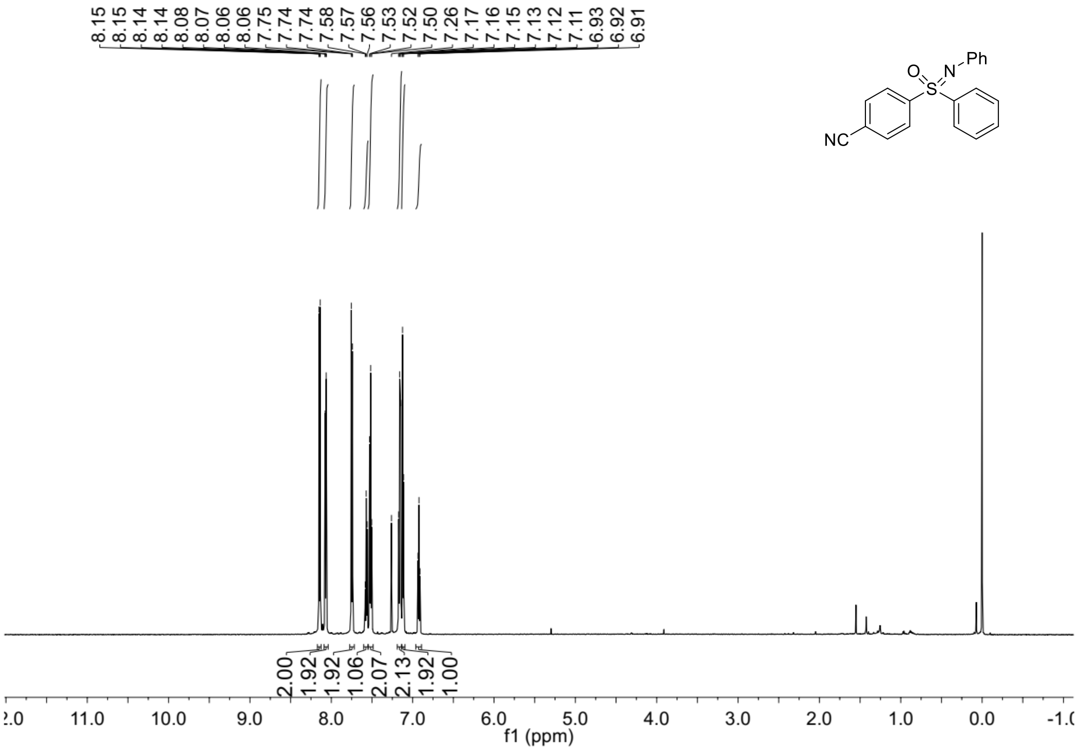

Supplementary Figure 58. ^{1}H NMR spectra (CDCl$_3$, 600 MHz) of compound 3ha

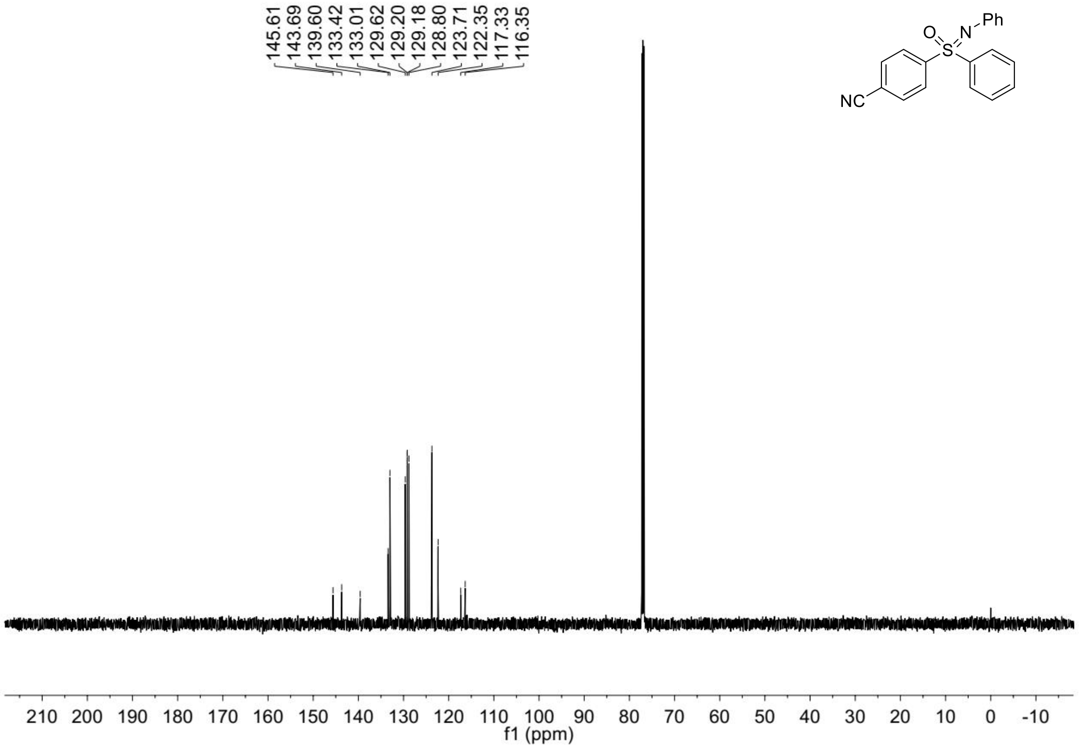

Supplementary Figure 59. ^{13}C{^{1}H}NMR spectra (CDCl$_3$, 150 MHz) of compound 3ha

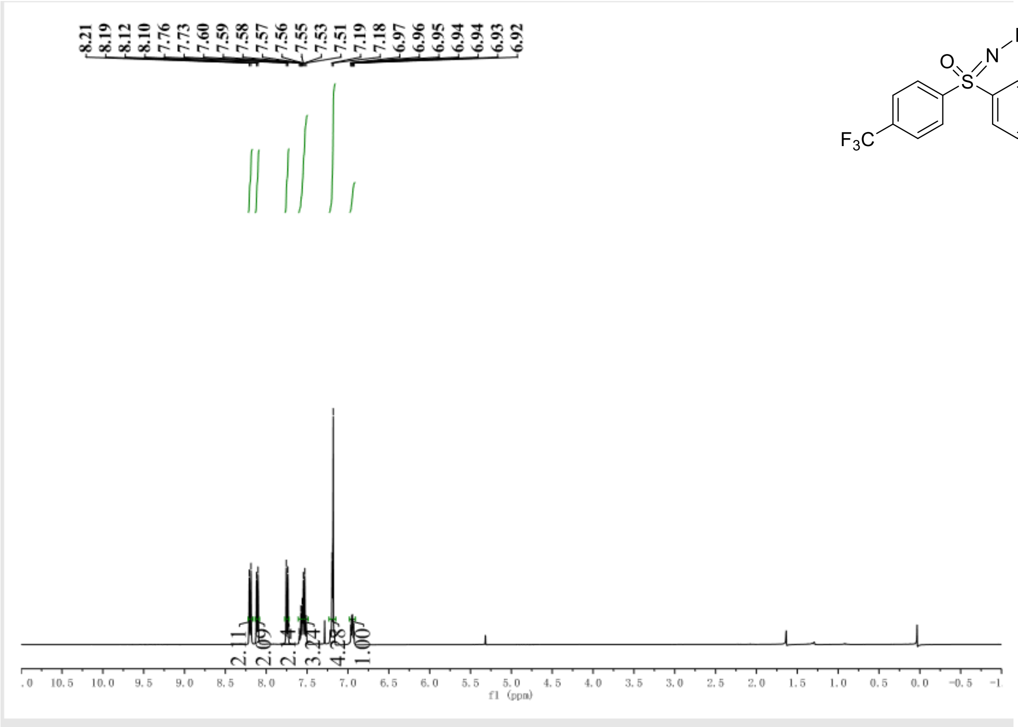

Supplementary Figure 60. ^{1}H NMR spectra ($CDCl_3$, 400 MHz) of compound 3ia

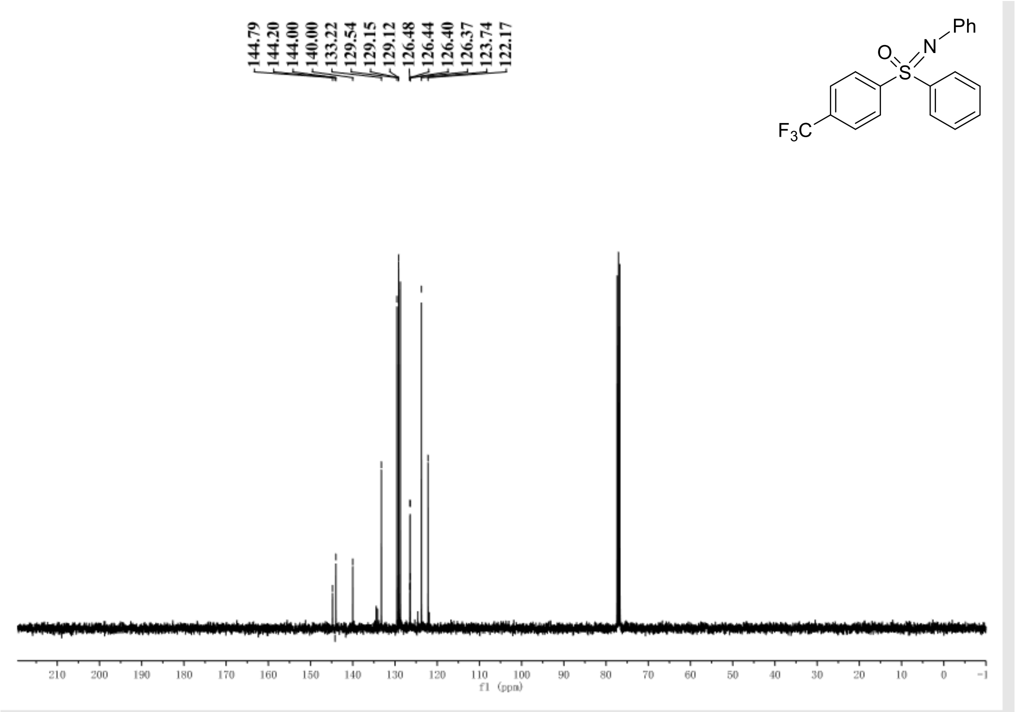

Supplementary Figure 61. ^{13}C{^{1}H}NMR spectra ($CDCl_3$, 100 MHz) of compound 3ia

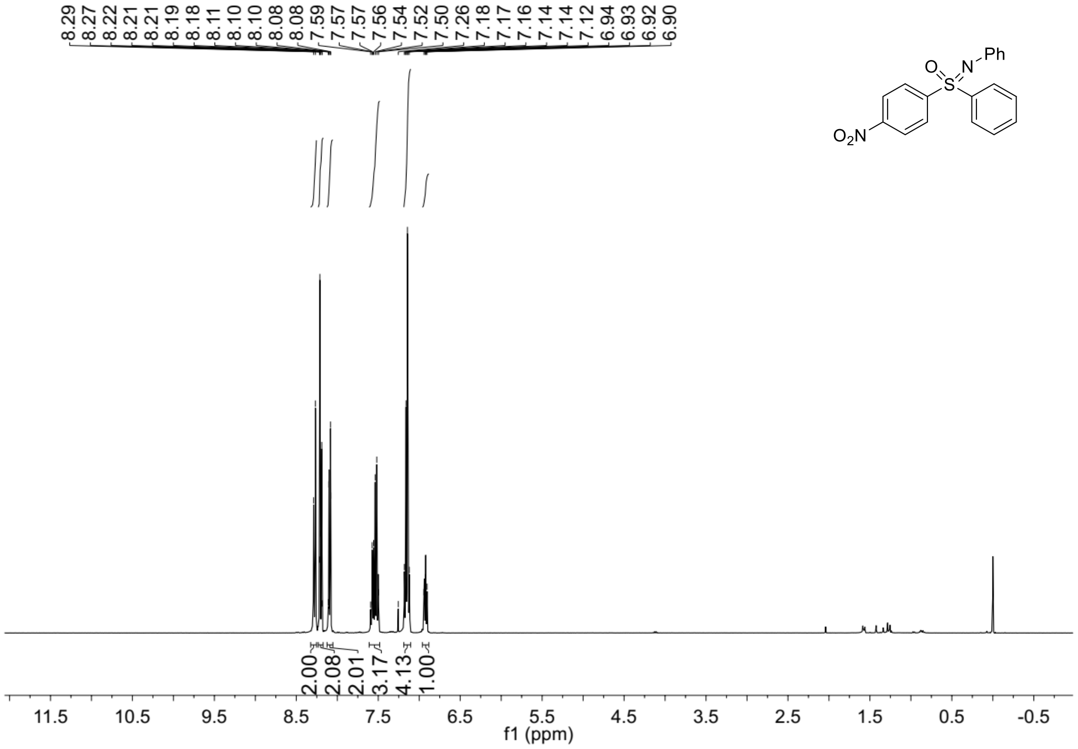

Supplementary Figure 62. ^{1}H NMR spectra ($CDCl_3$, 600 MHz) of compound 3ja

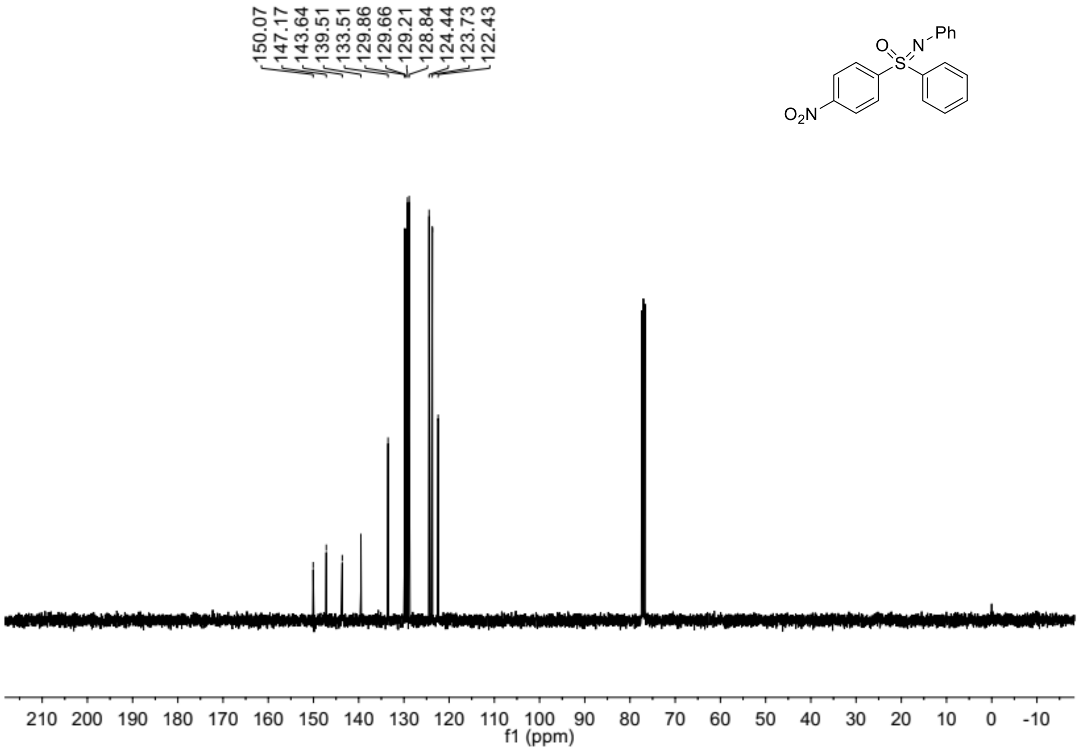

Supplementary Figure 63. $^{13}C\{^{1}H\}$NMR spectra ($CDCl_3$, 150 MHz) of compound 3ja

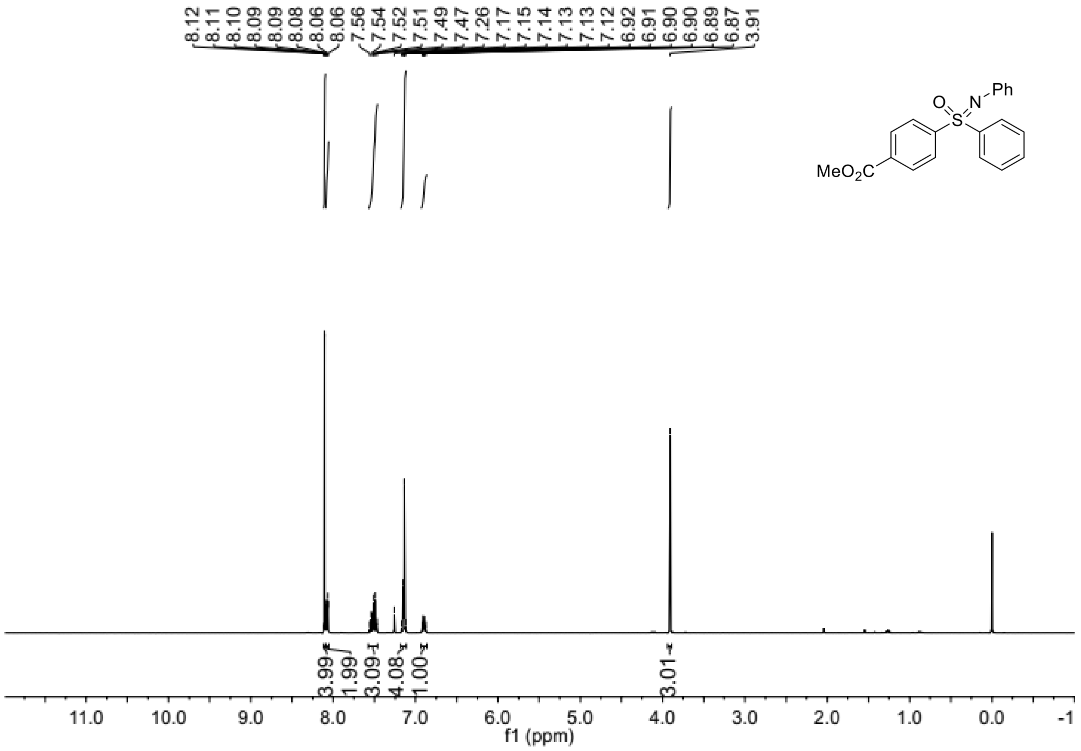

Supplementary Figure 64. ^{1}H NMR spectra ($CDCl_3$, 600 MHz) of compound 3ka

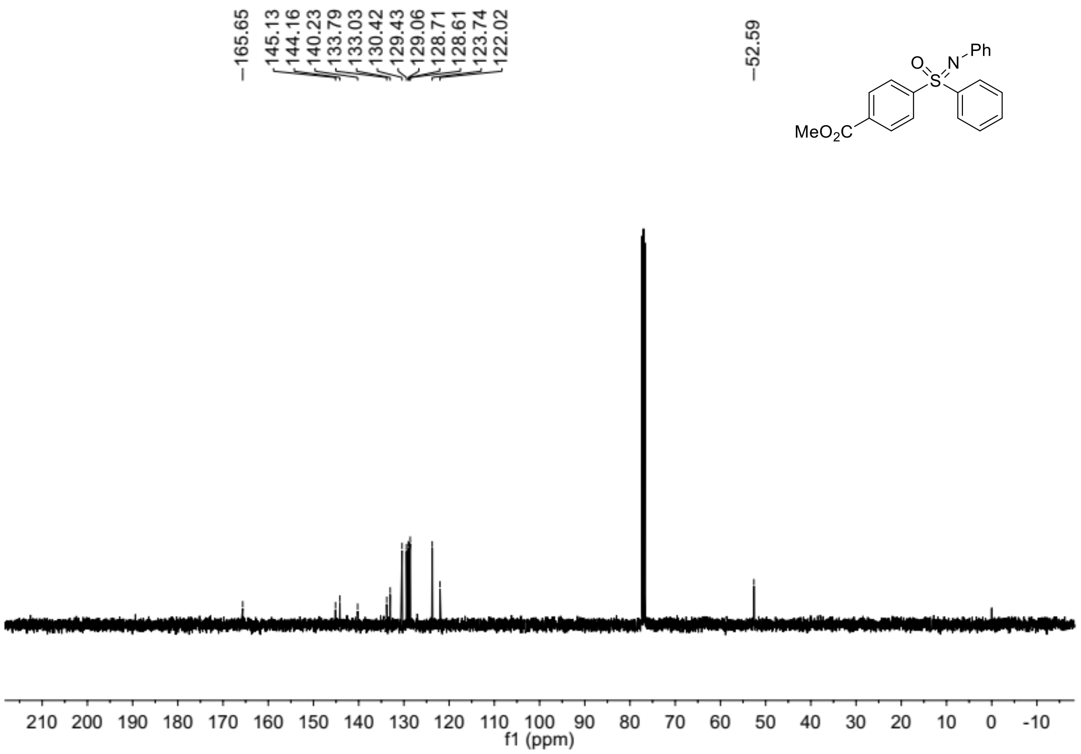

Supplementary Figure 65. ^{13}C{^{1}H}NMR spectra ($CDCl_3$, 150 MHz) of compound 3ka

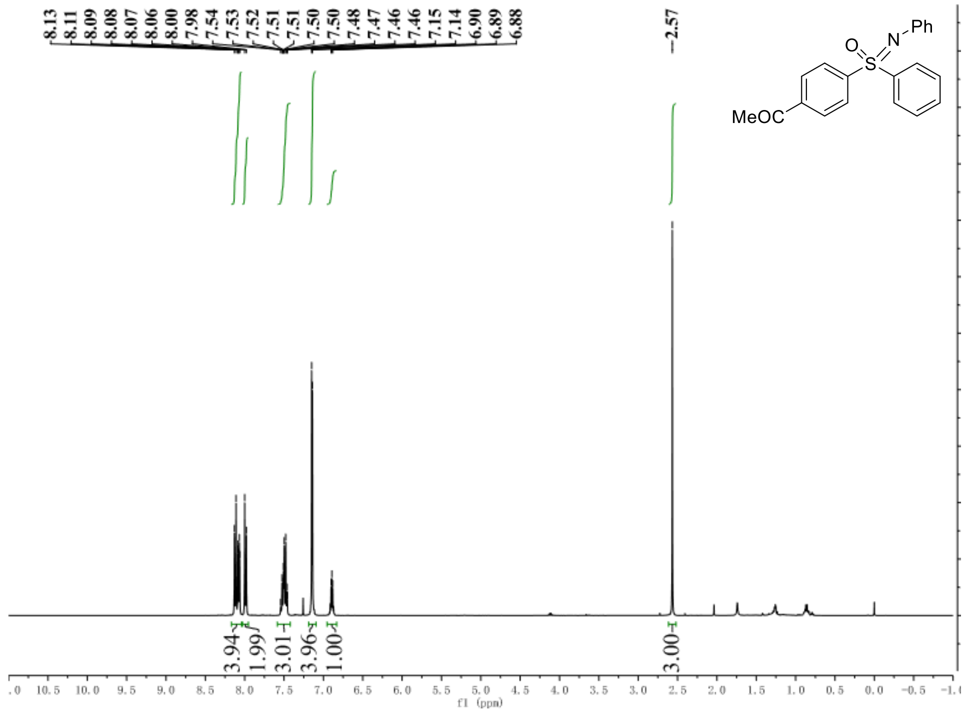

Supplementary Figure 66. ^{1}H NMR spectra ($CDCl_3$, 400 MHz) of compound 3la

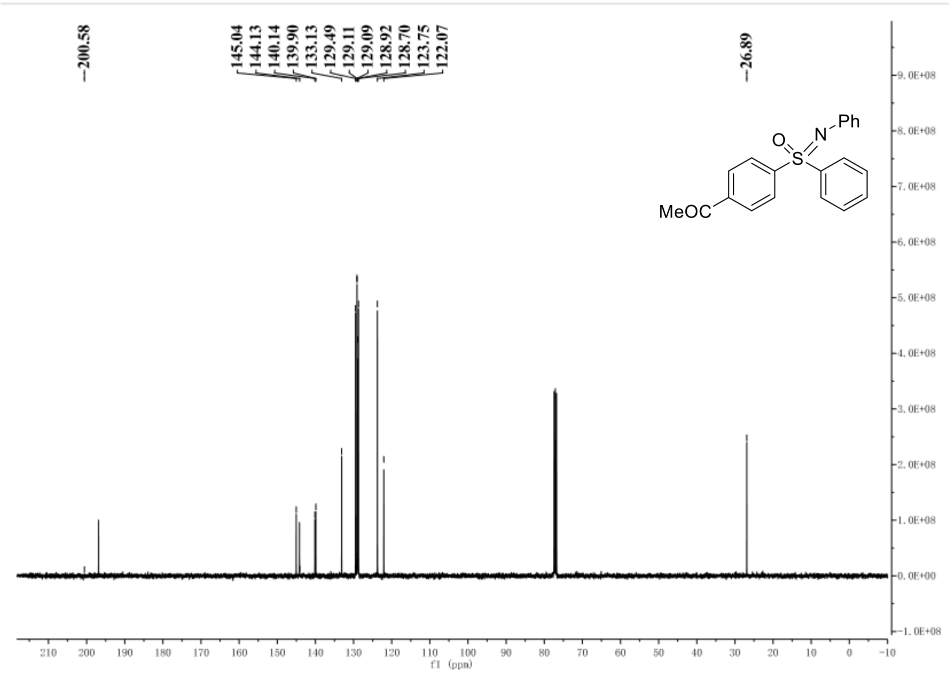

Supplementary Figure 67. $^{13}C\{^{1}H\}$NMR spectra ($CDCl_3$, 100 MHz) of compound 3la

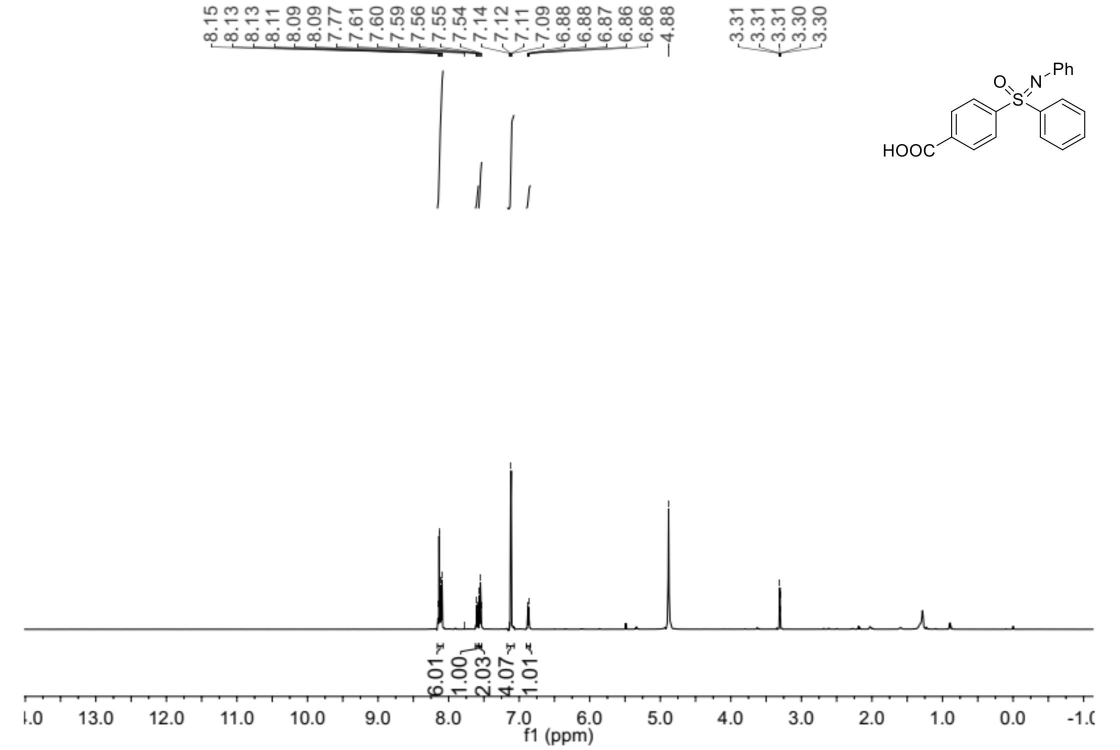

Supplementary Figure 68. ^{1}**H NMR spectra (CDCl$_3$, 600 MHz) of compound 3ma**

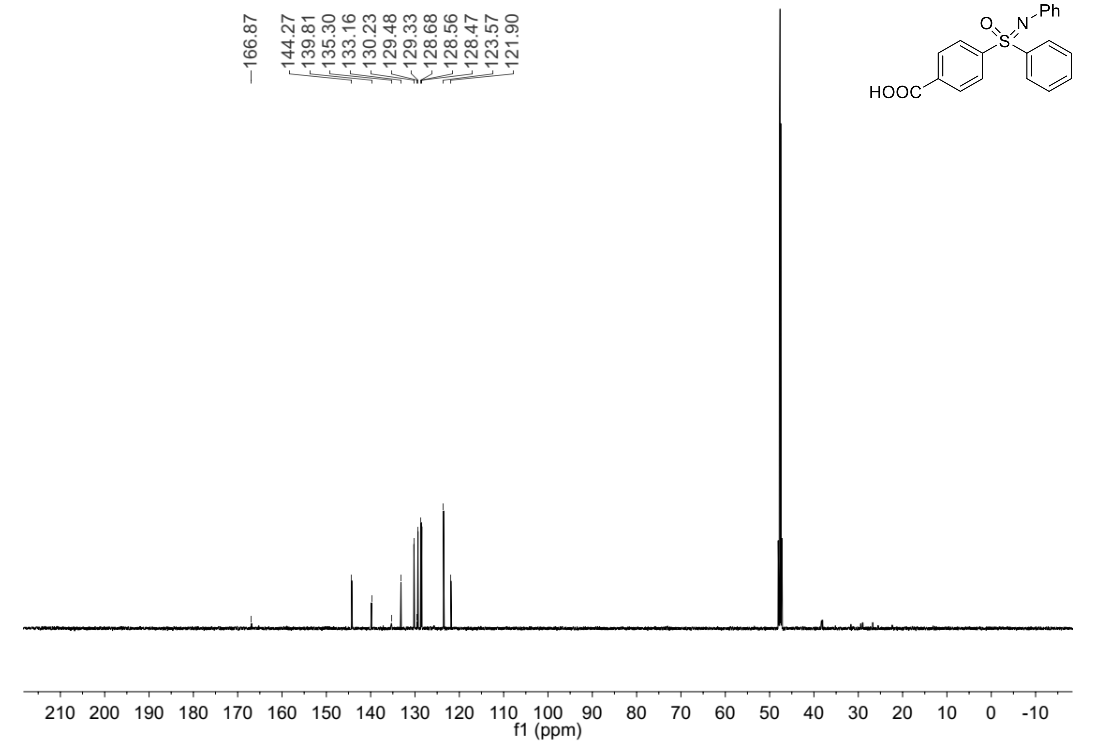

Supplementary Figure 69. ^{13}**C{**^{1}**H}NMR spectra (CDCl$_3$, 150 MHz) of compound 3ma**

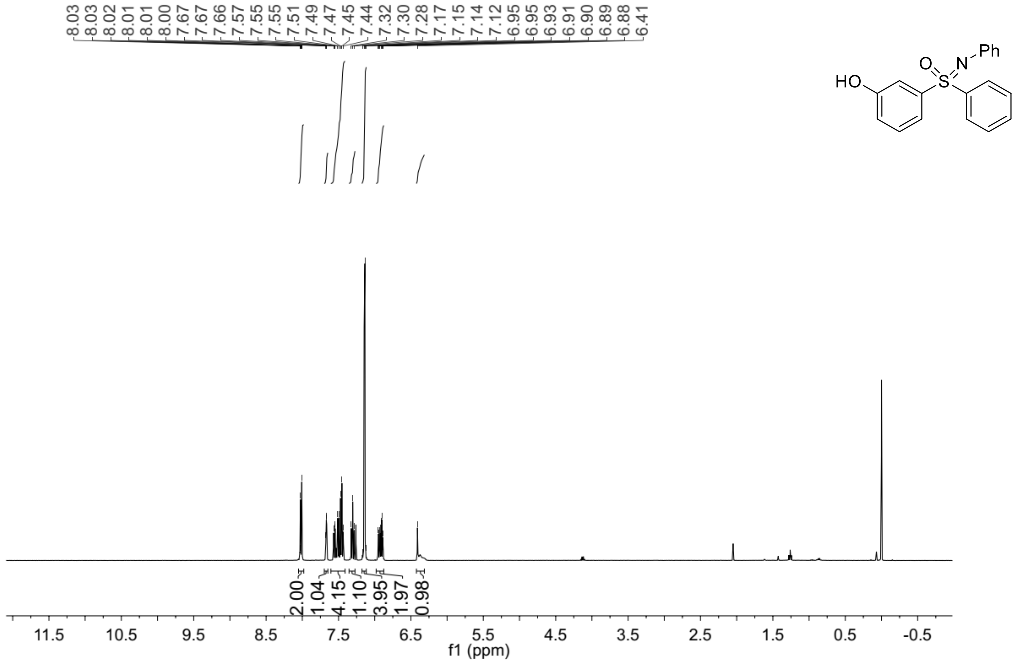

Supplementary Figure 70. ^{1}**H NMR spectra (CDCl$_3$, 600 MHz) of compound 3na**

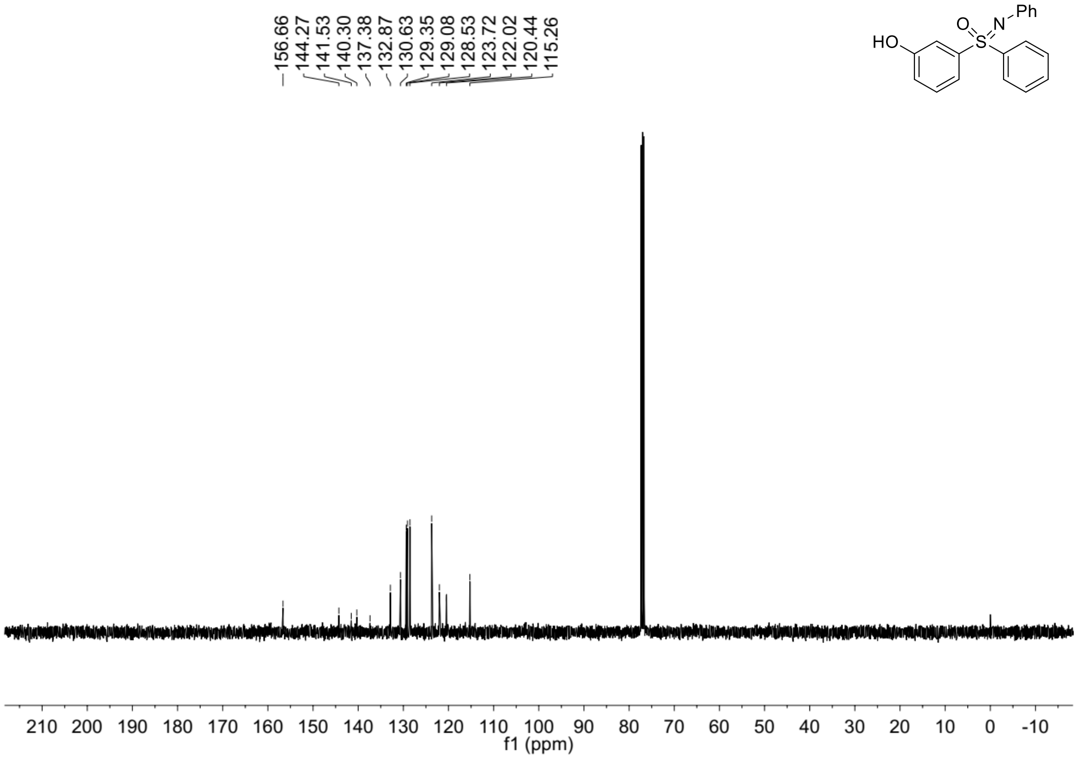

Supplementary Figure 71. ^{13}**C{**^{1}**H}NMR spectra (CDCl$_3$, 150 MHz) of compound 3na**

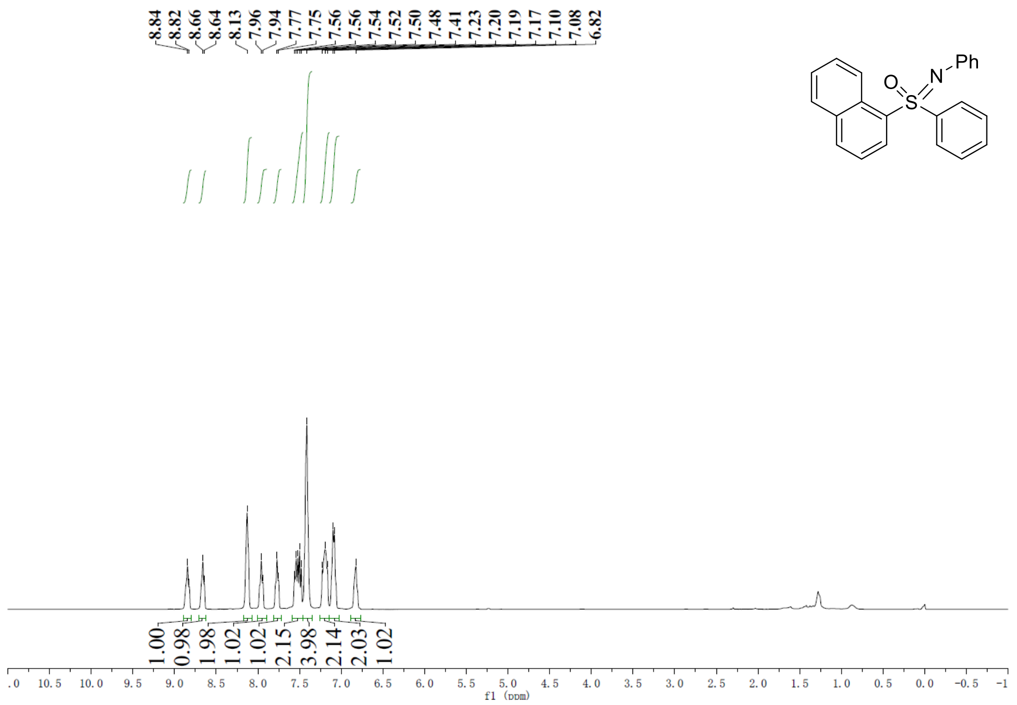

Supplementary Figure 72. ^{1}H NMR spectra ($CDCl_3$, 400 MHz) of compound 3oa

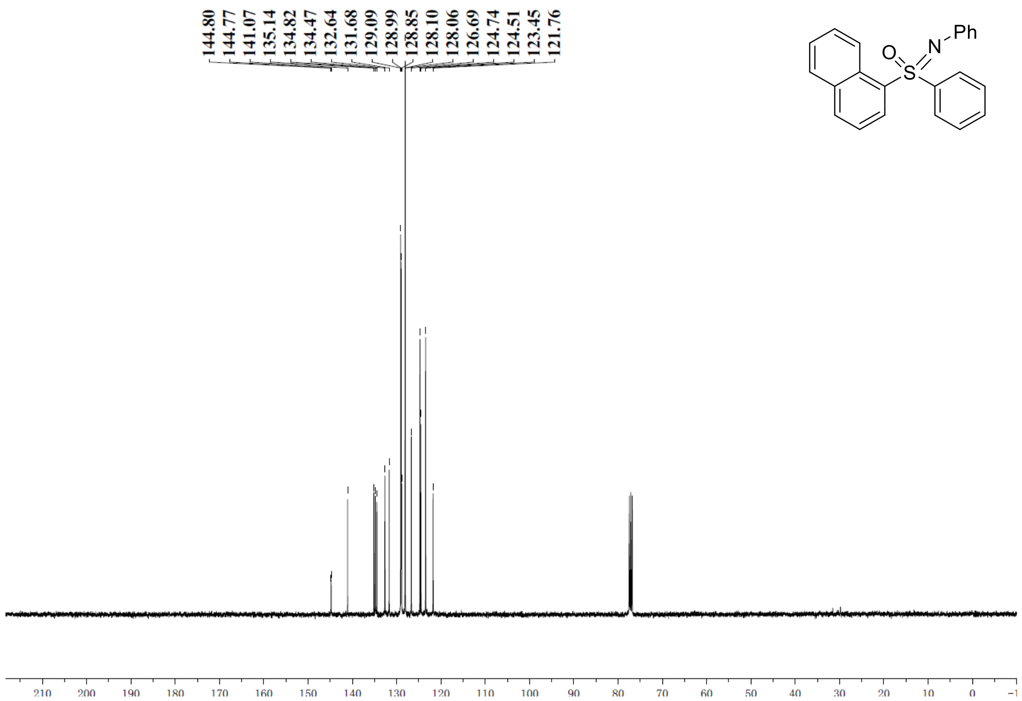

Supplementary Figure 73. $^{13}C\{^{1}H\}$NMR spectra ($CDCl_3$, 100 MHz) of compound 3oa

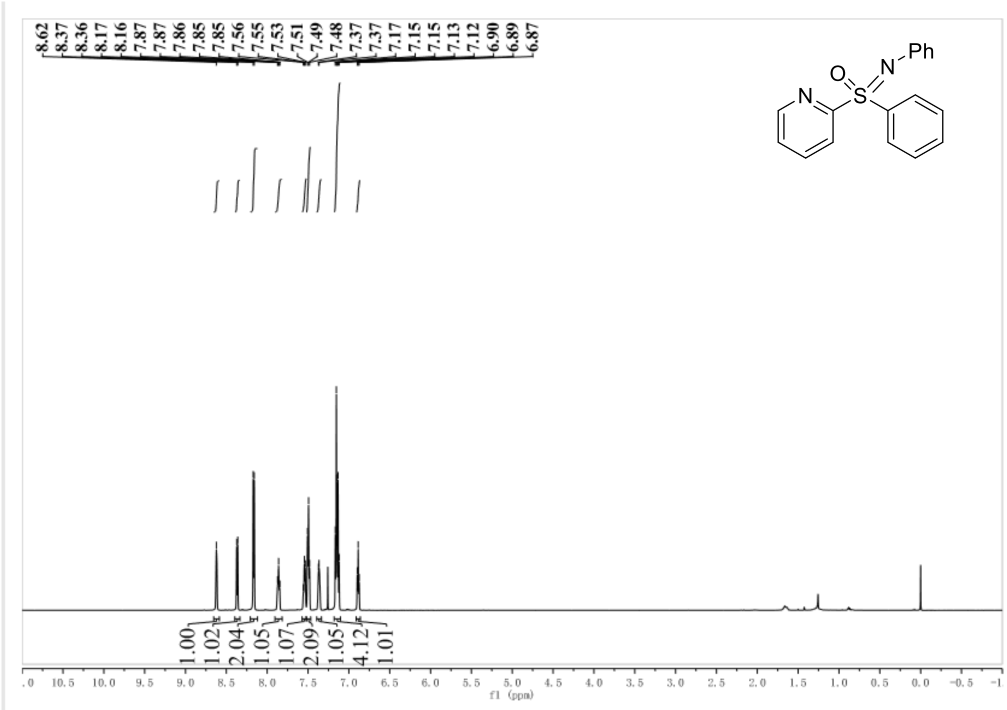

Supplementary Figure 74. ^{1}H NMR spectra ($CDCl_3$, 400 MHz) of compound 3pa

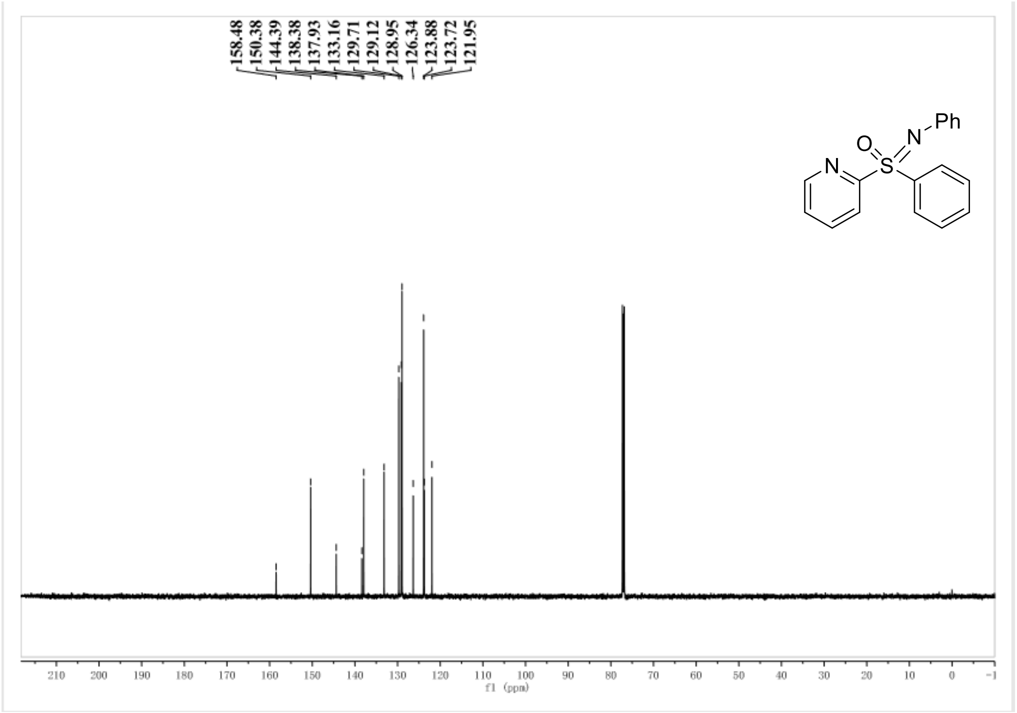

Supplementary Figure 75. $^{13}C\{^{1}H\}$NMR spectra ($CDCl_3$, 100 MHz) of compound 3pa

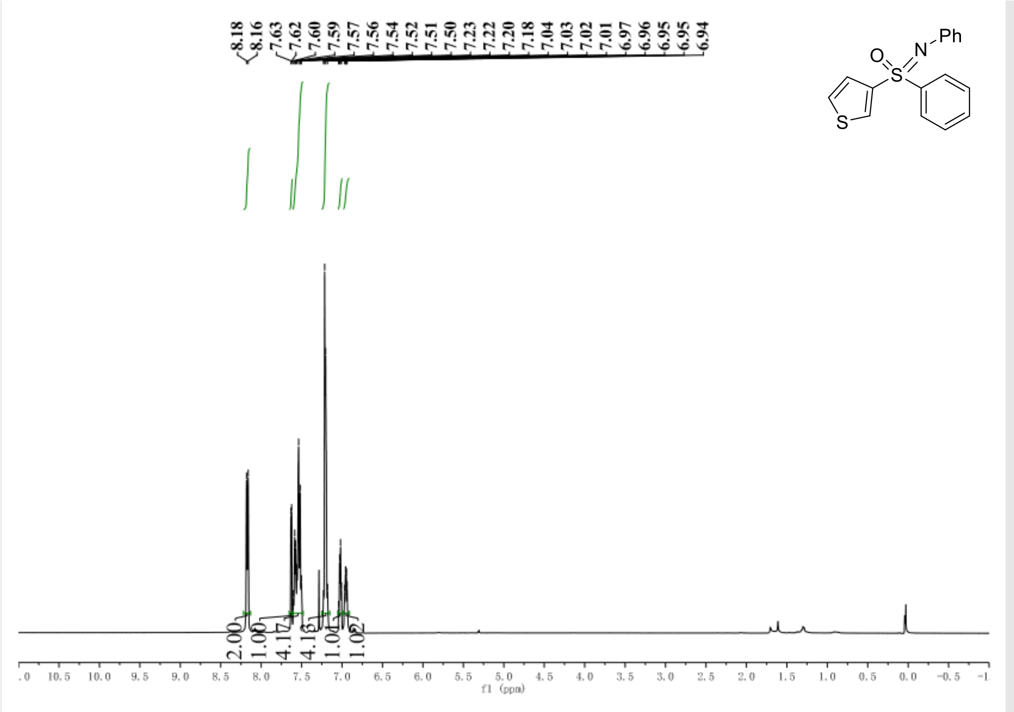

Supplementary Figure 76. ^{1}H NMR spectra ($CDCl_3$, 400 MHz) of compound 3qa

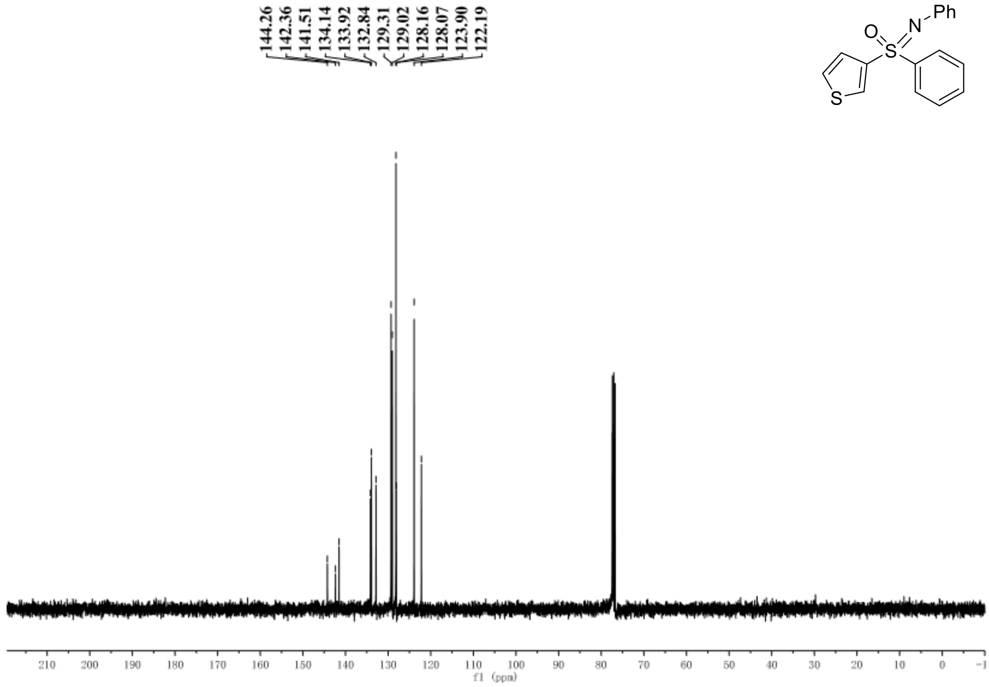

Supplementary Figure 77. $^{13}C\{^{1}H\}$NMR spectra ($CDCl_3$, 100 MHz) of compound 3qa

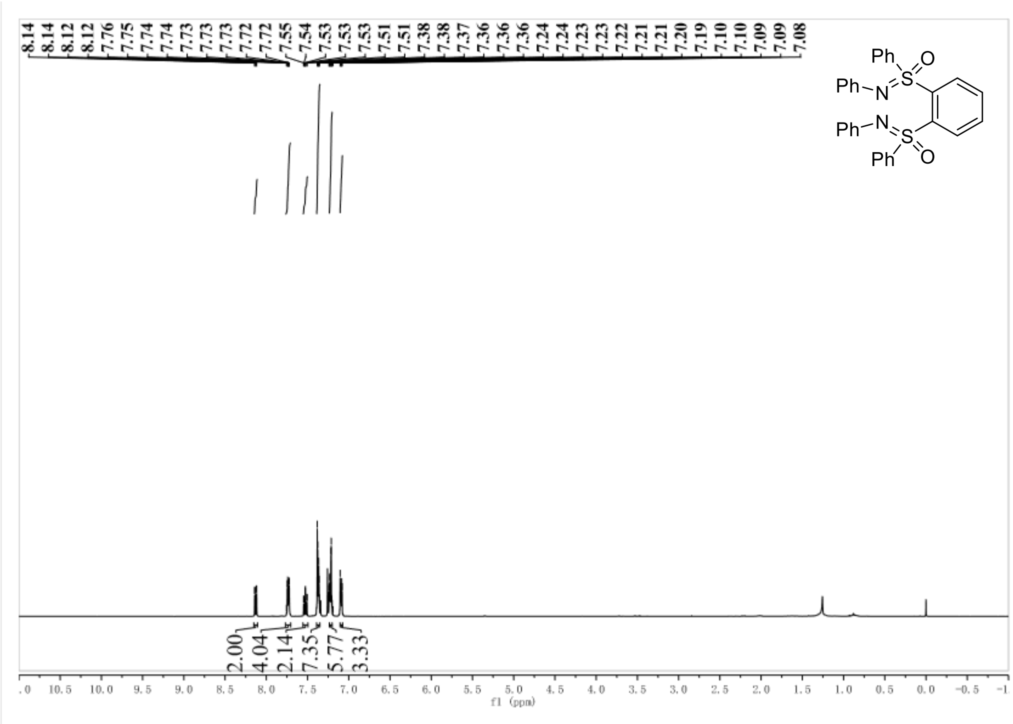

Supplementary Figure 78. ^{1}H NMR spectra ($CDCl_3$, 400 MHz) of compound 3ra

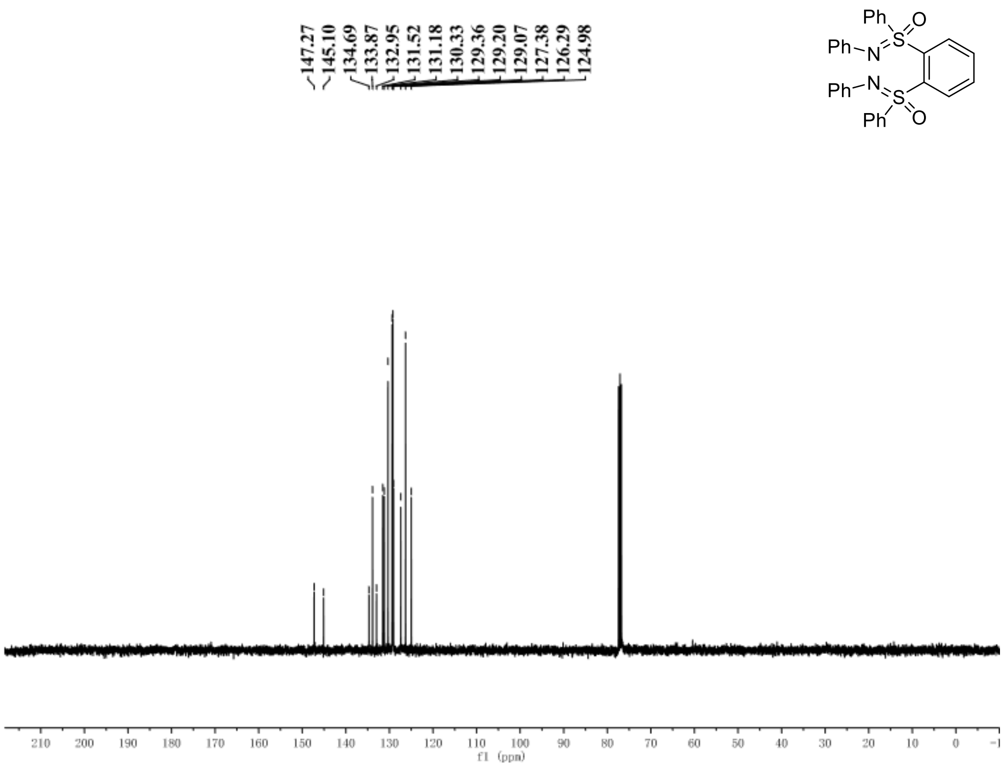

Supplementary Figure 79. $^{13}C\{^1H\}$NMR spectra ($CDCl_3$, 100 MHz) of compound 3ra

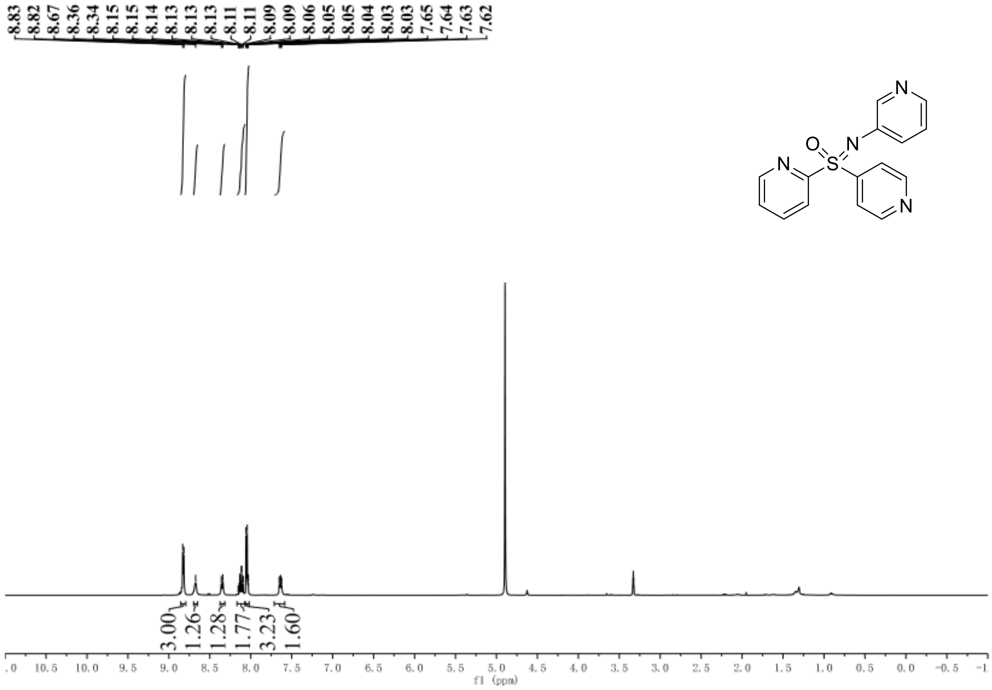

Supplementary Figure 80. ^{1}H NMR spectra ($CDCl_3$, 400 MHz) of compound 3sm

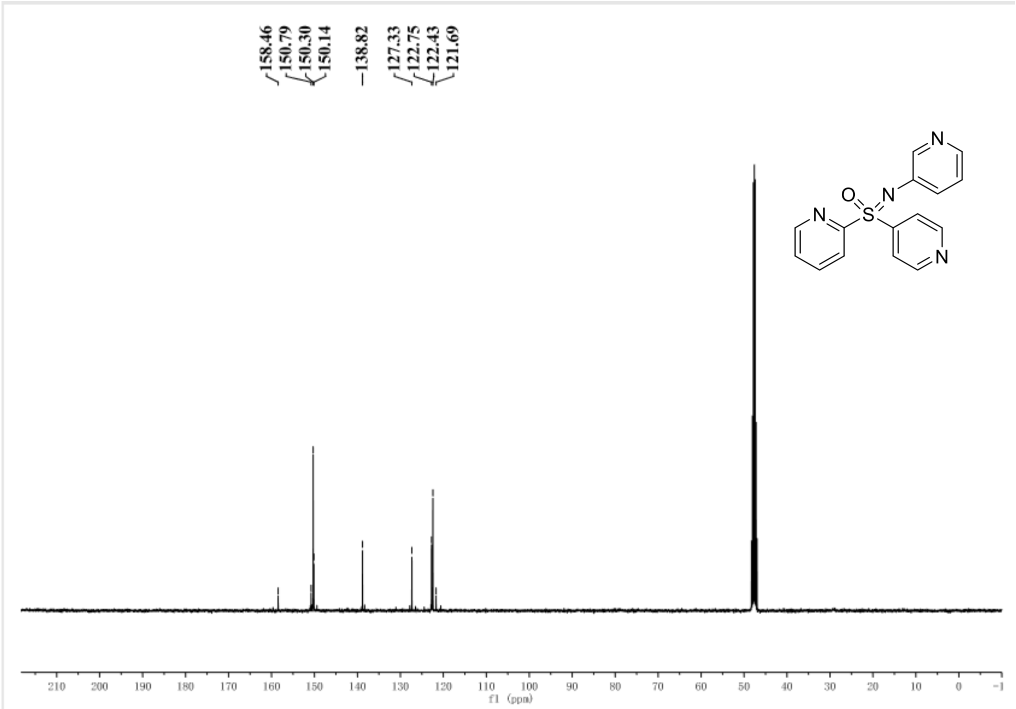

Supplementary Figure 81. $^{13}C\{^{1}H\}$NMR spectra ($CDCl_3$, 100 MHz) of compound 3sm

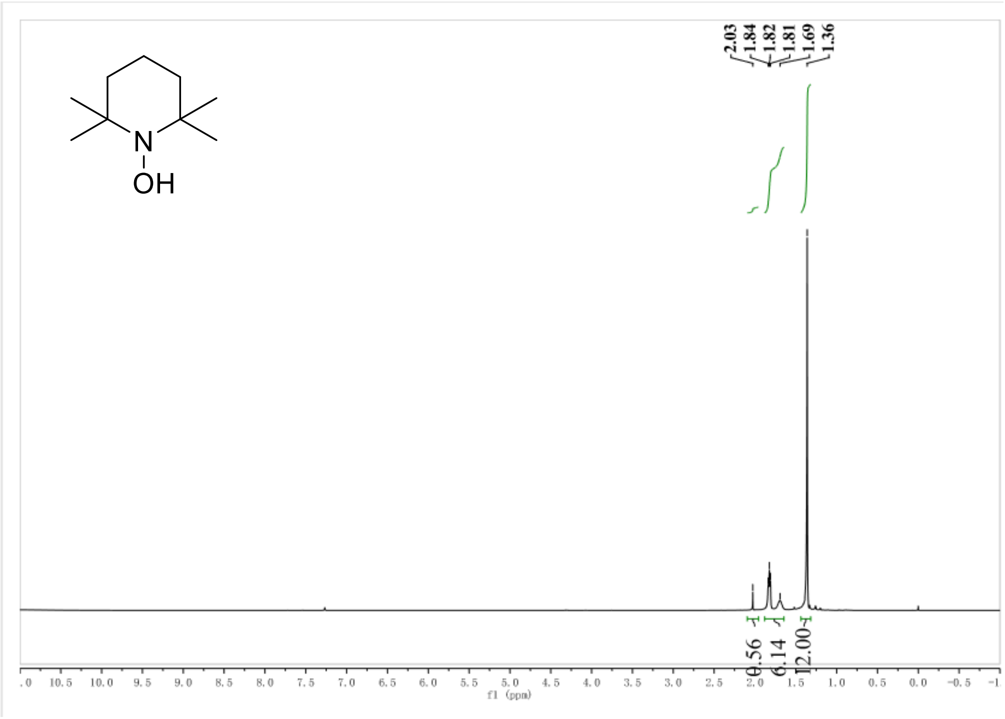

Supplementary Figure 82. ^{1}**H NMR spectra (CDCl$_3$, 400 MHz) of compound 4**

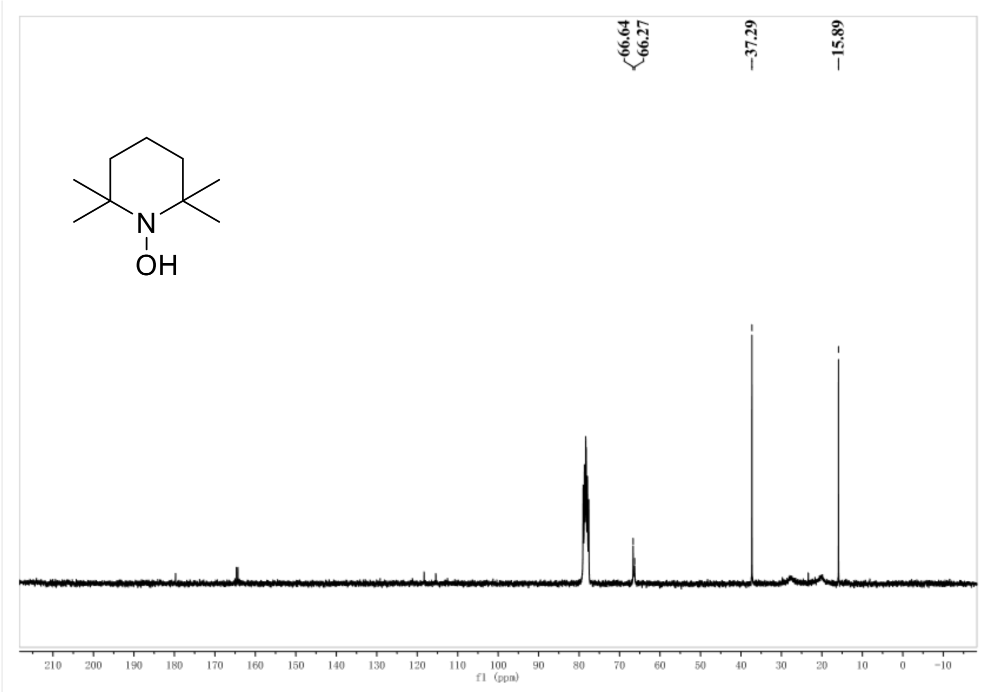

Supplementary Figure 83. ^{13}**C{**^{1}**H}NMR spectra (CDCl$_3$, 100 MHz) of compound 4**

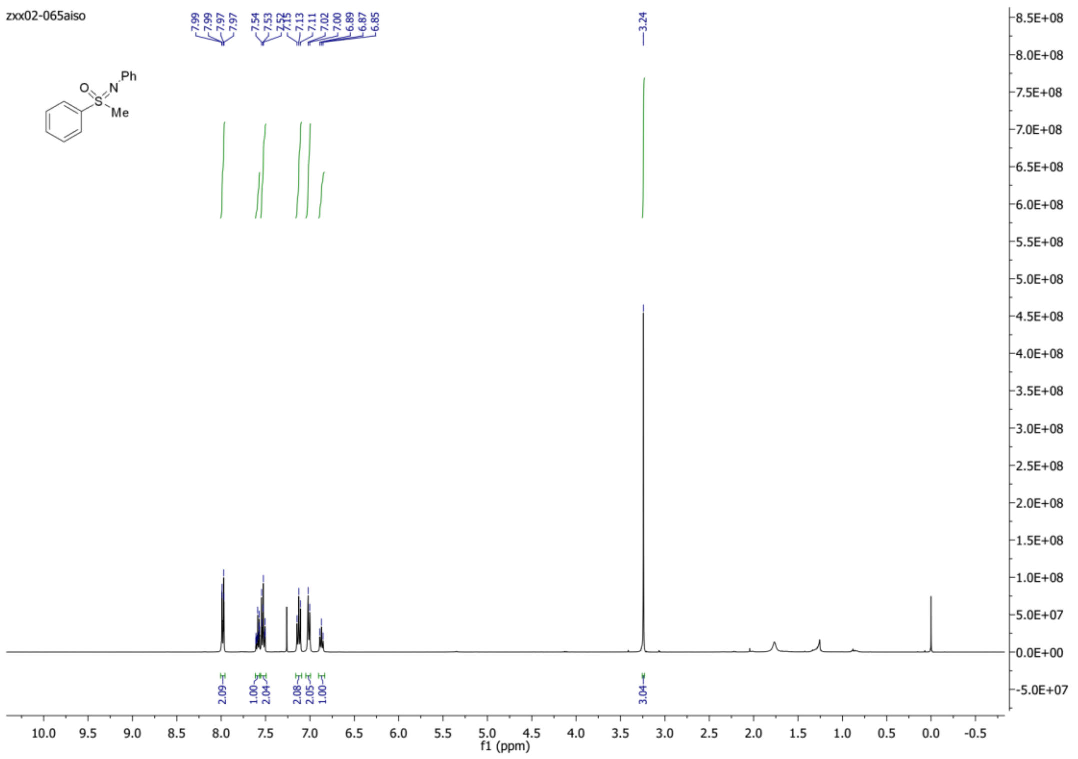

Supplementary Figure 84. ^{1}**H NMR spectra (CDCl$_3$, 400 MHz) of compound 7**

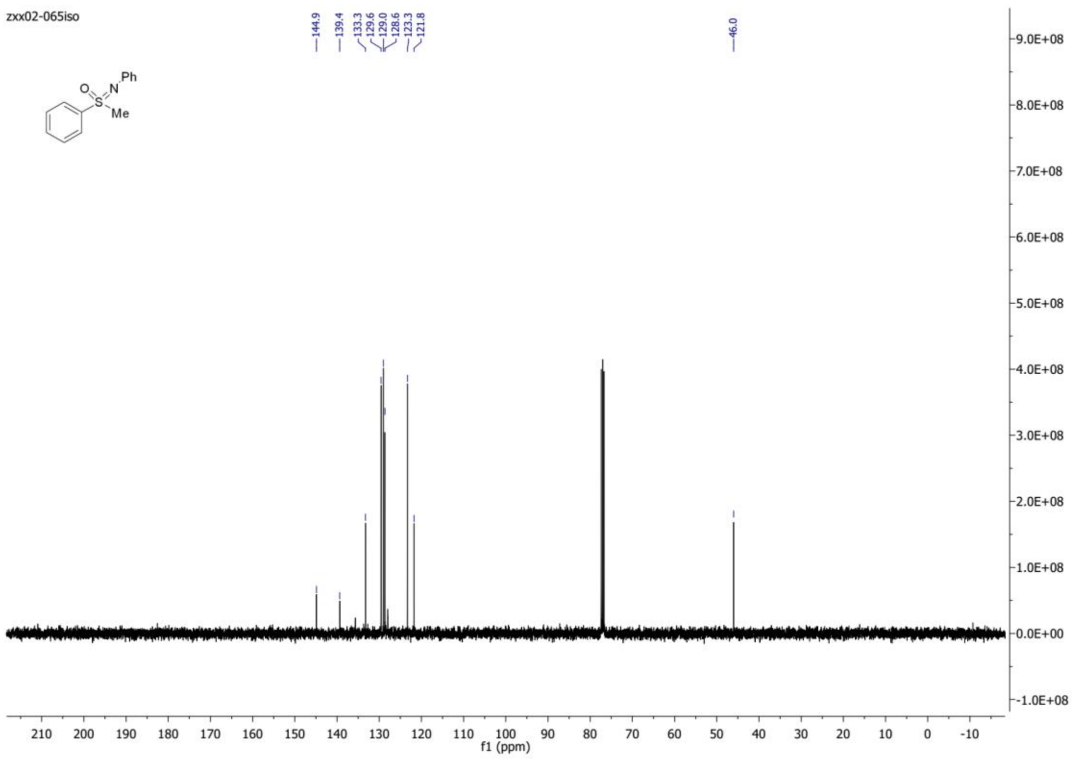

Supplementary Figure 85. ^{13}**C{^{1}H}NMR spectra (CDCl$_3$, 100 MHz) of compound 7**

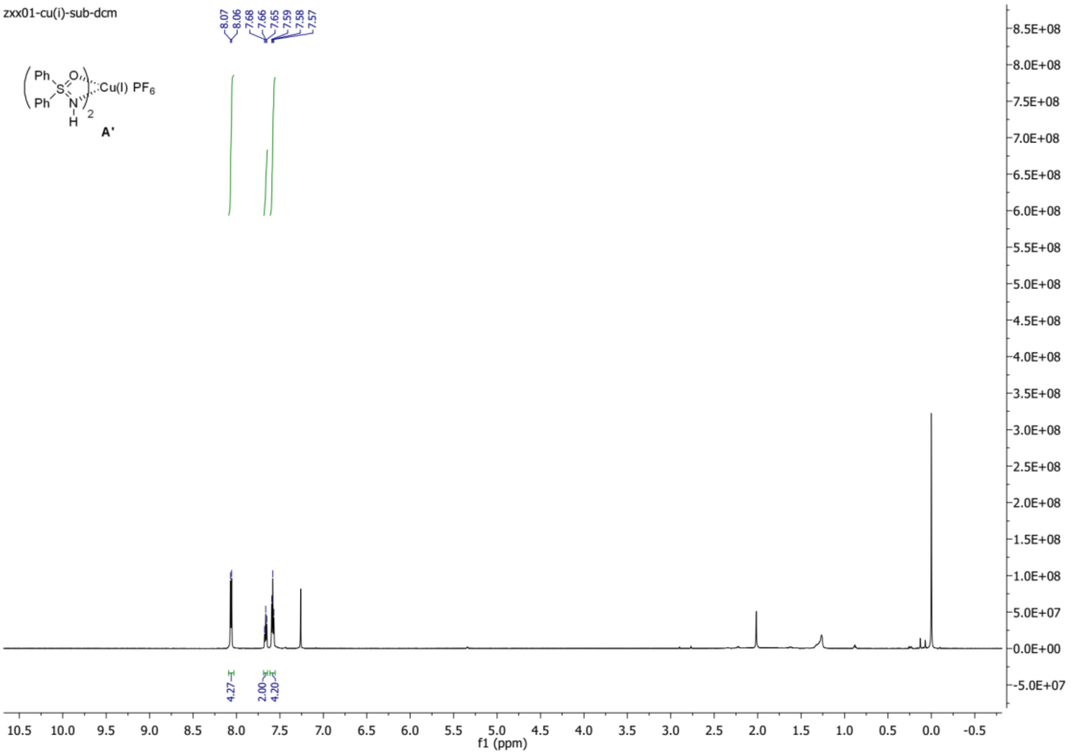

Supplementary Figure 86. ^{1}**H NMR spectra (CDCl$_3$, 600 MHz) of complex A'**

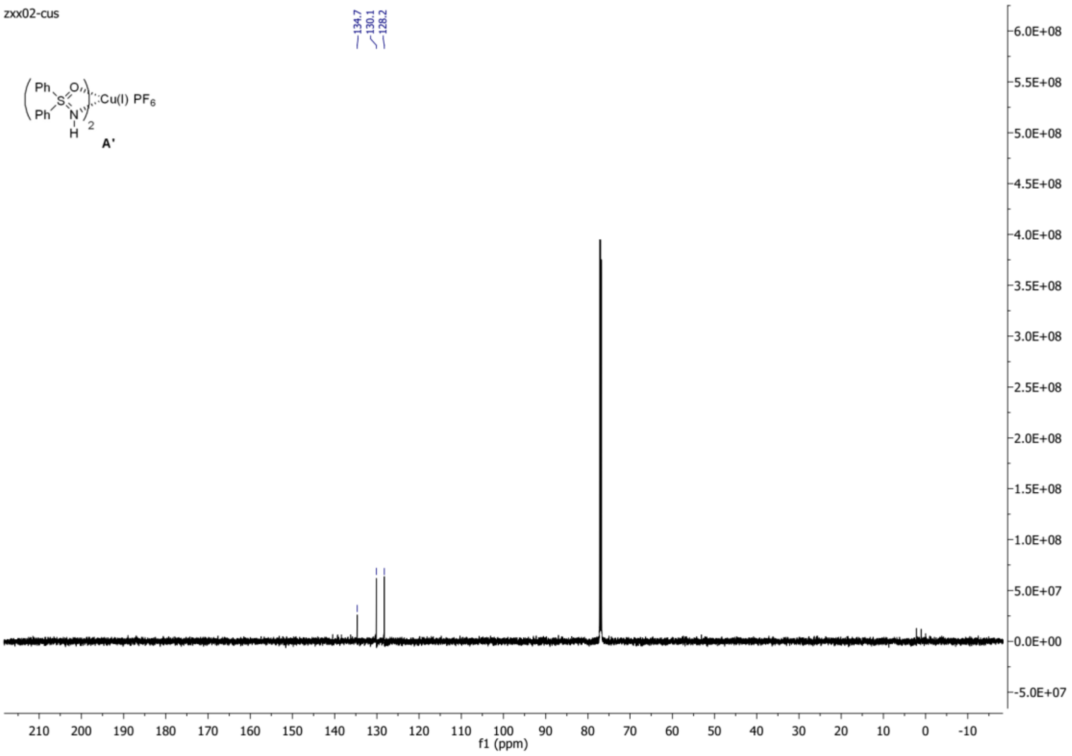

Supplementary Figure 87. ^{13}**C{^{1}H}NMR spectra (CDCl$_3$, 150 MHz) of complex A'**

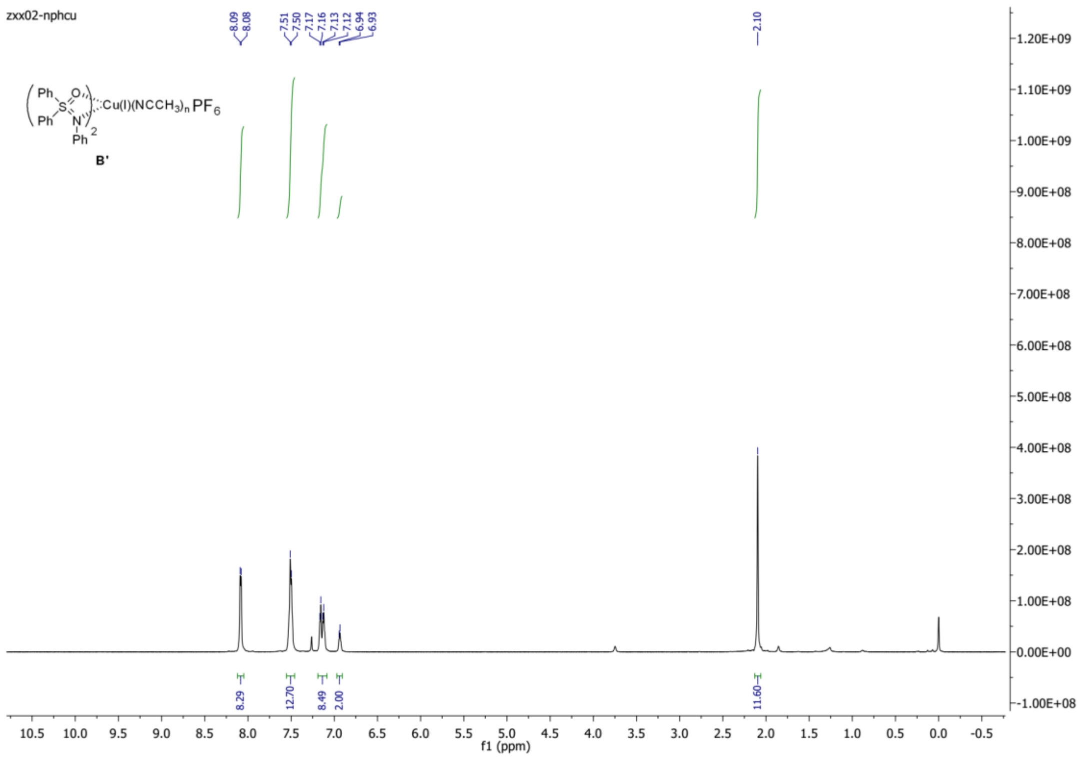

Supplementary Figure 88. ^{1}**H NMR spectra (CDCl$_3$, 600 MHz) of complex B'**

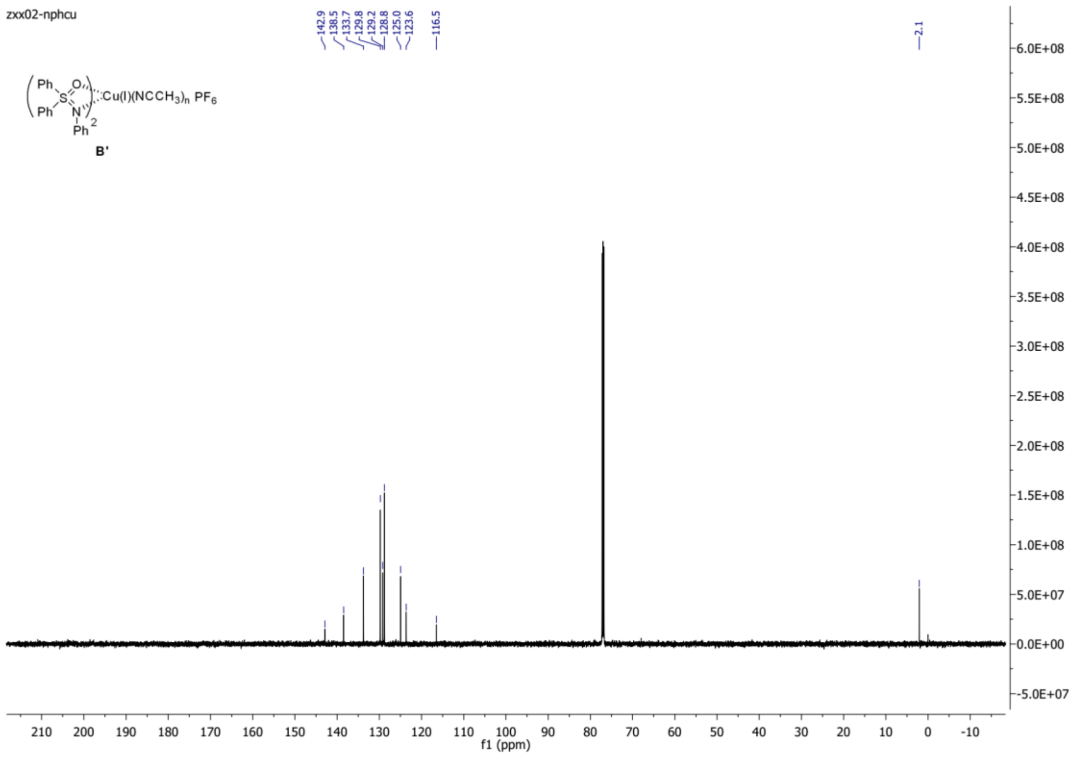

Supplementary Figure 89. ^{13}**C{**^{1}**H}NMR spectra (CDCl$_3$, 150 MHz) of complex B'**

Supplementary References

(1) Tota, A. et al. Synthesis of *NH*-sulfoximines from sulfides by chemoselective one-pot *N*- and *O*-transfers. *Chem. Comm.* **53**, 348-351 (2017).

(2) Bachon, A. K., Steinkamp, A. D. & Bolm, C. *N*-Arylated sulfoximines as cross-coupling building blocks. Adv. *Synth. Catal.* **360**, 1088-1093 (2018).

(3) Zhu, H., Teng, F., Pan, C. D., Cheng, J. & Yu, J. T. Radical *N*-arylation/alkylation of sulfoximines. *Tetrahedron Lett*. **57**, 2372-2374 (2016).

(4) Vaddula, B., Leazer, J. & Varma, R. S. Copper-catalyzed ultrasound-expedited *N*-arylation of sulfoximines using diaryliodonium salts. *Adv. Synth. Catal.* **354**, 986-990. (2012).

(5) Li, Y. J. et al. Copper(II)-catalyzed asymmetric photoredox reactions: enantioselective alkylation of imines driven by visible light. *J. Am. Chem. Soc*. **140**, 15850-15858 (2018).

(6) Liu, K. et al. Visible light-induced direct α C–H functionalization of alcohols. *Nat. Comm.* **10**, 467 (2019).

(7) Liu, Z. J., Vors, J. P., Gesing, E. R. F. & Bolm, C. Microwave-assisted solvent- and ligand-free copper-catalysed cross-coupling between halopyridines and nitrogen nucleophiles. *Green. Chem.* **13**, 42-45 (2011).

(8) Zhu, Y. M., Li, L. Y. & Shen, Z. M. Cu-catalyzed cyanation of arylboronic acids with acetonitrile: a dual role of TEMPO. *Chem. Eur. J.* **21**, 13246-13252 (2015).

(9) Frisch, M. J., et al. Gaussian 16, Revision B.01 (Gaussian, 2016).

(10) Becke, A. D. Density-functional thermochemistry. III. The role of exact exchange. *J. Chem. Phys.* **98,** 5648–5652 (1993).

(11) Becke, A. D. A new mixing of Hartree-Fock and local density-functional theories. *J. Chem. Phys.* **98,** 1372–1377 (1993).

(12) Lee, C., Yang, W. & Parr, R. G. Development of the Colle-Salvetti correlation-energy formula into a functional of the electron density. *Phys. Rev. B* **37**, 785-789 (1988)

(13) Legault, C. Y., *CYLview 1.0b*; (Université de Sherbrooke, 2009) http://www.cylview.org.
