# Supplementary material for: Autocatalytic photoredox Chan-Lam coupling of free diaryl sulfoximines with arylboronic acids
Source: Nat Commun. 2021 Feb 10;12:932. doi: 10.1038/s41467-021-21156-w (PMC7876119; doi:10.1038/s41467-021-21156-w)
Supplement: Supplementary file 3 — Description of Additional Supplementary Files [file 41467_2021_21156_MOESM3_ESM.pdf]

Description of Additional Supplementary Files

File Name: Supplementary Data 1
Description: According to the computational study, it showed the structures and energy difference of copper(I) and copper(II) photocatalysts by two transition states Pathways. Thermodynamic cycles for different copper (I) and copper (II) species were also showed in Supplementary Data 1

The structure of all possible transition states of copper species **I-XV**, substrate **1**, and coupling product **3** were listed in Supplementary Data 1. In part of structure of copper species, methyl groups were used in place of aryl groups and water was used in place of ethanol in order to reduce computation times.
