# Supplementary material for: Autocatalytic photoredox Chan-Lam coupling of free diaryl sulfoximines with arylboronic acids
Source: Nat Commun. 2021 Feb 10;12:932. doi: 10.1038/s41467-021-21156-w (PMC7876119; doi:10.1038/s41467-021-21156-w)
Supplement: Supplementary file 4 — Supplementary Data 1 [file 41467_2021_21156_MOESM4_ESM.zip › 268879_2_data_set_5233161_qmtd1l.docx]

**Coordinates and thermochemical data computed starting materials, intermediates, and transition states Pathways**

**I**

Zero-point correction= 0.249066 (Hartree/Particle)

Thermal correction to Energy= 0.269976

Thermal correction to Enthalpy= 0.270920

Thermal correction to Gibbs Free Energy= 0.198317

Sum of electronic and zero-point Energies= -1529.577711

Sum of electronic and thermal Energies= -1529.556802

Sum of electronic and thermal Enthalpies= -1529.555857

Sum of electronic and thermal Free Energies= -1529.628460

1 3

S 2.31737800 -0.62939100 -0.04323500

N 1.65533200 0.35084200 1.02736800

O 1.32597900 -1.52679700 -0.65899300

S -2.20173600 -0.47104700 -0.38428100

N -1.47357300 0.22713300 0.84293600

O -1.89132000 0.18766100 -1.67124400

H 2.35348300 0.86676700 1.56753100

C -1.83640400 -2.24017600 -0.52651200

H -2.14606400 -2.75608200 0.38584000

H -2.37623600 -2.62552300 -1.39500300

H -0.75543600 -2.31043000 -0.66957500

C -3.99303100 -0.41073100 -0.14073000

H -4.46685000 -0.89902600 -0.99577800

H -4.26890900 -0.90702100 0.79223800

H -4.25633800 0.64908300 -0.10746100

C 3.60881200 -1.58249800 0.79129900

H 4.06229800 -2.25962100 0.06270800

H 4.35839600 -0.90559400 1.20953100

H 3.11409500 -2.14698000 1.58469000

C 3.20063800 0.24586400 -1.35482300

H 3.64240000 -0.49301600 -2.02845600

H 2.44296900 0.84658800 -1.86590300

H 3.96871800 0.88523900 -0.91144700

C -1.61259000 -0.37887500 2.18248800

H -1.16793700 0.32369400 2.89128100

H -2.66234700 -0.51292800 2.47168000

H -1.08383800 -1.33550400 2.27019100

O 0.36789900 1.77803300 -1.66097000

H -0.42976700 1.28283300 -1.94881600

H 0.19015200 2.71455600 -1.85325800

Cu 0.05978000 1.58039600 0.63818800

**II**

Zero-point correction= 0.239492 (Hartree/Particle)

Thermal correction to Energy= 0.261121

Thermal correction to Enthalpy= 0.262065

Thermal correction to Gibbs Free Energy= 0.188058

Sum of electronic and zero-point Energies= -1529.560161

Sum of electronic and thermal Energies= -1529.538531

Sum of electronic and thermal Enthalpies= -1529.537587

Sum of electronic and thermal Free Energies= -1529.611594

1 3

S 2.17247300 -0.80741400 0.13006100

N 1.74433700 0.70337300 0.34459200

O 1.26470400 -1.76462600 0.78292800

S -1.98792700 -0.70320400 -0.33108000

N -1.56441800 0.48321700 0.64112800

O -1.13813100 -0.71357200 -1.53738300

H 2.46944000 1.39000200 0.10900700

C -1.92976600 -2.30442300 0.50413600

H -2.57126300 -2.28748600 1.38869700

H -2.26694000 -3.06473700 -0.20464800

H -0.88229600 -2.45657800 0.77649200

C -3.71873200 -0.51580000 -0.81360800

H -3.99630400 -1.36112100 -1.44795300

H -4.35231500 -0.47185300 0.07582700

H -3.77654200 0.42080800 -1.37342600

C 3.85359000 -0.97177600 0.76430600

H 4.17178700 -2.00780400 0.62531400

H 4.51902900 -0.28472200 0.23498000

H 3.80679500 -0.72327600 1.82708100

C 2.30345900 -1.24786100 -1.61952800

H 2.67013600 -2.27447800 -1.69893100

H 1.28878500 -1.16930900 -2.01922500

H 2.97499800 -0.54737100 -2.12254400

C -2.13668700 0.56679900 2.00057900

H -1.83033600 1.52602500 2.42175300

H -3.23373900 0.55268900 1.99286600

H -1.76550300 -0.22900900 2.65717900

O 0.56216900 3.13224700 -0.54792500

H 0.55098800 3.22599300 -1.51646900

H 2.67829700 3.27518900 -0.57203800

Cu -0.01407900 1.51575000 0.01951800

**III**

Zero-point correction= 0.237551 (Hartree/Particle)

Thermal correction to Energy= 0.257969

Thermal correction to Enthalpy= 0.258913

Thermal correction to Gibbs Free Energy= 0.187347

Sum of electronic and zero-point Energies= -1529.547431

Sum of electronic and thermal Energies= -1529.527012

Sum of electronic and thermal Enthalpies= -1529.526068

Sum of electronic and thermal Free Energies= -1529.597634

1 3

S 2.18243600 -0.78443600 0.09613900

N 1.71952500 0.64304400 0.62435500

O 1.28191800 -1.91570200 0.42249900

S -2.07066500 -0.59800100 -0.37334400

N -1.57199100 0.45044700 0.72745000

O -1.31287800 -0.41043200 -1.62484800

H 2.45308200 1.71759700 0.04715000

C -1.93006200 -2.29841300 0.21521300

H -2.50227800 -2.42182100 1.13813000

H -2.31213000 -2.95558400 -0.56999300

H -0.86169800 -2.46337000 0.38286300

C -3.83417500 -0.36230800 -0.67795600

H -4.16002200 -1.10636400 -1.40900500

H -4.39235900 -0.46386000 0.25614500

H -3.93956000 0.64703800 -1.08301900

C 3.80582000 -1.07672300 0.82361100

H 4.16747100 -2.05181600 0.48870300

H 4.48536600 -0.27434100 0.52704000

H 3.65928200 -1.06992900 1.90593700

C 2.48671900 -0.79900900 -1.69107900

H 2.91767200 -1.76603200 -1.96251100

H 1.50979500 -0.67394800 -2.16550800

H 3.15785700 0.02182400 -1.95557700

C -2.03566700 0.33903800 2.12515900

H -1.67501000 1.22203500 2.65529900

H -3.13003000 0.33757300 2.19963000

H -1.63241500 -0.54874300 2.62645300

O 0.69311800 2.96356800 -0.60362000

H 0.63632000 2.98920900 -1.57648500

H 2.36120600 2.50761600 -0.36983300

Cu 0.00632100 1.41043100 0.12121700

**IV**

Zero-point correction= 0.239718 (Hartree/Particle)

Thermal correction to Energy= 0.261938

Thermal correction to Enthalpy= 0.262882

Thermal correction to Gibbs Free Energy= 0.186301

Sum of electronic and zero-point Energies= -1529.560999

Sum of electronic and thermal Energies= -1529.538779

Sum of electronic and thermal Enthalpies= -1529.537835

Sum of electronic and thermal Free Energies= -1529.614416

1 3

S 2.54545500 -0.45370400 -0.18944300

N 1.89410900 0.51727900 0.85768100

O 1.72969300 -1.10773500 -1.24957300

S -2.31461900 -0.40390800 -0.43257900

N -1.52925000 -0.06149500 0.90086500

O -2.29063400 0.73742400 -1.37845600

C -1.65386500 -1.85735300 -1.28295400

H -1.74373100 -2.73061400 -0.63094800

H -2.21434500 -2.00337600 -2.20958900

H -0.59969000 -1.64025000 -1.48739800

C -4.02806100 -0.84107500 -0.06175100

H -4.53091300 -1.02504100 -1.01415800

H -4.07195900 -1.72693700 0.57536300

H -4.46749000 0.02374500 0.44129500

C 3.39069100 -1.76054600 0.74088100

H 3.91018300 -2.41288300 0.03531200

H 4.07815000 -1.30557900 1.45590500

H 2.61213500 -2.31702200 1.26753200

C 3.90650100 0.45660600 -0.96868900

H 4.44575700 -0.23161000 -1.62398600

H 3.44780900 1.25499300 -1.55589000

H 4.55522100 0.87195600 -0.19575600

C -1.59675400 -1.00104500 2.04144500

H -1.04752200 -0.54196300 2.86476700

H -2.62628000 -1.15694100 2.38404000

H -1.13437100 -1.97362700 1.82684100

Cu 0.16845800 1.00138900 0.59787500

H 0.85709700 4.69737900 -1.66791600

H -0.28282800 3.28543900 -0.59492500

O -0.80595800 2.64157800 -0.08409100

H -1.49577500 2.27368000 -0.68298700

**V**

Zero-point correction= 0.236141 (Hartree/Particle)

Thermal correction to Energy= 0.257056

Thermal correction to Enthalpy= 0.258000

Thermal correction to Gibbs Free Energy= 0.184833

Sum of electronic and zero-point Energies= -1529.545345

Sum of electronic and thermal Energies= -1529.524430

Sum of electronic and thermal Enthalpies= -1529.523485

Sum of electronic and thermal Free Energies= -1529.596653

1 3

S 2.53729500 -0.45775900 -0.17821800

N 1.87825900 0.60140000 0.78227000

O 1.71677000 -1.24419300 -1.13661400

S -2.28423300 -0.47111000 -0.40413800

N -1.47011400 -0.05961300 0.89157800

O -2.27343500 0.61671000 -1.41036300

C -1.64638000 -1.97519600 -1.18053500

H -1.73481100 -2.81111100 -0.48121500

H -2.22103200 -2.16573300 -2.09021500

H -0.59371700 -1.77822300 -1.40927800

C -3.99267800 -0.87640000 0.02340400

H -4.51306700 -1.12866700 -0.90363900

H -4.02659600 -1.71260800 0.72513000

H -4.42114700 0.02426600 0.46982000

C 3.45030800 -1.62595600 0.86485100

H 3.97543200 -2.33222500 0.21771400

H 4.13775800 -1.07269000 1.50679300

H 2.70546700 -2.14864100 1.46915200

C 3.83975300 0.42300000 -1.08169600

H 4.38662600 -0.30845200 -1.68174300

H 3.33452000 1.14214100 -1.73003200

H 4.49182200 0.93500900 -0.37222600

C -1.46917300 -0.95532200 2.06677600

H -0.91760900 -0.44418400 2.85737600

H -2.48183400 -1.13938000 2.44466000

H -0.97375300 -1.91617300 1.87412500

Cu 0.15393100 1.10983100 0.50479000

H 0.04821500 4.05130500 -1.39310700

H -0.32694200 3.49968300 -0.80779700

O -0.91612100 2.62969600 -0.09585700

H -1.54802400 2.23152700 -0.74779800

**VI**

Zero-point correction= 0.224797 (Hartree/Particle)

Thermal correction to Energy= 0.242816

Thermal correction to Enthalpy= 0.243760

Thermal correction to Gibbs Free Energy= 0.176979

Sum of electronic and zero-point Energies= -1453.175612

Sum of electronic and thermal Energies= -1453.157593

Sum of electronic and thermal Enthalpies= -1453.156649

Sum of electronic and thermal Free Energies= -1453.223429

1 3

S -1.93496400 -0.74550100 -0.16755100

N -1.62949800 0.79707000 0.10701300

O -1.09520600 -1.29972900 -1.24751100

S 2.25835500 -0.48159300 0.16034400

N 1.32293800 0.24468800 -0.91764600

O 1.64835100 -1.16589800 1.32150200

Cu 0.09918400 1.74955200 -0.35609200

C 3.29905000 -1.65118600 -0.74357400

H 3.79732800 -1.13359900 -1.56519200

H 4.01608400 -2.07974800 -0.03881100

H 2.63632300 -2.43371500 -1.12258700

C 3.37293900 0.81997300 0.70457700

H 4.05778500 0.38145500 1.43385300

H 3.90229600 1.22933900 -0.15763800

H 2.75847600 1.59120900 1.17655000

C -3.67721900 -0.95216100 -0.60997700

H -3.83439600 -2.01050700 -0.83237500

H -4.32416800 -0.62622700 0.20714700

H -3.84033200 -0.34540300 -1.50400800

C -1.72361700 -1.75843900 1.31765400

H -2.01692000 -2.78361600 1.07770600

H -0.65869200 -1.70652900 1.56390600

H -2.33395400 -1.36039400 2.13203100

C -2.47073100 1.51764200 1.08746900

H -2.08380200 2.53962100 1.13819700

H -3.51507300 1.58027600 0.76009600

H -2.42246300 1.09015000 2.09664800

H 0.72915900 -0.45810400 -1.37792000

**VII**

Zero-point correction= 0.216635 (Hartree/Particle)

Thermal correction to Energy= 0.234747

Thermal correction to Enthalpy= 0.235691

Thermal correction to Gibbs Free Energy= 0.169028

Sum of electronic and zero-point Energies= -1453.131859

Sum of electronic and thermal Energies= -1453.113746

Sum of electronic and thermal Enthalpies= -1453.112802

Sum of electronic and thermal Free Energies= -1453.179465

1 3

S 2.31888500 -0.39337600 0.19545800

N 1.69430800 1.09224000 0.31823400

O 1.58309500 -1.46111800 0.91599900

S -1.95283600 -0.66919100 -0.20291600

N -1.66843400 0.82961800 0.30909000

O -1.13213200 -0.96956000 -1.38746100

C -1.66674200 -1.89426300 1.09379700

H -2.25076700 -1.64352700 1.98279500

H -1.95899600 -2.87051700 0.69828000

H -0.59123100 -1.86502700 1.29556700

C -3.70657600 -0.80828500 -0.60152300

H -3.89877600 -1.83188900 -0.93264300

H -4.31330200 -0.56175000 0.27317000

H -3.88869000 -0.09967200 -1.41310500

C 4.01470300 -0.27202900 0.79962500

H 4.49406500 -1.24832400 0.68862600

H 4.54101800 0.49926400 0.23222300

H 3.94645000 0.00924600 1.85271300

C 2.50758400 -0.89482300 -1.53594800

H 3.04670400 -1.84540700 -1.55548700

H 1.49695000 -1.02217500 -1.93142000

H 3.04690500 -0.11959400 -2.08551700

C -2.22868100 1.28784300 1.59747900

H -2.03525600 2.35982300 1.66841000

H -3.31624500 1.15128600 1.63747700

H -1.76555000 0.79503400 2.46027900

Cu -0.04528100 1.54875500 -0.43586700

H 1.59253200 1.86216800 -0.85203800

**VIII**

Zero-point correction= 0.223954 (Hartree/Particle)

Thermal correction to Energy= 0.242301

Thermal correction to Enthalpy= 0.243245

Thermal correction to Gibbs Free Energy= 0.176050

Sum of electronic and zero-point Energies= -1453.171094

Sum of electronic and thermal Energies= -1453.152747

Sum of electronic and thermal Enthalpies= -1453.151803

Sum of electronic and thermal Free Energies= -1453.218997

1 3

S 2.17482700 -0.45149300 0.11515400

N 1.71507200 1.07277700 0.00867100

O 1.29760200 -1.25129700 0.98605800

S -1.85848500 -0.66806400 -0.23355100

N -1.41589800 0.65888300 0.53461600

O -1.07875800 -0.86544500 -1.46955500

C -1.77540400 -2.13696600 0.82294600

H -2.40551200 -2.00145300 1.70512000

H -2.12105300 -2.98439100 0.22543200

H -0.72504700 -2.25139400 1.09827800

C -3.60837700 -0.52856700 -0.65508400

H -3.92882900 -1.45172500 -1.14465000

H -4.19053900 -0.33997700 0.25064600

H -3.68309500 0.31894000 -1.34090800

C 3.87594100 -0.47491700 0.72829300

H 4.19580900 -1.51709600 0.80756800

H 4.52595000 0.07956800 0.04633300

H 3.85478000 -0.00433800 1.71395800

C 2.27020800 -1.25285700 -1.50242200

H 2.64400100 -2.27140200 -1.36883100

H 1.24645800 -1.26226700 -1.88600700

H 2.92598800 -0.67373200 -2.15746400

C -1.66846300 0.80637100 1.97983200

H -1.39845300 1.83152200 2.24548000

H -2.73014200 0.67855000 2.22742400

H -1.06191700 0.11977100 2.58083600

Cu -0.10581800 1.87223700 -0.37689500

H 2.45046000 1.68140000 -0.35531800

**Thermodynamic Cycles**

**IX**

Zero-point correction= 0.410529 (Hartree/Particle)

Thermal correction to Energy= 0.439267

Thermal correction to Enthalpy= 0.440211

Thermal correction to Gibbs Free Energy= 0.344185

Sum of electronic and zero-point Energies= -2180.742582

Sum of electronic and thermal Energies= -2180.713843

Sum of electronic and thermal Enthalpies= -2180.712899

Sum of electronic and thermal Free Energies= -2180.808926

1 1

S -2.79828900 -0.23403900 0.31581400

N -1.76289100 -0.36422500 -0.90080100

O -2.53610400 -0.87602300 1.62014700

S 3.02247900 -0.24084700 -0.85152100

N 1.82495700 0.77279600 -0.51629200

O 3.15803700 -0.84606500 -2.19160600

H 2.03994800 1.68214900 -0.93149400

H -1.88347300 -1.27804400 -1.34327400

Cu 0.03331800 0.19077900 -0.69011300

C -2.93127100 1.53500400 0.56598500

C -3.13275300 1.97302100 1.87738100

C -2.87838000 2.42276800 -0.51434300

C -3.27537100 3.34157200 2.10963800

H -3.16004300 1.25676200 2.69120800

C -3.02314700 3.78651200 -0.26231300

H -2.71470500 2.05710000 -1.52167500

C -3.22246300 4.24341900 1.04430400

H -3.42678800 3.70041000 3.12292500

H -2.98116700 4.49147100 -1.08696400

H -3.33725100 5.30696200 1.23131600

C -4.36351900 -0.85186100 -0.31254700

C -5.17457200 -1.56206600 0.57635700

C -4.74432800 -0.61306900 -1.63803400

C -6.39861100 -2.05014300 0.11754500

H -4.84149900 -1.73838400 1.59314400

C -5.97093200 -1.10861400 -2.07769300

H -4.09632100 -0.06409700 -2.31281700

C -6.79510200 -1.82265700 -1.20230900

H -7.03866000 -2.61053200 0.79184600

H -6.28151100 -0.93828200 -3.10388300

H -7.74916600 -2.20494100 -1.55264200

C 4.53409400 0.65856600 -0.48694200

C 5.63044800 0.43429500 -1.32359500

C 4.59701700 1.53483200 0.60246000

C 6.82001100 1.11468100 -1.06052400

H 5.54130700 -0.24309400 -2.16567700

C 5.79430600 2.20468700 0.84957500

H 3.73084800 1.69798700 1.23469700

C 6.90155900 1.99361900 0.02190300

H 7.67997600 0.95874000 -1.70434600

H 5.86175000 2.89211400 1.68714200

H 7.83064200 2.51912300 0.22141100

C 2.81619000 -1.54963900 0.35329900

C 2.36112300 -1.27789400 1.64829400

C 3.16665500 -2.83905600 -0.05584000

C 2.24724800 -2.33646800 2.54836900

H 2.08698900 -0.27003500 1.93810600

C 3.04699700 -3.88496500 0.85993900

H 3.50623700 -3.01418200 -1.07079500

C 2.59050700 -3.63403400 2.15594400

H 1.88747900 -2.14730100 3.55496900

H 3.30813400 -4.89431900 0.55762000

H 2.49984000 -4.45236700 2.86399000

**X**

Zero-point correction= 0.490928 (Hartree/Particle)

Thermal correction to Energy= 0.524585

Thermal correction to Enthalpy= 0.525529

Thermal correction to Gibbs Free Energy= 0.417199

Sum of electronic and zero-point Energies= -2411.702837

Sum of electronic and thermal Energies= -2411.669180

Sum of electronic and thermal Enthalpies= -2411.668236

Sum of electronic and thermal Free Energies= -2411.776566

1 1

S -3.19298800 -0.11793200 -0.69698600

N -2.03913700 0.91955500 -0.29281600

O -3.21102000 -0.76473600 -2.02449800

S 2.59968700 0.52603900 0.70409900

N 1.58865800 -0.21959900 -0.29714600

O 2.50781500 0.32298600 2.16725100

H -2.22591400 1.81322800 -0.75308500

Cu -0.22992100 0.35965400 -0.28936300

C -3.07911000 -1.38529800 0.56279700

C -3.37731700 -2.69182600 0.16774600

C -2.74544700 -1.06478800 1.88342700

C -3.33050300 -3.70520600 1.12573100

H -3.62063600 -2.90471800 -0.86737500

C -2.70366300 -2.09126400 2.82587000

H -2.50883800 -0.04427700 2.16198200

C -2.99699800 -3.40550300 2.44846100

H -3.55194600 -4.72746800 0.83542600

H -2.44021200 -1.86390200 3.85423900

H -2.96373000 -4.19860700 3.18951600

C -4.73954300 0.77317200 -0.48885000

C -5.76076200 0.50706000 -1.40455000

C -4.90206300 1.68357500 0.56152400

C -6.97549800 1.17908900 -1.26303000

H -5.59497900 -0.19622500 -2.21313200

C -6.12300300 2.34462500 0.68699200

H -4.09300800 1.87910100 1.25710100

C -7.15591800 2.09151500 -0.22103200

H -7.77774900 0.99020400 -1.96951500

H -6.26688300 3.05772300 1.49288100

H -8.10418300 2.61009600 -0.11595400

C 4.28178900 0.13719100 0.19527900

C 5.23495800 -0.02309900 1.20343300

C 4.61138500 0.04124800 -1.16050900

C 6.55566900 -0.28675400 0.83753100

H 4.94091700 0.04789500 2.24462300

C 5.93507700 -0.22316200 -1.50684300

H 3.85263200 0.14900600 -1.92717400

C 6.90380200 -0.38428600 -0.51123200

H 7.30912100 -0.41776400 1.60800700

H 6.20866500 -0.30881000 -2.55391600

H 7.93303300 -0.58999300 -0.78976000

C 2.30622500 2.26317300 0.35537400

C 2.14429700 2.72527200 -0.95588800

C 2.30116600 3.12883800 1.45117400

C 1.96125000 4.09175000 -1.16316500

H 2.15008000 2.03447900 -1.79136500

C 2.11448700 4.49383400 1.22432000

H 2.42842500 2.73446200 2.45332800

C 1.94684500 4.97276300 -0.07654700

H 1.83298800 4.46826900 -2.17347300

H 2.10260900 5.17990400 2.06558300

H 1.80759200 6.03618400 -0.24704500

C 1.78281300 -1.63316700 -0.54066100

C 1.60728300 -2.08262600 -1.85660200

C 2.09010400 -2.55009900 0.47402200

C 1.74398500 -3.43779700 -2.15551600

H 1.36288100 -1.36514600 -2.63515000

C 2.24493000 -3.90188400 0.15983000

H 2.19642700 -2.21212100 1.49989100

C 2.07088800 -4.35112100 -1.15006700

H 1.60287100 -3.77716700 -3.17767500

H 2.49160700 -4.60618800 0.94925400

H 2.18498200 -5.40491100 -1.38587400

**XI**

Zero-point correction= 0.571367 (Hartree/Particle)

Thermal correction to Energy= 0.609924

Thermal correction to Enthalpy= 0.610868

Thermal correction to Gibbs Free Energy= 0.491144

Sum of electronic and zero-point Energies= -2642.663009

Sum of electronic and thermal Energies= -2642.624452

Sum of electronic and thermal Enthalpies= -2642.623508

Sum of electronic and thermal Free Energies= -2642.743232

1 1

S -2.79967900 0.30361600 0.81693800

N -1.86606000 -0.29332200 -0.34510200

O -2.73928300 -0.23812200 2.19349400

S 2.85177300 -0.88434100 -0.45831800

N 1.88998400 0.38196300 -0.21237300

O 2.92684800 -1.52761400 -1.78859500

Cu 0.01121000 0.04037700 -0.23431200

C -2.33575000 2.03694600 0.87095800

C -2.32258800 2.64016700 2.13092200

C -2.06200500 2.75533200 -0.29818700

C -2.01630800 3.99921500 2.21830800

H -2.53732000 2.05149800 3.01601100

C -1.75659800 4.11147500 -0.19108400

H -2.07179100 2.26450100 -1.26464200

C -1.73667000 4.73133400 1.06230900

H -1.99897800 4.48309500 3.19004100

H -1.53357200 4.68230200 -1.08707400

H -1.50392800 5.78952600 1.13639700

C -4.51025200 0.21423500 0.26205000

C -5.47835700 -0.10726700 1.21561200

C -4.84175500 0.49732100 -1.06670800

C -6.81663900 -0.14625200 0.82132500

H -5.18307800 -0.32902500 2.23502700

C -6.18298900 0.45337300 -1.44226400

H -4.07316100 0.72850900 -1.79542300

C -7.16679400 0.13444900 -0.50089800

H -7.58238600 -0.39791100 1.54853100

H -6.45913900 0.66335400 -2.47095400

H -8.20971000 0.10256300 -0.80178600

C 4.52322800 -0.41583600 0.02013200

C 5.57194800 -0.88546400 -0.77333600

C 4.74649700 0.35712900 1.16390400

C 6.88072000 -0.56797500 -0.40675000

H 5.36009400 -1.47615800 -1.65747600

C 6.05971000 0.66362000 1.51511800

H 3.91749500 0.72425000 1.75819300

C 7.12291300 0.20121100 0.73306200

H 7.70799400 -0.92182400 -1.01413300

H 6.25265800 1.26693800 2.39683000

H 8.14337100 0.44436400 1.01375200

C 2.25612500 -2.09187700 0.72748100

C 1.86118200 -1.71670500 2.01671900

C 2.25727400 -3.42516900 0.31121200

C 1.44746200 -2.71077100 2.90202100

H 1.86123200 -0.67472700 2.31526000

C 1.84013400 -4.40641000 1.21206300

H 2.56330200 -3.67829300 -0.69781300

C 1.43868700 -4.05078400 2.50121300

H 1.13022700 -2.43774600 3.90371800

H 1.82855500 -5.44686700 0.90265400

H 1.11724600 -4.81909600 3.19812400

C 2.30093400 1.65404200 -0.76864400

C 2.19392600 2.78071000 0.05743900

C 2.75019000 1.79869800 -2.08892600

C 2.53823300 4.04072100 -0.43150900

H 1.84085400 2.65817600 1.07754800

C 3.10844400 3.06219900 -2.56347700

H 2.81171700 0.93209900 -2.73946200

C 3.00266300 4.18528800 -1.74124900

H 2.45402100 4.90846300 0.21678900

H 3.46295200 3.16609600 -3.58500100

H 3.27854800 5.16569400 -2.11802700

C -2.21844800 -1.57707000 -0.91352600

C -2.08623200 -1.71685300 -2.30180900

C -2.63623000 -2.66865900 -0.13993800

C -2.37396700 -2.93755900 -2.91127900

H -1.75637600 -0.86630200 -2.89208400

C -2.94161600 -3.88055000 -0.76292600

H -2.71115200 -2.57462100 0.93871900

C -2.80989700 -4.02157000 -2.14518600

H -2.26481100 -3.03746700 -3.98736900

H -3.27332300 -4.72026000 -0.15875500

H -3.04112700 -4.96922500 -2.62222700

**XII**

Zero-point correction= 0.409509 (Hartree/Particle)

Thermal correction to Energy= 0.438508

Thermal correction to Enthalpy= 0.439452

Thermal correction to Gibbs Free Energy= 0.342307

Sum of electronic and zero-point Energies= -2180.623593

Sum of electronic and thermal Energies= -2180.594594

Sum of electronic and thermal Enthalpies= -2180.593650

Sum of electronic and thermal Free Energies= -2180.690795

1 3

S 2.35527500 0.26770000 -0.27763200

N 1.78923700 -0.79398500 -1.34293600

O 1.65067900 1.56110000 -0.08232500

S -2.18670400 0.13127400 0.36351500

N -1.01336100 0.17369800 -0.73021200

O -1.90457600 -0.25978600 1.75961200

H -0.36083400 0.93691000 -0.51290300

H 2.04245900 -0.49793500 -2.28988700

Cu -0.10177800 -1.52667400 -1.30420400

C -2.90428100 1.77849300 0.37312300

C -3.38631900 2.25751500 1.59433000

C -2.98456700 2.52897100 -0.80489000

C -3.96452200 3.52667800 1.63019000

H -3.29504000 1.65336200 2.49016400

C -3.56529000 3.79512000 -0.74826400

H -2.58956000 2.14145300 -1.73755900

C -4.05535300 4.29038100 0.46398600

H -4.34097200 3.91750000 2.57041600

H -3.63222300 4.39519000 -1.65048000

H -4.50695500 5.27727300 0.49924200

C -3.37416400 -1.01095500 -0.33878600

C -3.65709800 -1.00930200 -1.70985100

C -4.02922100 -1.86139300 0.55486300

C -4.61619900 -1.89887800 -2.19041800

H -3.13229900 -0.34322700 -2.38480000

C -4.98428000 -2.74670400 0.05337200

H -3.77936100 -1.83882700 1.60982500

C -5.27641700 -2.76422000 -1.31215200

H -4.84388400 -1.91923100 -3.25159700

H -5.49637800 -3.42254100 0.73105700

H -6.02120600 -3.45504400 -1.69575300

C 2.33534400 -0.64041800 1.26117500

C 2.13577500 0.10095400 2.42937200

C 2.54754500 -2.02368900 1.28128500

C 2.14314400 -0.56987800 3.65179200

H 1.95962600 1.16957800 2.37642300

C 2.54894600 -2.67496000 2.51425100

H 2.68685100 -2.57657800 0.35953100

C 2.35025700 -1.95084500 3.69342400

H 1.98101800 -0.01315800 4.56929400

H 2.70215700 -3.74897500 2.55124400

H 2.35363400 -2.46679900 4.64885300

C 4.05260600 0.61475100 -0.74560000

C 4.48805700 1.93959400 -0.65859900

C 4.88839600 -0.42173400 -1.17754500

C 5.80370800 2.22981800 -1.02218800

H 3.80829300 2.71670400 -0.32730600

C 6.19876200 -0.10995600 -1.53503300

H 4.52511600 -1.44188500 -1.24255100

C 6.65410700 1.21030100 -1.45636900

H 6.16079700 3.25342500 -0.96695400

H 6.86304200 -0.89770800 -1.87637800

H 7.67658600 1.44409800 -1.73715800

**XIII**

Zero-point correction= 0.405226 (Hartree/Particle)

Thermal correction to Energy= 0.434986

Thermal correction to Enthalpy= 0.435930

Thermal correction to Gibbs Free Energy= 0.336204

Sum of electronic and zero-point Energies= -2180.619366

Sum of electronic and thermal Energies= -2180.589606

Sum of electronic and thermal Enthalpies= -2180.588662

Sum of electronic and thermal Free Energies= -2180.688388

1 3

S -2.97340400 0.05887500 -1.02351300

N -1.72016700 1.00188200 -0.66607900

O -3.09488600 -0.56842700 -2.35837400

S 2.78754400 -0.30812800 0.35209400

N 1.78263000 -0.40654300 -0.89212700

O 2.44418800 -0.88782100 1.66671900

H 1.84876600 -1.34186200 -1.29983100

H -1.85300900 1.90302800 -1.13260400

Cu 0.02580700 0.28750800 -0.76170300

C -2.92235200 -1.20731600 0.23166100

C -3.39377300 -2.47520500 -0.13069000

C -2.52406500 -0.91025300 1.54241300

C -3.43712700 -3.47391600 0.84100500

H -3.69338200 -2.66992700 -1.15475200

C -2.57638700 -1.92333300 2.49873800

H -2.16407500 0.07990500 1.79600600

C -3.03123000 -3.19892700 2.15018300

H -3.78420200 -4.46734100 0.57394800

H -2.25747000 -1.71688400 3.51581800

H -3.06843400 -3.98162000 2.90206500

C -4.43572100 1.24027800 -0.84828400

C -5.63515400 0.76439700 -1.54573800

C -4.58690800 1.88488900 0.45998400

C -6.71047000 0.42890400 -0.76832400

H -5.61555800 0.59974200 -2.61716900

C -5.68138900 1.53205300 1.20297300

H -3.82138900 2.55261600 0.84143500

C -6.73188100 0.75506700 0.62349300

H -7.58939600 -0.02302800 -1.22033400

H -5.79609000 1.90623800 2.21694200

H -7.61033500 0.51907700 1.21480300

C 4.32496000 -1.05081000 -0.20631700

C 5.06092400 -1.78058400 0.73077100

C 4.75834600 -0.88613500 -1.52687900

C 6.26186600 -2.36495600 0.32678100

H 4.68790300 -1.89757500 1.74222700

C 5.96080000 -1.47744800 -1.91151400

H 4.16782400 -0.32056400 -2.23962900

C 6.70999700 -2.21221800 -0.98731700

H 6.84338900 -2.94176100 1.03918700

H 6.31095500 -1.36552100 -2.93299100

H 7.64571200 -2.66932300 -1.29492400

C 3.03490500 1.45509500 0.55208000

C 3.07832500 2.30837100 -0.55610400

C 3.22352500 1.92148000 1.85551700

C 3.30860700 3.66669400 -0.34118400

H 2.92233000 1.92178400 -1.55682500

C 3.45258900 3.28410800 2.05053400

H 3.17479200 1.23218400 2.69132800

C 3.49654500 4.15187900 0.95689700

H 3.34206100 4.34529000 -1.18804900

H 3.59541500 3.66503000 3.05699000

H 3.67826900 5.21076900 1.11505000

**XIV**

Zero-point correction= 0.490057 (Hartree/Particle)

Thermal correction to Energy= 0.523950

Thermal correction to Enthalpy= 0.524894

Thermal correction to Gibbs Free Energy= 0.416636

Sum of electronic and zero-point Energies= -2411.588550

Sum of electronic and thermal Energies= -2411.554657

Sum of electronic and thermal Enthalpies= -2411.553713

Sum of electronic and thermal Free Energies= -2411.661971

1 3

S -2.00174500 0.57090200 0.35592200

N -1.66551300 -0.65692800 -0.63171200

O -1.29613900 0.68210900 1.66058000

S 2.57778400 0.51505500 0.17734100

N 1.25147900 -0.37955500 0.21117000

O 2.51561700 1.95815000 -0.13628100

H 0.67245100 -0.10117700 1.01156400

Cu 0.20080300 -0.73636200 -1.49097500

C 3.34432900 0.33098400 1.79390400

C 4.02972800 1.43440100 2.30897800

C 3.26077700 -0.88117200 2.48719900

C 4.64601600 1.31483100 3.55500300

H 4.06315000 2.36272600 1.74957100

C 3.88311000 -0.98155600 3.73077600

H 2.71123900 -1.71925300 2.07253200

C 4.57478600 0.11166800 4.26115300

H 5.17915900 2.16318800 3.97289400

H 3.82567500 -1.91283800 4.28586700

H 5.05739600 0.02519900 5.23008400

C 3.61166600 -0.30446700 -1.03440000

C 3.66575200 -1.70074400 -1.11367800

C 4.38773300 0.51692500 -1.85487200

C 4.51581600 -2.27990400 -2.05404600

H 3.04826700 -2.31729200 -0.47085400

C 5.23059500 -0.08190400 -2.79218000

H 4.31394600 1.59542900 -1.76973600

C 5.29460500 -1.47345800 -2.89010500

H 4.56531500 -3.36115300 -2.13715500

H 5.83376300 0.54076600 -3.44550300

H 5.95304600 -1.93334400 -3.62096000

C -1.59992200 2.00404300 -0.63921900

C -1.05946900 3.10326300 0.03256700

C -1.86270700 2.02462600 -2.01419200

C -0.76759300 4.25345100 -0.70082900

H -0.85699700 3.04901500 1.09617900

C -1.56359400 3.18407800 -2.72815600

H -2.27026200 1.15486900 -2.51565400

C -1.02022800 4.29367900 -2.07360700

H -0.33758800 5.11370100 -0.19789100

H -1.75132100 3.21700500 -3.79680000

H -0.79037400 5.19225600 -2.63824300

C -3.77207300 0.57464100 0.67248300

C -4.19192900 0.88478700 1.96785400

C -4.67384900 0.30288100 -0.36147400

C -5.56231100 0.92124900 2.23011400

H -3.46259600 1.08310900 2.74522600

C -6.03753800 0.34410300 -0.07868800

H -4.32419000 0.05160700 -1.35662900

C -6.47958200 0.65363600 1.21179000

H -5.90940500 1.15766800 3.23114200

H -6.75461400 0.13132500 -0.86538600

H -7.54424400 0.68384600 1.42298300

C -2.11660300 -1.97376400 -0.29332200

C -2.52863300 -2.78607200 -1.36436600

C -2.10408100 -2.49332200 1.01301000

C -2.93238700 -4.09855800 -1.12791100

H -2.53139000 -2.37382300 -2.36866600

C -2.52949000 -3.80123200 1.23850700

H -1.76157000 -1.88108700 1.84091200

C -2.94033500 -4.60800600 0.17317400

H -3.24916700 -4.72013800 -1.96007300

H -2.53005400 -4.19514600 2.25064600

H -3.26267000 -5.62847900 0.35664800

**XV**

Zero-point correction= 0.570304 (Hartree/Particle)

Thermal correction to Energy= 0.609078

Thermal correction to Enthalpy= 0.610022

Thermal correction to Gibbs Free Energy= 0.491931

Sum of electronic and zero-point Energies= -2642.546570

Sum of electronic and thermal Energies= -2642.507796

Sum of electronic and thermal Enthalpies= -2642.506852

Sum of electronic and thermal Free Energies= -2642.624944

1 3

S 2.15540200 0.46841400 -0.75168100

N 1.79285300 -0.13924500 0.69726200

O 1.64303400 -0.14045500 -1.99886000

S -2.39928400 0.31448500 -0.44426900

N -1.45265900 -0.65900800 0.41833600

O -1.91072000 0.94657800 -1.69056200

Cu -0.00374700 0.16419400 1.64674400

C -3.91087400 -0.58676800 -0.83376700

C -4.46382900 -0.38475400 -2.09989800

C -4.50979000 -1.42189500 0.11455000

C -5.65355400 -1.04065600 -2.42041300

H -3.96643100 0.26332900 -2.81266400

C -5.69738800 -2.06773900 -0.22337900

H -4.05480000 -1.57991200 1.08568500

C -6.26799100 -1.87633700 -1.48584000

H -6.09635000 -0.89769600 -3.40126500

H -6.17461000 -2.72402100 0.49781800

H -7.19354600 -2.38369500 -1.74098700

C -2.83694600 1.61214700 0.71749400

C -3.15740800 1.32035400 2.04871400

C -2.91322700 2.90846700 0.20503700

C -3.55680700 2.36413700 2.88186600

H -3.08462100 0.30869200 2.42921800

C -3.31081500 3.94151400 1.05601800

H -2.65335300 3.09781100 -0.83017100

C -3.63302300 3.66975200 2.38709600

H -3.80173800 2.15695700 3.91887100

H -3.37226900 4.95626600 0.67502100

H -3.94672300 4.47662400 3.04276800

C 1.55348100 2.15531300 -0.64508900

C 0.96848600 2.68077100 -1.79856200

C 1.73780200 2.92137400 0.51210700

C 0.55747100 4.01491600 -1.78905600

H 0.82608000 2.05206000 -2.66963400

C 1.31861000 4.25108200 0.50259900

H 2.18657800 2.49094500 1.39949400

C 0.73556200 4.79654300 -0.64563500

H 0.10179700 4.44010200 -2.67804300

H 1.45032100 4.85971900 1.39184600

H 0.42230000 5.83655300 -0.64787300

C 3.95320300 0.54407400 -0.86720100

C 4.52140800 0.27977600 -2.11521500

C 4.72812800 0.88095600 0.24699600

C 5.90884800 0.35851300 -2.24576500

H 3.88979300 0.01131400 -2.95461700

C 6.11159600 0.95479500 0.09687100

H 4.26798000 1.06372800 1.21143000

C 6.69946100 0.69636100 -1.14556400

H 6.36852800 0.15339500 -3.20763800

H 6.73066600 1.20955300 0.95153700

H 7.77832200 0.75577500 -1.25363800

C 2.36824400 -1.39238200 1.07865900

C 2.77908000 -1.51519600 2.41929700

C 2.48607800 -2.49121500 0.20986300

C 3.30737700 -2.71934300 2.87926100

H 2.68682500 -0.65885000 3.08038100

C 3.03628000 -3.68299800 0.67732000

H 2.13316300 -2.41600600 -0.81254000

C 3.44491300 -3.80415900 2.00866800

H 3.62370000 -2.80574600 3.91462400

H 3.13390200 -4.52596300 -0.00040100

H 3.86705500 -4.73876600 2.36585500

C -1.20852100 -1.98288600 -0.07434400

C -1.23988100 -3.02546900 0.86670200

C -0.91605300 -2.26534200 -1.41911600

C -0.99848900 -4.33760400 0.46339600

H -1.46414600 -2.79545500 1.90437300

C -0.69830300 -3.58491800 -1.81423800

H -0.84125800 -1.45788800 -2.13891600

C -0.73780100 -4.62328500 -0.87927400

H -1.02698700 -5.13782200 1.19719300

H -0.48706800 -3.79980300 -2.85792400

H -0.56417500 -5.64782200 -1.19492900

**1**

Zero-point correction= 0.202858 (Hartree/Particle)

Thermal correction to Energy= 0.215514

Thermal correction to Enthalpy= 0.216458

Thermal correction to Gibbs Free Energy= 0.162434

Sum of electronic and zero-point Energies= -991.756423

Sum of electronic and thermal Energies= -991.743768

Sum of electronic and thermal Enthalpies= -991.742823

Sum of electronic and thermal Free Energies= -991.796847

0 1

S 0.01222400 1.31546300 0.13455200

O -0.05295800 2.12030300 -1.11289800

N 0.16224600 1.91557000 1.55661100

H -0.64290200 2.50994400 1.76760800

C 1.43612200 0.22280300 0.02232000

C 1.90854600 -0.08588700 -1.25386800

C 2.04298000 -0.28848600 1.17062500

C 3.00666200 -0.93696800 -1.38102500

H 1.43340900 0.35412400 -2.12426200

C 3.14225800 -1.13554600 1.02920100

H 1.66956100 0.00005100 2.14630000

C 3.61985800 -1.46330500 -0.24226400

H 3.38704700 -1.18121500 -2.36879400

H 3.62938900 -1.53511100 1.91426800

H 4.47630700 -2.12414700 -0.34494200

C -1.42633700 0.19810400 0.03582400

C -2.13186500 0.08979800 -1.16347800

C -1.81841600 -0.50903100 1.17449100

C -3.24511300 -0.75027900 -1.22197900

H -1.81396800 0.67062900 -2.02251800

C -2.93053800 -1.34816200 1.10458300

H -1.26590400 -0.38810700 2.10128600

C -3.64115800 -1.46993700 -0.09243300

H -3.80513700 -0.83875500 -2.14885600

H -3.24496200 -1.90221100 1.98471200

H -4.50847600 -2.12262800 -0.14271000

**3**

Zero-point correction= 0.283945 (Hartree/Particle)

Thermal correction to Energy= 0.301340

Thermal correction to Enthalpy= 0.302284

Thermal correction to Gibbs Free Energy= 0.236102

Sum of electronic and zero-point Energies= -1222.727190

Sum of electronic and thermal Energies= -1222.709795

Sum of electronic and thermal Enthalpies= -1222.708850

Sum of electronic and thermal Free Energies= -1222.775032

0 1

S 0.36465300 0.28691500 0.71797900

O 0.40876200 0.10806500 2.19368500

N -0.55964300 1.29436000 -0.01997900

C 0.15424800 -1.39623500 0.04333300

C -0.17298500 -1.55711600 -1.30439800

C 0.31781800 -2.49325500 0.88958400

C -0.33006500 -2.84599000 -1.81336700

H -0.31821600 -0.68706600 -1.93650300

C 0.15892600 -3.77853600 0.36884800

H 0.54988400 -2.33041600 1.93647900

C -0.16135200 -3.95410500 -0.97901000

H -0.59012900 -2.98441000 -2.85895000

H 0.28024600 -4.64129300 1.01792900

H -0.28601800 -4.95637200 -1.37968400

C 1.99970400 0.80758800 0.17844400

C 3.07554700 0.47787700 1.00408200

C 2.17975800 1.50532300 -1.01672900

C 4.36300100 0.84897700 0.61520100

H 2.89761400 -0.03829200 1.94136100

C 3.47207500 1.87278400 -1.39111000

H 1.31681200 1.77218000 -1.61521000

C 4.56116400 1.54174100 -0.58076600

H 5.20856500 0.60369900 1.25137000

H 3.62684300 2.42349800 -2.31461100

H 5.56516200 1.83103900 -0.87887100

C -1.96805100 1.19527400 0.03058000

C -2.67302100 1.83029900 -1.00788100

C -2.70227300 0.55250200 1.04510100

C -4.06448400 1.80873200 -1.04148500

H -2.10511900 2.33931100 -1.78148700

C -4.09626200 0.52984000 0.99735800

H -2.18269500 0.09238700 1.87963600

C -4.78795500 1.15296600 -0.04189900

H -4.58668900 2.30654800 -1.85503100

H -4.64415100 0.02591200 1.78996700

H -5.87391500 1.13560900 -0.06901300
